# Histo-Blood Group Antigens Act as Attachment Factors of Rabbit Hemorrhagic Disease Virus Infection in a Virus Strain-Dependent Manner

**DOI:** 10.1371/journal.ppat.1002188

**Published:** 2011-08-25

**Authors:** Kristina Nyström, Ghislaine Le Gall-Reculé, Paola Grassi, Joana Abrantes, Nathalie Ruvoën-Clouet, Beatrice Le Moullac-Vaidye, Ana M. Lopes, Pedro J. Esteves, Tanja Strive, Stéphane Marchandeau, Anne Dell, Stuart M. Haslam, Jacques Le Pendu

**Affiliations:** 1 INSERM, U892, Université de Nantes, Nantes, France; 2 Anses, Laboratoire de Ploufragan/Plouzané, Unité de Virologie, Immunologie, Parasitologie Aviaires et Cunicoles, Ploufragan, France; 3 Division of Molecular Biosciences, Faculty of Natural Sciences, Imperial College London, London, United Kingdom; 4 CIBIO, Centro de Investigacao em Biodiversidade e Recursos Geneticos, Campus Agrario de Vairao, Vairao, Portugal; 5 CITS, Centro de Investigacao em Tecnologias de Saude, CESPU, Gandra, Portugal; 6 Commonwealth Scientific and Industrial Research Organisation, Canberra, Australian Capital Territory, Australia; 7 Invasive Animals Cooperative Research Centre, Canberra, Australia; 8 Office National de la Chasse et de la Faune Sauvage, Direction des Etudes et de la Recherche, Nantes, France; University of North Carolina at Chapel Hill, United States of America

## Abstract

Rabbit Hemorrhagic disease virus (RHDV), a calicivirus of the *Lagovirus* genus, and responsible for rabbit hemorrhagic disease (RHD), kills rabbits between 48 to 72 hours post infection with mortality rates as high as 50–90%. Caliciviruses, including noroviruses and RHDV, have been shown to bind histo-blood group antigens (HBGA) and human non-secretor individuals lacking ABH antigens in epithelia have been found to be resistant to norovirus infection. RHDV virus-like particles have previously been shown to bind the H type 2 and A antigens. In this study we present a comprehensive assessment of the strain-specific binding patterns of different RHDV isolates to HBGAs. We characterized the HBGA expression in the duodenum of wild and domestic rabbits by mass spectrometry and relative quantification of A, B and H type 2 expression. A detailed binding analysis of a range of RHDV strains, to synthetic sugars and human red blood cells, as well as to rabbit duodenum, a likely gastrointestinal site for viral entrance was performed. Enzymatic cleavage of HBGA epitopes confirmed binding specificity. Binding was observed to blood group B, A and H type 2 epitopes in a strain-dependent manner with slight differences in specificity for A, B or H epitopes allowing RHDV strains to preferentially recognize different subgroups of animals. Strains related to the earliest described RHDV outbreak were not able to bind A, whereas all other genotypes have acquired A binding. In an experimental infection study, rabbits lacking the correct HBGA ligands were resistant to lethal RHDV infection at low challenge doses. Similarly, survivors of outbreaks in wild populations showed increased frequency of weak binding phenotypes, indicating selection for host resistance depending on the strain circulating in the population. HBGAs thus act as attachment factors facilitating infection, while their polymorphism of expression could contribute to generate genetic resistance to RHDV at the population level.

## Author Summary

Rabbit hemorrhagic disease virus (RHDV), detected as late as 1984, has spread to large parts of the world, threatening rabbit populations and other species dependent on rabbits in many European countries. Mortality has been shown to be as high as 90% and rabbits are killed 48 to 72 hours after infection. Related viruses called noroviruses, infect humans in a manner dependent on the expression of histo-blood group antigens (HBGAs), which are not only expressed on red blood cells, but also on epithelial cells, in saliva and on mucins of the intestinal tract. RHDV also binds to HBGA and in this report we characterize binding of strains of all genetic groups of RHDV to different HBGAs. We also demonstrate HBGAs to function as attachment factors in a challenge experiment. As polymorphisms of genes involved in HBGA synthesis divide the rabbit population into different subgroups, we find selection of low-binding subgroups of wild rabbits in populations recovering from devastating outbreaks of RHDV. This is the first demonstration of differential HBGA specificities of RHDV strains, description of function in infection and demonstration of host selection due to RHDV infection based on HBGA phenotype.

## Introduction

Rabbit hemorrhagic disease virus (RHDV), a single stranded positive-sense RNA virus belonging to the *Lagovirus* genus of the *Caliciviridae* family, is the cause of rabbit hemorrhagic disease (RHD), a disease affecting wild and domestic rabbits of the *Oryctolagus cuniculus* species. RHD was first described in Angora rabbits in China in 1984. By 1987 RHD was detected in Czechoslovakia and Italy, and rapidly expanded to most European countries [1]. RHDV usually kills rabbits within 48 to 72 hours of infection. The disease is characterized by acute necrotizing hepatitis and haemorrhages, sometimes preceded by tracheitis and generally associated with disseminated intravascular coagulation in many organs, particularly the lungs, heart and kidneys. There are three different clinical courses of RHD, the peracute form is distinguished by sudden death with no previous clinical signs. The acute form of RHD involves depression, anorexia, apathy, rapid respiration, anemia and some animals show signs of abdominal distress. Animals perish after one to three days. The sub acute form involves slight clinical symptoms and the animals recover within 2–3 days [2], [3]. Mortality rates are as high as 50–90% although rates are lower in young animals less than 6–8 weeks-old, and no mortality occurs in animals less than 4 weeks-old. Kittens can become infected and shed virus but do not show clinical signs of the disease. The most common routes of infection are the oral and upper respiratory routes, mainly through direct contact between animals or through contact with water or contaminated food. The virus is present in the blood, organs, secretions and skin or fur of infected animals. It is excreted in large amounts through urine and feces and can also be spread by insects [4]. In addition RHDV is resistant in the environment particularly in dry conditions and to date there is no evidence that RHDV can infect other species [5]. RHDV has become endemic in the original distribution range of the rabbit, Spain, Portugal and France, where it has caused severe long term decline of rabbit population size [6], [7]. The drastic decline also threatens species dependent on rabbits such as the Iberian lynx (*Lynx pardinus*) and the Spanish imperial eagle (*Aquila adalberti)*, which are specialist predators, and to a lesser extent the Bonelli eagle (*Hieraaetus fasciatus*). The decline of wild rabbit populations has also had an impact on lizard populations, which use rabbit warrens during hot summer periods [8]. In addition, RHDV can cause devastating losses for rabbit producers, although efficient vaccines are commercially available allowing protection of farmed rabbits [9].

Anti-RHDV antibodies have been detected in wild rabbit serum sampled prior to the reported emergence of RHDV [10]. This has led to speculation that non-pathogenic RHDV strains may have been circulating in rabbit populations prior to the first detected RHDV outbreak. Such strains were indeed discovered later in countries such as Italy, France and Australia [11], [12], [13], [14]. Infection by these non-pathogenic strains has been reported to confer complete protection [11], partial protection [15] or no protection [12], [16] against RHDV in rabbits through cross-recognizing antibodies.

**Figure 1:**
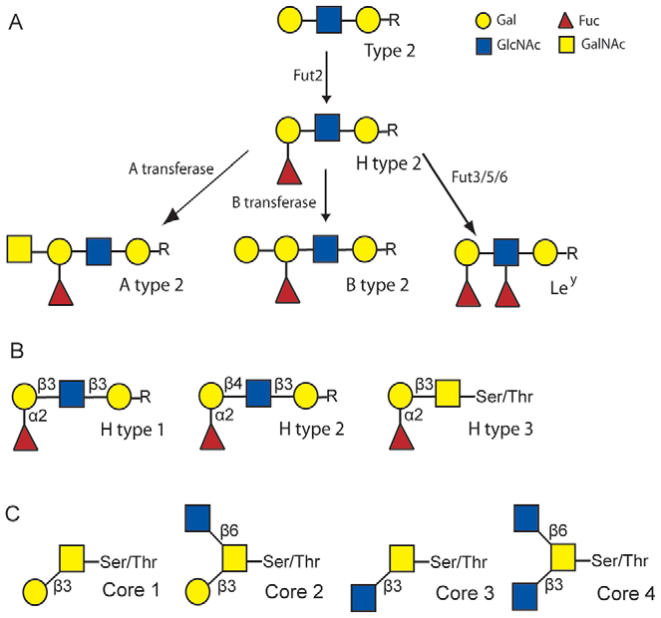
Schematic of the synthesis of rabbit HBGA ligands. (A) Schematic of the synthesis of H type 2, A type 2, B type 2 and Le^y^. (B) Structural representation of H type 1, H type 2 and H type 3. (C) Structural representation of core structures of O-glycans. The symbol presentation of O-glycans is based on the nomenclature used in Essentials of Glycobiology textbook (http://www.ncbi.nlm.nih.gov/books/NBK1908) and adopted by the Consortium for Functional Glycomics (http://www.functionalglycomics.org).

The genetic diversity between RHDV isolates is quite low even between isolates that are not geographically correlated. Indeed, the nucleotide and amino acid differences between strains range between 1–10% and 1–6%, respectively, which is far lower than the differences observed for other caliciviruses [12], [16]. Nevertheless, it has been suggested that French RHDV isolates can be assigned into six genetic groups, G1 to G6 following spatio-temporal distribution [17]. In France, G1 has almost completely disappeared and is found exclusively in the south-west near the Spanish border. G2, in which had been included the strain isolated in the first reported outbreak in China in 1984, has not been isolated recently. The genetic group G4 emerged from G3, while G5 and G6 appeared as new independent groups [17], the latter corresponding to the first antigenic variant identified, RHDVa [18]. More recently, the pathogenic forms of RHDV were shown to cluster into four major groups [19], [20] with the genetic groups G3, G4 and G5 as an artificial subdivision of Group 4 identified by Kerr and co-workers, which clusters Western Europe and Bahrain strains collected from 1989 onward. RHDV has previously been shown to bind the oligosaccharide H type 2 and A type 2 (Fig 1A), histo-blood group antigens (HBGAs) expressed on the duodenal surface and trachea of rabbits, two possible doors of entry for the virus [21]. HBGAs are polymorphic carbohydrate structures representing terminal exposed portions of larger glycans O- or N-linked to proteins or to glycolipids. In many vertebrate species they are mainly expressed on epithelial cells and only a few primate species, including humans, express them on vascular endothelial cells and erythrocytes. They are synthesized by stepwise addition of monosaccharide units from several precursors by specific glycosyltransferases (Fig. 1). The recent occurrence of the highly pathogenic RHDV with a documented HBGA binding ability can be expected to provide a useful model to study the impact of the virus on the host's HBGA diversity and reciprocally of the host diversity on the virus HBGA-binding properties. We have here determined host diversity regarding HBGA expression on the duodenum surface, a likely point of entry for the virus, and strain-specific binding of RHDV by examining the binding to synthetic sugars, haemagglutination and binding to the duodenal mucosa of both wild and domestic rabbits. The role of each glycan for binding was determined through specific enzymatic removal of the monosaccharide comprising the A, B or H epitopes. Host variation was determined through semi-quantitative A, B and H phenotyping of rabbit duodenums and structural characterization of glycans by mass spectrometry. The role of HBGA binding in RHDV infection was further tested by challenging AB negative and AB positive rabbits with a strain largely dependent on AB binding, revealing an important role of HBGA binding at lower dose infections. In addition, preliminary evidence for selection of weak-binding ABH phenotypes were detected in wild rabbit populations following RHDV outbreaks. Understanding the selection of weak-binding RHDV phenotypes is important as resistance to infection generates problems for controlling the large rabbit population in Australia and provides possibilities of selecting RHDV resistant animals in areas where the rabbit populations are threatened.

## Results

### Phylogenetic relationships between the strains used

**Figure 2:**
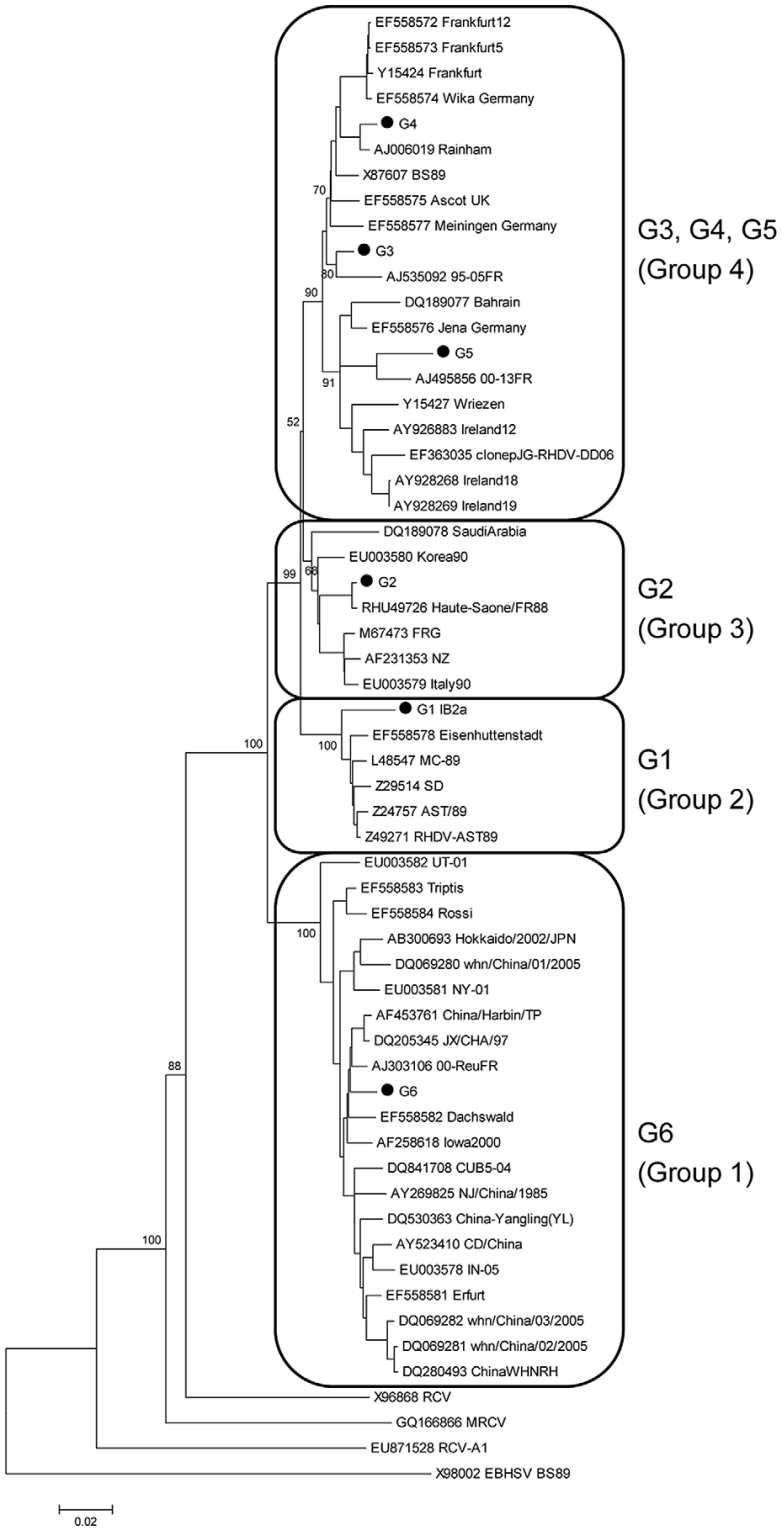
Evolutionary tree of RHDV strains and non-pathogenic rabbit calicivirus strains (RCV, RCV-A1 and MRCV) according to nucleotide sequences of the gene encoding the capsid protein VP60. The 6 RHDV strains used in this study are marked with a dot (GenBank accession numbers: G1 JF438967, G2 FR823355, G3 FR823354, G4 AJ535094, G5 AM085133, and G6 AJ969628). The genetic group of each strain (G1 to G6 according to Le Gall-Reculé et al.[17] and Group 1 to 4 according to Kerr et al. [20]) is annotated. For the G1 strain, the genetic group is noted as defined in Muller et al. [19] (IB2a). Capsid sequences where recombination was previously detected were excluded from the analyses [76], [77]. The newly obtained sequences were checked for recombination using RDP3 [78]. Evolutionary distances were computed using the p-distance method. Alignment gaps and missing data were eliminated only in pairwise sequence comparisons (Pairwise deletion option). The *European brown hare syndrome virus* strain (GenBank accession number X98002) was used as an outgroup to root the tree. The tree was constructed using the neighbor-joining method [79] with the pairwise deletion option. Reliability of the tree was assessed by bootstrap with 1000 replicates and is indicated in the nodes for relevant clusters. Several genetic distance methods were used and similar results were obtained, but only p-distance is shown. The phylogenetic analyses were conducted in MEGA 4 [80]. Similar trees were obtained using the Maximum Likelihood (ML) method as implemented in Garli v1.0 [81].

The strains used in this study were chosen to represent each of the six chronologically established genetic groups (G1–G6) previously identified by Le Gall-Reculé and co-workers [17]. To analyze the phylogenetic position of these strains, a neighbor-joining tree was constructed using complete nucleotide sequences of the capsid gene. Similar to the finding of Kerr et al. [20], the resulting phylogenetic diagram shows a division of the analyzed strains into four rather than six clades with G3, G4 and G5 forming a major group while G1, G2 and G6 are clearly distinct highly supported groups (Fig 2). G1 corresponds to strains that circulated in Western Europe during the first RHDV outbreaks [17] and now exclusively circulate in the Iberian Peninsula and sporadically in the South of France [19], [22]. G6 corresponds to the antigenic variant strains (RHDVa) first described by Capucci et al [18].

### Binding of RHDV strains to synthetic sugars

**Figure 3:**
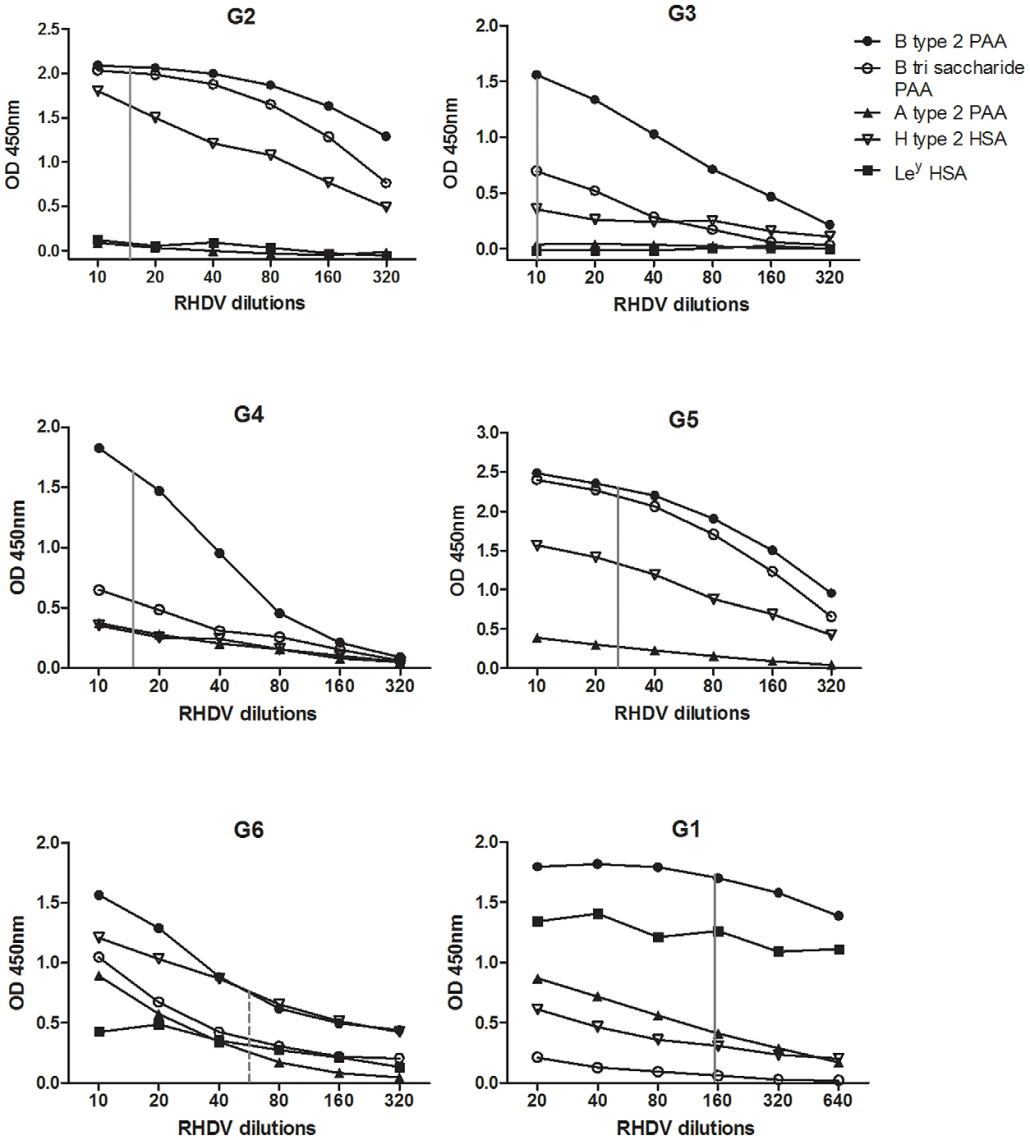
RHDV strains binding to synthetic oligosaccharides. RHDV liver extract binding used in a range of dilutions for detection of binding to human serum albumin (HSA) and polyacrylamide (PAA) conjugated oligosaccharides. Vertical grey line symbolizes the same amount of viral RNA, whereas the dotted line symbolizes the same amount of viral RNA as the solid lines, though detected with a different antibody system since the G6 strain was less well recognized than the other strains by the Lp4 polyclonal antibody.

To address the question of carbohydrate binding of RHDV, six different strains designated G1–G6 were used. RHDV liver extracts with high virus titres, at least 1×10^10^ viral RNA copies, were used to screen a panel of 38 polyacrylamide (PAA)-conjugated oligosaccharides and 19 human serum albumin (HSA)- conjugated oligosaccharides (see Table S1). The carbohydrates with any capacity to bind RHDV were then used to determine binding over a range of RHDV dilutions (Fig 3). The dilution corresponding to equivalent amounts of viral RNA, determined through real time RT-PCR, are shown in the figure with a vertical line. The antigenic variant G6 was detected with a mouse monoclonal antibody 2G3 previously determined to bind G6 strains as well as all other pathogenic RHDV strains (kindly provided by Lorenzo Capucci). G1–G5 were on the other hand detected with a high-titered anti-RHDV rabbit serum and the amount of G6 may therefore not be completely comparable to that of the other strains. All strains showed strongest binding to B type 2. G1 was the only strain showing strong binding to Le^y^ and the binding to H type 2, A type 2 and B trisaccharide varied between the strains tested. Therefore H type 2 is not the only HBGA which may be of relevance for RHDV binding and individual strains show distinct specificities for synthetic oligosaccharides.

### Haemagglutination characteristics of RHDV strains

Human red blood cells (RBC) carry A, B and H type 2 on their surface, all of which may be ligands of the G1–G6 strains tested on synthetic sugars. Therefore the ABH expression in this non-synthetic system was used to test for RHDV strain binding. Moreover, RHDV strains have previously been described to be either haemagglutinating or non-haemagglutinating [23]. We tested all 6 strains of RHDV on human A, B and O blood (Table 1). All RHDV strains were able to agglutinate B blood consistent with B type 2 recognition. The G2 and G3 strains also showed strong binding to H type 2 on O RBC. G1 and G5 showed weak binding to O RBC, while G4 and G6 did not agglutinate O blood. G1 was able to agglutinate A blood to the same extent as B, whilst all other strains showed weak agglutination of A, indicating B type 2 as a ligand for G1–G6, H type 2 as a ligand for G2 and G3 and A as a ligand for G1.

**Table 1:** RHDV agglutination titres of human red blood cells.

	G2	G3	G4	G5	G6	G1
Type O	2048	512	0	8	0	16
Type A	2	32	2	16	16	256
Type B	4096	1024	256	512	128	256

### ABH antigens expression on rabbit tissues

**Figure 4:**
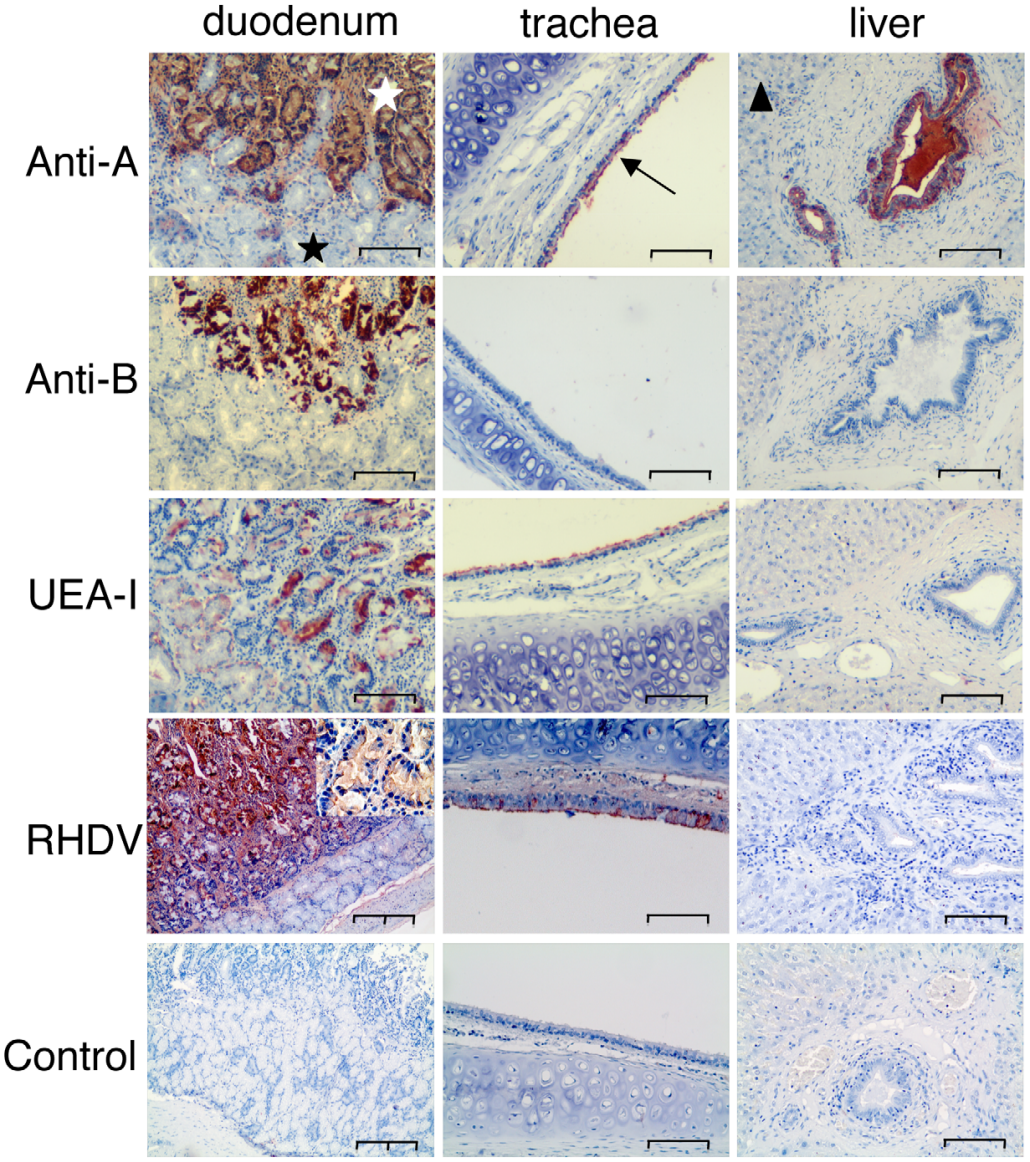
Immunohistochemistry of A, B and H expression and RHDV binding to duodenum and trachea. Staining of the duodenum, trachea and liver of A+B+ rabbits by an anti-A, an anti-B and the H type 2 specific UEA-I lectin. Binding of RHDV (shown a G3 strain) was performed by incubating liver extracts on tissue sections followed by detection using an anti-RHDV monoclonal antibody as described in the “materials and methods” section. Negative controls correspond to section where the liver extracts were omitted. The simple bar corresponds to 100 µm and the double bar to 200 µm. A 4x higher magnification is inserted into the RHDV/duodenum picture, showing the labeling of the crypt epithelial cells. The white star is the Lieberkühn crypts (surface layer of the duodenal mucosa). The black star is the Brünners' glands (deep layer of the duodenal mucosa). The arrow indicates the epithelial layer of the trachea. The triangle indicates the liver parenchyma.

To study HBGAs expression and distribution in the rabbit duodenum and trachea, the proposed sites of viral entry, as well as in the liver, a major site of replication, monoclonal antibodies against A and B as well as *Ulex europaeus* lectin (UEA-I) were used to detect A, B and H type 2 (Le^y^) on wild rabbit tissue sections, respectively (Fig 4). HBGA expression was always restricted to epithelial cells. The nine French wild rabbits tested were found to be either A and B positive (A+B+) at the duodenum surface or A and B negative (A−B−). Staining with UEA-I was much stronger and homogenous on sections from A−B− rabbits (data not shown), suggesting partial masking of H type 2 by the A and B epitopes. In addition, expression of the B antigen appeared to differ from those of the A and H antigens. A, B and H were expressed on the crypts of Lieberkühn (surface layer of the mucosa) and not on the Brünners' glands (deep layer). Yet, staining by the anti-B appeared more patchy and irregular than staining by the anti-A and it was always weaker. In addition, in A+B+ animals, A antigen, but not B antigen was detected on the surface of the trachea and on the biliary ducts of portal spaces in the liver. Neither A, B or H antigen could be detected on the liver parenchyma.

### Analysis of rabbit HBGA diversity

**Figure 5:**
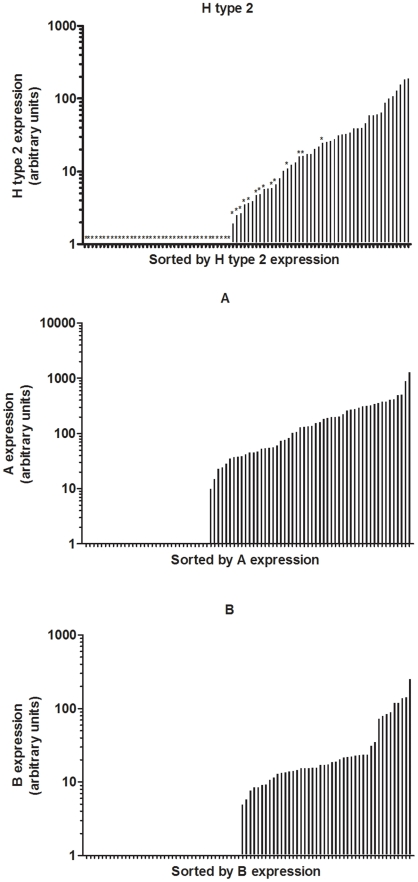
Expression of H type 2, A and B in rabbit duodenum. Relative expression of H type 2, A and B where the individual rabbits are sorted according to H type 2, A and B. A- rabbits are shown with stars in graph of H type 2 expression.

In order to get insights into the diversity of HBGA expression in rabbits, a more quantitative assay was needed. Therefore, fresh rabbit duodenums were collected from 84 rabbits of both wild and laboratory origin for more detailed studies of ABH expression and RHDV binding. A semi-quantitative ELISA system for rabbit duodenum scrapings determined that all animals do express either H type 2, A or B. No animal was found to be of a clear non-secretor phenotype as described for humans, and all rabbits expressed detectable levels of either H type 2 or A and/or B, albeit with great individual variations and where A expression generally was stronger than B. Ranking animals by increasing H type 2 detection clearly showed an inverse relationship with A expression and to a lesser degree with B expression, indicating that A and B epitopes mask the H motif (Fig 5 and Table S2). Based on the detection of the A or B antigens, animals were phenotyped A+B+, A−B− or A+B− with the respective frequencies 0.52, 0.38 and 0.1 respectively.

**Figure 6:**
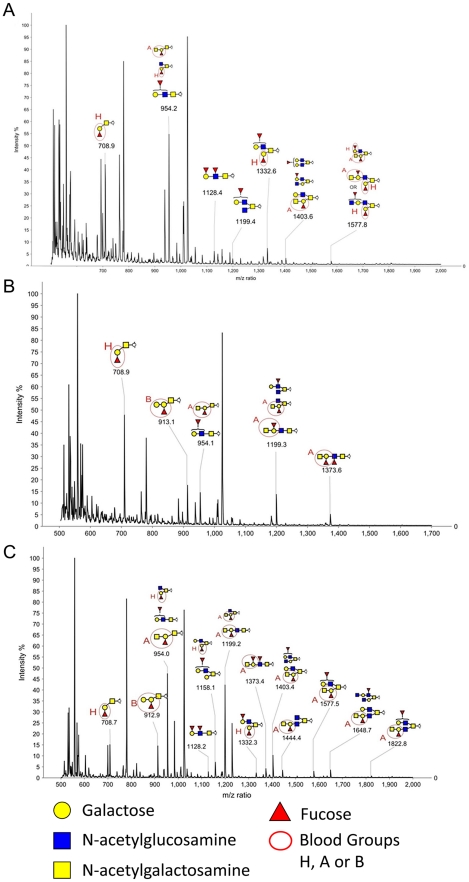
MALDI-TOF MS of O-glycans released from Rabbit Duodenum. Glycans were permethylated prior to MALDI-TOF analysis. Structures were assigned taking into account the molecular weight value and the fragment ions obtained upon MS/MS analysis. Non annotated structures correspond to non fucosylated O-glycans and permethylation adducts. **A**: Profile of fucosylated permethylated O-glycans released from rabbit duodenum (Sample 2). **B**: Profile of fucosylated permethylated O-glycans released from rabbit duodenum (Sample 4). **C**: Profile of fucosylated permethylated O-glycans released from rabbit duodenum (Sample 6).

The carbohydrates of the rabbit duodenum were further characterized through mass spectrometry (MS). Both N-linked and O-linked glycans were analyzed and blood group antigens have been mainly found on O-glycans, while N-glycans are largely terminated with galactose or sialic acid with virtually no fucose on their antennae (Fig S1). Many of the O-glycan's peaks identified through MALDI-TOF analysis (Table S4) have been shown to be a mixture of different structures after MS/MS analysis (Fig S2). In O-glycans, we observed a predominance of core 2 structures, followed by core 3 (Fig 1C). The smallest fucosylated glycan was observed at m/z 708, corresponding to a core 1 fucosylated trisaccharide bearing a blood group H epitope, while the largest fucosylated glycan had a composition of 8 residues arranged on a biantennary core 2 structure (m/z 1822) with one antenna carrying an A blood group epitope (Fig 6). Most of the samples analyzed showed high abundance peaks at m/z 708, and/or at mass 954, a trisaccharide with composition HexNAc2, Fuc, Hex, corresponding to different structures in different samples: after MS/MS sequencing, a mixture of core 1, 2 and 3 structures bearing blood group H, A and Lewis epitopes have been observed (Fig 6 and Fig S2). A peak at m/z 1199, with composition HexNAc3, Fuc, Hex, has also been observed in most of the samples analyzed, showing after MS/MS sequencing a core 3 structure carrying an H or Lewis X epitope in some of the rabbits, while in other rabbits it was recognized as a core 2, 3 and 4 with terminal A blood group on one antenna (Fig 6 and Fig S2). In 3 of the 10 samples analyzed, a peak at m/z 1373 was found, with composition HexNAc3, Fuc2, Hex, and after further analysis it was found to be a core 3 structure carrying a blood group A epitope (Fig S3). Detailed compositions of O-glycans of the 10 samples analyzed are reported in Table S3. In summary, we observed a high variety of O-glycan structures carrying blood group antigens H, A and B (Table S3). Blood group antigen B has been detected in samples 1, 4, 5, 6, 8, and 9 for a total of six B positive rabbits out of ten analyzed, in accordance with data from antibody binding analysis, while blood group antigen H has been detected in nine out of ten samples analyzed and only sample 9 did not show any glycan bearing H blood group epitopes. Mass spectrometry analysis could not distinguish between type 1 or type 2 based antigens but we observed poor reactivity on rabbit duodenal extracts of an anti-H type 1 specific antibody compared to the UEA-I reactivity, recognizing H type 2. On human saliva from O secretors, both reagents reacted equally well, suggesting that in rabbit duodenum, there is little, if any, type 1 based histo-blood group antigens (data not shown). In all of the 10 rabbits, both wild and domestic rabbits, detection of the B type 3 structure by mass spectrometry matched the B phenotype obtained with an anti-B antibody showing broad specificity toward all types of B antigens (Table 2). In contrast, detection of A histo-blood group structures by mass spectrometry and using a broadly reactive anti-A did not match. Thus, although three animals were unequivocally phenotyped as A-, the A type 3 structure was found in all 10 rabbit duodenum samples analyzed by mass spectrometry. A type 2 was only found in the rabbits that through antibody detection were determined to be A+, though two of the A+ phenotyped rabbits did not express A type 2. In order to determine if the discrepancy between the MS and the phenotyping results for the A type was not due to the specific anti-A that was used in the first place, the A- rabbits were then phenotyped again with several other broad binding, anti-A specific antibodies with confirmed recognition of the PAA-conjugated A trisaccharide. Yet, despite attempts to amplify the signal, the A- phenotyped rabbits remained A-, indicating that in some animals, although present, A type 3 epitopes are not detected on duodenum extracts by ELISA or immunohistochemistry, and the phenotypes of the 10 rabbit duodenum samples as determined by ELISA are noted in Table 2. Despite this discrepancy, it remains that both the A+ versus A− and B+ versus B− phenotypic dichotomies are as clear-cut as the A or B versus O distinction between humans.

**Table 2:** Detection of A and B with mass spectrometry and antibodies.

	A		B		
Rabbits	Mass spectrometry	Antibody	Mass spectrometry	Antibody	Phenotype
1	A type 3	+	B type 3	+	A+B+H−
2	A type 3	−	−	−	A−B−H+
3	A type 3	−	−	−	A−B−H+
4	A type 3; A type 1/2; A Lewis b/y	+	B type 3	+	A+B+H−
5	A type 3; A type 1/2; A Lewis b/y	+	B type 3	+	A+B+H−
6	A type 3; A type 1/2; A Lewis b/y	+	B type 3	+	A+B+H+
7	A type 3; A type 1/2	+	−	−	A+B−H−
8	A type 3	+	B type 3	+	A+B+H+
9	A type 3; A type 1/2	+	B type 3	+	A+B+H−
10	A type 3	−	−	−	A−B−H+

### Analysis of RHDV strains binding to rabbit tissues

To determine the sites of RHDV attachment to the duodenum, A+B+ rabbit duodenum sections were used and all six strains were found to bind to the duodenum surface but not the Brünner's gland, in accordance to A, B and H type 2 expression as discussed above. Binding was also detected on the surface epithelium of the trachea that expresses detectable amounts of H type 2 epitopes despite expression of A antigen, but not on the biliary ducts in the liver where B and H type 2 epitopes are not detectable in A+B+ animals (Fig 4).

**Figure 7:**
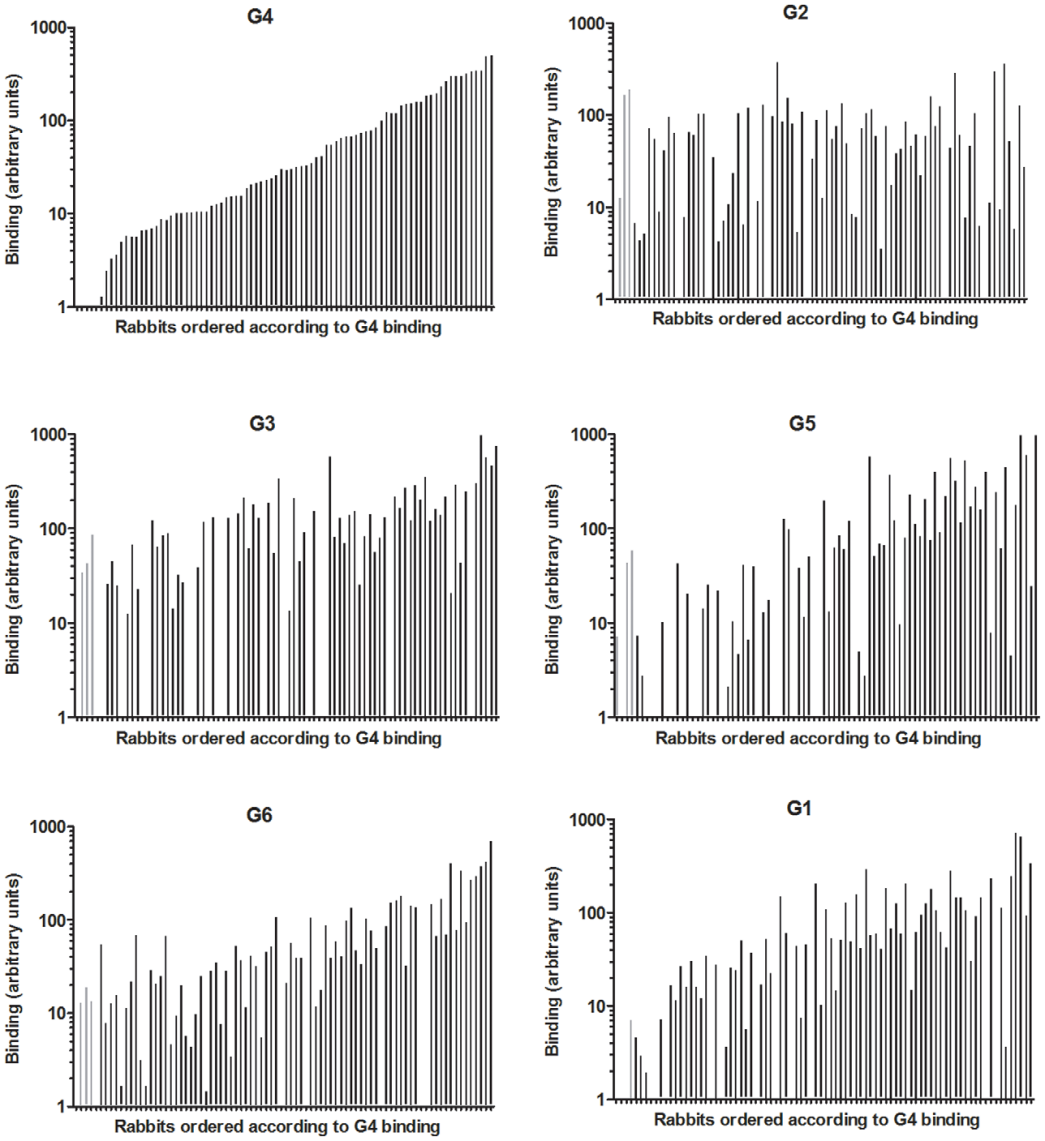
Binding of 6 RHDV strains to rabbit duodenum. Relative binding of G1–G6 to 84 individual wild and non-wild rabbit duodenums. Rabbits are ordered according to G4 binding in all graphs. The rabbits below the detection threshold of G4 and therefore in no particular order are displayed with gray bars.

The six strains of RHDV were then analyzed for binding to duodenum extracts using the same semi-quantitative system as for ABH phenotyping. Virus binding and ABH phenotypes were normalized, to account for variation of duodenum scraping, against *Concanavalin A* binding, a lectin which binds mannose of N-glycans. To analyze relationships between A, B or H expression and virus binding, animals were separated into three equal groups of weak, medium and strong binders and correlated to A, B and H presence or absence. G2 binding was significantly correlated to H type 2 expression. G3 binding did not correlate with the presence of either A, B or H antigen. G4, G5, G6 and G1 binding significantly correlated to A and B expression and inversely correlated to H type 2 expression, with the exception of G6 binding which did not correlate to B expression (Table 3, Table S5). It should be noted that most animals that express A also express B (A+B+ or A−B− phenotypes). Therefore in this association study, A and B antigens are linked. Nevertheless, a small subgroup of rabbits expresses A independently of B (A+B− phenotype) (Table S2). This subgroup was significantly associated with low G2 binding (p = 0.006) and inversely with strong G4, G5, G6 and G1 binding (p<0.05). In order to visualize differences in binding of these strains to individual animals, rabbits were ranked according to G4 binding in increasing order (Fig 7). Keeping the same order of relative binding for the five other strains clearly showed important individual differences with animals strongly recognized by some strains but poorly by others. Each strain showing a unique binding pattern, despite similar binding characteristics of G4, G5 and G6 as described above.

**Table 3:** Correlation between weak, medium and strong RHDV binding and A, B and H type 2 expression or no expression.

RHDV strains	A	B	H type 2
G2	NS	NS	*
G3	NS	NS	NS
G4	***	**	* †
G5	***	**	* †
G6	***	NS	* †
G1	**	**	* †

For each strain, animals were split into 3 equal groups according to the intensity of binding (weak, medium or strong) and association with A, B and H type 2 expression or lack of expression was tested by chi-square analysis. NS  =  not significant.

*p<0.05.

**p<0.005.

***p<0.0005.

**†:** correlation with low binding (inverse correlation).

### RHDV binding after enzymatic removal of ABH antigens

**Figure 8:**
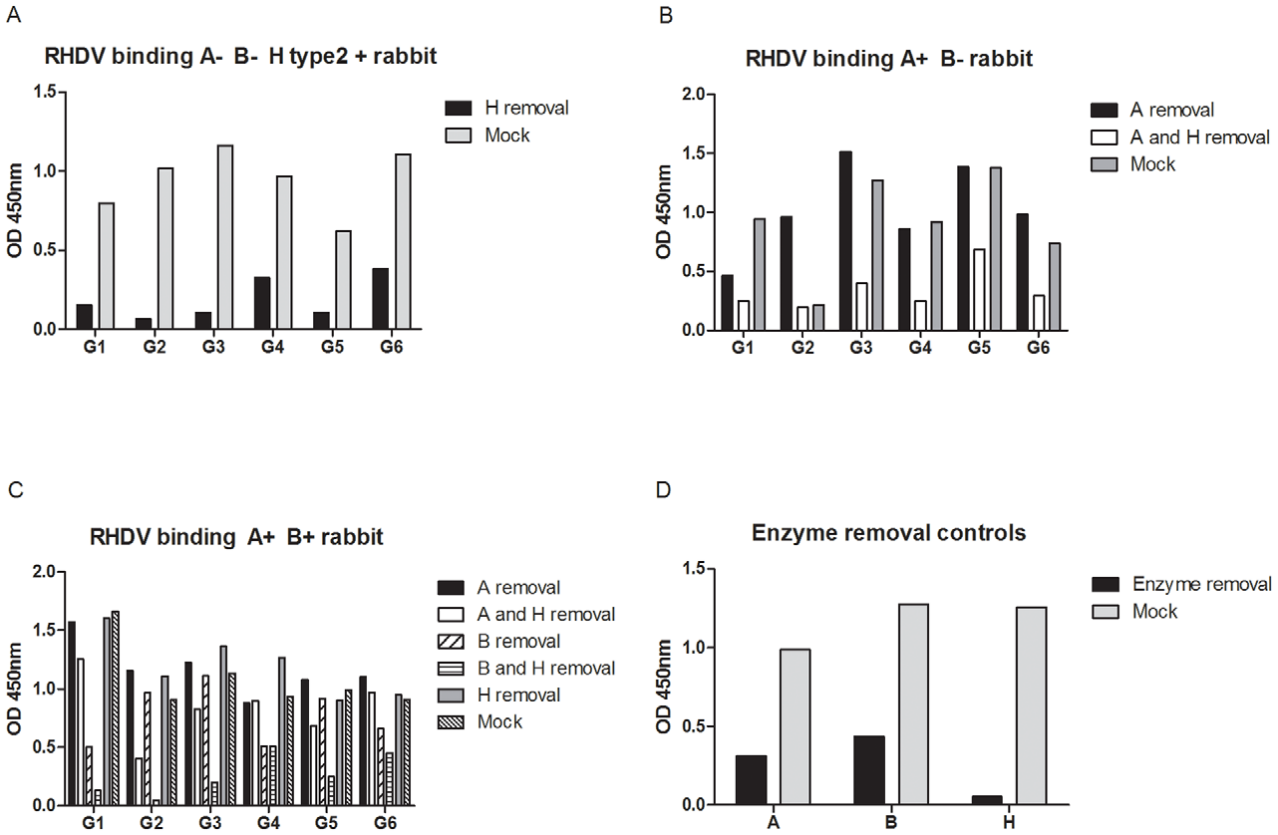
Enzymatic removal of A, B and H from three different rabbit duodenums. The H epitope was cleaved from an A−B− rabbit duodenum and G1–G6 binding was tested against H removal and a mock treated control (A). A and H were cleaved from an A+B− rabbit duodenum and RHDV binding was tested against A removal, A followed by H removal and a mock treated control (B). A, B and H were removed from an A+B+ rabbit duodenum where A removal, A and H removal, B removal, B and H removal and a mock treated control were tested for G1–G6 binding (C). Control of the enzymatic removal of A, B and H are shown (D).

To confirm that binding of each strain to rabbit duodenum extracts required the presence of A, B or H epitopes and to get a more complete picture of the strains specificities, A, B or H antigens of duodenal extracts were removed with specific glycosidases prior to virus binding assay. Extracts from three rabbits of three different phenotypes (A−B−, A+B+ and A+B−) were treated with either a galactosidase or an N-acetyl galactosaminidase, removing the galactose (Gal) of B or the N-acetylgalactosamine (GalNAc) of A respectively, to determine the role of A and B binding in the duodenum, followed by an α1-2 fucosidase to remove the fucose of H type 2 (Fig 8). Efficacy of each enzyme was determined by testing the binding of either an anti-A, an anti-B or UEA-I before and after treatment. The results shown in Fig 8D indicate that the fucosidase removed H type 2 almost completely, whereas treatments with either the N-acetyl galactosaminidase or the galactosidase only resulted in a partial removal of the A and B antigens, respectively. Removing the fucose of H type 2 confirmed that binding of all RHDV strains to A−B− rabbits was dependent on H (Fig 8A). In an A+B− rabbit, cleavage of the GalNAc sugar of A followed by removal of the fucose of H type 2 confirmed the significance of RHDV binding and the importance of A antigen expression (Fig 8B). Indeed, for G2 which was the only strain unable to recognize the A synthetic oligosaccharide and to show no relationship between RHDV binding and A expression, cleavage of the GalNAc residue of A allowed binding since it resulted in appearance of the underlying H type 2, which became accessible for binding. Inversely, the G1 strain preferred binding to A over H type 2, as seen in the decreased binding after removal of the A epitope, consistent with the results of agglutination. The other strains were not affected by the removal of A but showed a clear decrease of binding following removal of both A and H, indicating similar binding to A as to H type 2 on the duodenum extracts. Cleavage of the Gal and GalNAc followed by removal of H type 2 of an A+B+ rabbit indicated a major importance of B in the presence of A, as removal of the Gal of B followed by removal of the fucose of the underlying H type 2 abolished binding for G1, G2, G3 and G5 (Fig 8C). G4 and G6 binding also preferably bound B over A as removal of B decreased binding, whereas removal of A did not affect binding, or even slightly increased RHDV binding. However, neither G4 nor G6 binding was further decreased after removal of H as A was still expressed in the duodenal scrapings, allowing for binding of G4 and G6. Thus, the results of RHDV strain binding so far indicates binding of G2 to H type 2 and B, while the other strains are able to bind A, B and H type 2 with variable strength.

### Rabbit challenge with infectious RHDV

**Figure 9:**
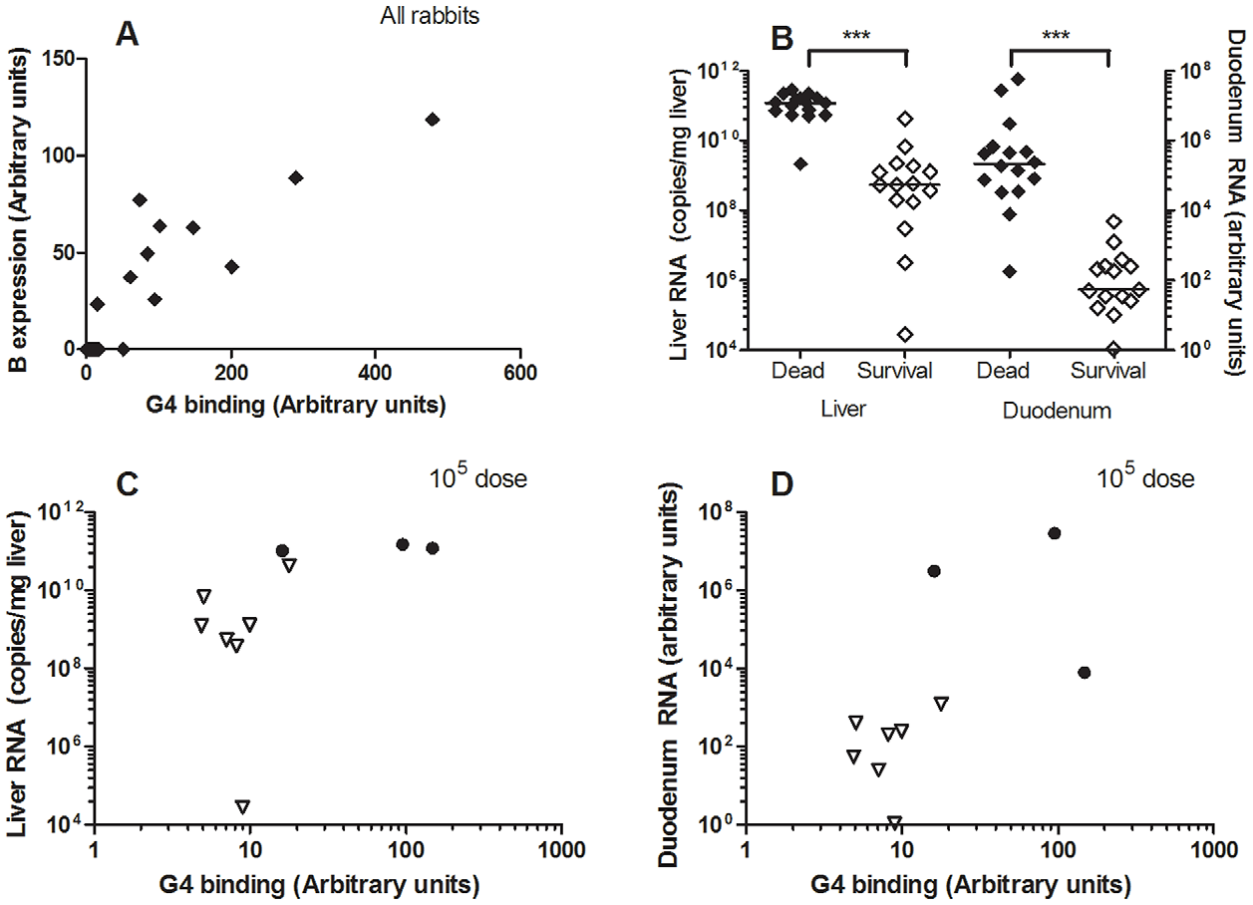
RHDV challenge of A+B+ and A−B− rabbits. 31 rabbits were infected with an A and B binding G4 strain at 10^5^, 10^7^ or 10^9^ virus copies. Rabbits either succumbed to infection or were sacrificed after 11 days. Duodenum and liver samples were collected at time of death. Duodenum was phenotyped for A, B and H expression and G4 binding. Duodenum and liver were also assayed for virus RNA via real time RT-PCR. (A) G4 binding plotted against B expression levels. (B) Viral load of liver and duodenum of all dead and surviving rabbits. Duodenum RNA is plotted as arbitrary units since the RNA was normalized against β actin to account for tissue preparation variations. Liver RNA is plotted as copy numbers since the tissue state due to infection did not allow for reliable quantification of rabbit RNA. (C–D) Virus RNA levels of liver or duodenum for 10^5^ infectious dose, where A+B+ (circle), A−B− (triangle), dead rabbits (black symbol) and surviving rabbits (white symbol).

Human norovirus binding to HBGA's has been determined to correlate with symptomatic infection in human volunteer studies and in outbreak studies. To determine the importance of carbohydrate binding regarding RHDV infection, domestic rabbits were challenged with the G4 strain (GenBank accession number AJ535094). This genetic group was chosen because it proved to be strongly dependent on binding to A and B antigens and because a breed of rabbits with a previously determined high A−B− frequency (63%) was available at the animal facility. Pre-challenge serum was collected from 6 of the animals, and no RHDV antibodies were detected. In addition, the rabbits from this animal facility proved negative during routine screening for the non-pathogenic calicivirus strain. Furthermore, any protection from cross-reacting antibodies can be excluded as non-pathogenic calicivirus strains circulating in France have been shown to provide no protection against RHD [12]. The 12 week old rabbits were then infected orally with 10^5^, 10^7^ or 10^9^ genome copies of a G4 liver extract. At necropsy of dead rabbits, typical RHD lesions were observed. Surviving animals were sacrificed 11 days after infection, however one rabbit within the highest infectious dose group was sacrificed 7 days post infection due to ethical considerations as it seemed severely ill. Post-mortem examination confirmed the presence of RHD lesions. Duodenum and liver samples were collected from all of the rabbits post-mortem. 7/10, 6/11 and 3/10 rabbits died from RHDV with an average survival time of 3.5 days, 3.5 days and 5 days in the groups infected with 10^9^, 10^7^ and 10^5^ genome equivalents, respectively (Fig S5). Post-mortem ABH duodenum phenotyping of the rabbits determined 3, 3 and 4 A+B+ rabbits in the 10^5^, 10^7^ and 10^9^ infectious dose groups, respectively, the remaining animals being A−B− (Table 4). Analysis of the ABH duodenum phenotype and G4 binding to the duodenum of all infected animals resulted in a B expression well correlated with virus binding of the rabbit duodenum of the infected animals (r^2^ = 0.78) (Fig 9A). Nevertheless, it should be noted that all animals were recognized by the G4 strain, albeit with great individual variation. Real time RT-PCR of RNA isolated from the duodenum and liver of the rabbits revealed viral replication in the liver of all animals (Fig 9B). The RNA levels in both liver and duodenum of rabbits succumbed to infection were significantly higher than viral RNA levels of rabbits sacrificed at 11 days post infection (Mann-Whitney, p<0.0001). It should be noted that this difference might be partly due to the difference in sampling time between dead and surviving rabbits (3–5 days vs 7–11 days). Within the rabbits infected with the lowest dose of 10^5^ genome copies all A+B+ rabbits (n = 3) died, while all A−B− rabbits (n = 7) survived the infection (p = 0.008, Fischer's exact test) (Fig 9C, D, Table 4). This was however not the case for the two higher-dose challenges (Fig S4, Table 4), indicating that A and B binding facilitates infection, though the lack of A and B antigens can be overcome by a high viral dose.

**Table 4:** A+B+ and A−B− phenotypes and survival and death of rabbits from G4 challenge experiment.

	A+B+		A−B−	
	Survived	Dead	Survived	Dead
10^5^ dose	0	3	7	0
10^7^ dose	2	1	3	5
10^9^ dose	1	3	2	4

### Analysis of ABH phenotypes in wild rabbit populations affected by RHDV

In order to study possible selection of ABH phenotypes after RHDV outbreaks, rabbit duodenums were collected from two populations located 15 km apart near Perpignan, southern France, where detailed information was available regarding RHDV. Rabbits were sampled by hunters from the two different populations, Claira and Canohès. The Claira population had never been affected by an RHDV outbreak. The Canohès population was heavily reduced during September 2006 by RHDV, where G5 was the primary RHDV- circulating in the area, though Iberian G1 strains were also detected during this period, and the population size strongly decreased (Stéphane Marchandeau, personal communication). Twenty two rabbit duodenums were collected in 2009 from the Claira population and only 5 from Canohès due to the low density of rabbits in the population. All of the 5 rabbits sampled from the Canohès population were B-, and all of them, either of the A+B− and A−B− subtypes, were significantly lower binders of G5, and similar binding results were seen with G1 (Table 5). In contrast, the Claira population where RHDV had never been detected had a high frequency of A+B+ (82%) and therefore only few B− animals (18%). Since the G5 strain binds preferentially to the B antigen, these results suggest that the B− phenotype could have been selected at Canohès following the devastating 2006 outbreak.

**Table 5:** Correlation of B expression and RHDV binding of rabbits from Claira, not RHDV infected, and Canohès, RHDV survivors, percentage in parenthesis.

	Phenotype^1^			RHDV binding^2^		
Populations	A+B+	A+B−	A−B−	Low	Medium	High
Claira	18 (82)	1 (5)	3 (14)	6 (27)	10 (45)	6 (27)
Canohès	0	2 (40)	3 (60)	5 (100)	0	0

1 p = 0.002, Fischer's exact test for B+ versus B− phenotype.

2 p = 0.019, Fischer's exact test.

In Australia, rabbits have been repeatedly infected with the G2 Czech strain of RHDV to control the rabbit population. Infecting a rabbit population with the same strain gives an interesting perspective to study selection from a single RHDV strain. Rabbits were sampled at three different locations, Hattah, Bendigo and Bacchus Marsh. Experimental challenges with RHDV have shown the Hattah and Bacchus Marsh populations to have developed partial resistance to infection (Brian Cooke, personal communication) and the non-pathogenic, partially protecting virus RCV-A1 has been detected in the Bendigo and Bacchus Marsh populations, but not in the Hattah population [15]. Rabbit duodenum extracts were analyzed for ABH phenotype and G2 strain binding. Similar to the above described French G2 strain, the Czech G2 strain bound Australian A+B− rabbits poorly (Table 6). The G2 Czech strain also showed binding to synthetic B and H, but not A, similar to the French G2 strain used above. In addition, both strains showed poor binding to the A+B− individuals regardless of the animals' origin (data not shown). Hattah was the population of the significantly highest frequency of A+B− rabbits and inversely with the lowest frequency of A−B− animals, which are most frequently strongly recognized by the G2 strains, suggesting selection for a subgroup of rabbits with potential of protection against infection with a G2 strain.

**Table 6:** Correlation of A and B phenotypes of rabbits from three populations in Australia with G2 strain binding (percentages in parenthesis).

	A and B expression^1^			RHDV binding^2^		
	Bendigo^3^	Bacchus Marsh^4^	Hattah^5^	Low	Medium	High
A+B+	13 (59)	8 (35)	11 (61)	9	13	10
A−B−	7 (32)	14 (61)	1 (6)	3	8	11
A+B−	2 (9)	1 (4)	6 (33)	9	0	0

1 p = 0.0016.

2 p = 0.00011.

3 No indication of genetic resistance to RHDV infection (Brian Cooke, personal communication) and protection through RCV-A1 antibodies.

4 Signs of genetic resistance to RHDV infection (Brian Cooke, personal communication) and protection through RCV-A1 antibodies.

5 Signs of genetic resistance to RHDV infection (Brian Cooke, personal communication) and no protection through RCV-A1 antibodies.

## Discussion

A recent study of the phylodynamics of RHDV indicated that France has been the most important source population for RHDV [20]. Although this may be due to sampling bias, a chronological relationship matching their phylogenetic positions has been established in France for the G2 to G5 strains [17]. The G1 strain used in the present study is a recent strain of Iberian origin and G6 strains showed no apparent chronological link with other strains. In France, G1 and G2, which includes the strain isolated in the first reported outbreak in China in 1984, have not been isolated since 1990, though G1 currently circulates almost exclusively on the Iberian Peninsula. In addition, since 2000 a few Iberian G1 strains have been identified in the South of France, along the Spanish border [19]. This may be the result of virus spread across the Pyrenean Mountains via insects or the wind [22]. The Iberian strains, suspected to originate from a single introduction of G1, have evolved separately from the other RHDV strains and cluster into 6 Iberian clades (IB1 to IB6) [22]. G3 has been isolated in France between 1990–1997 and G4 has been isolated from 1993–1999. G5, originally first detected in 1994 and G6 first detected in 1999 are both currently circulating in France. G6 corresponds to the first antigenic variant identified, RHDVa [18]. Although the neighbor-joining tree constructed using nucleotide sequences allowed the allocation of the strains tested into each of the six previously identified genetic groups, it is comparable to the topology presented by Kerr et al. [20]. That the G3, G4 and G5 genetic groups did not appear as clearly independent as previously reported [17] might be due to the use of complete nucleotide sequences of the capsid in the more recent studies [20] rather than just partial sequences used in the earlier study [17]. Alternatively, this can also be a result of the inclusion of strains that cover most of the worldwide genetic diversity. Nevertheless, the topology of the tree is highly supported by the bootstrap values which are all above 90% for the major nodes. Neighbour joining trees were constructed using amino acids sequences of the entire capsid or of the P2 subdomain with the aim to infer a correlation between the HBGA binding profiles and the evolution of RHDV. No such correlation was observed (data not shown). Regardless, the trees constructed using either nucleotide or amino acid sequences showed that that the six selected virus strains G1 to G6 represent a good cross section of the antigenic diversity amongst the known pathogenic forms of RHDV.

The gastrointestinal tract is protected by a thick layer of O-glycans constituting the glycocalix of epithelial cells or presented as soluble mucins. It is therefore not uncommon for pathogens of the gastrointestinal tract to interact with such carbohydrates to be able to access the underlying epithelial cell membrane. A major route of transmission of RHDV is the fecal-oral route and so far viral RNA of the closely related non-pathogenic viruses have been exclusively recovered from the small intestine [11], [12], [13], suggesting that these viruses are primarily enteric viruses but that the pathogenic strains do not remain confined in the gut [24], [25]. Since an RHDV strain was previously shown to bind to a carbohydrate structure expressed in the gastrointestinal and upper respiratory tracts of rabbits [21], in the present study we first aimed at determining if this characteristic was shared by other pathogenic strains belonging to different clusters of the RHDV phylogeny and this was analyzed by several methods. Analysis of the binding to a set of HBGA related synthetic neoglycoconjugates revealed distinct binding patterns between strains, although strong binding to the B type 2 motif was common to all strains. The strong H type 2 binding previously observed for a G2 strain was confirmed but the magnitude of H type 2 binding was quite variable among strains. All strains except G2 and G3 were able to bind the A epitope and binding to Le^y^ was observed only for G1 and G6. The uniquely strong binding of G1, a strain restricted to the Iberian Peninsula, to Le^y^ suggests an important role of the host genetic background. G1 is the only genetic group present in that region, a fact that might be correlated with the Pyrenees acting as a barrier to the dispersal of both the virus and rabbits, but also by the fact that the European rabbit originated in that area and that populations of other parts of the world consist of only a subset of that original gene pool [26], [27], [28], . The higher genetic diversity of the Iberian rabbits may be reflected in the need of the virus to explore other carbohydrates for binding.

Binding to synthetic sugars provides information on the specificity for isolated carbohydrate motifs but the presentation and density of the sugar on the scaffold may not mimic expression in the tissue or on cell surfaces. In order to confirm that all RHDV strains bind to HBGAs, albeit with some differences in specificity, binding to human red blood cells (RBC) that express type 2 based HBGAs (H type 2 on O RBC, A type 2 on A RBC and B type 2 on B RBC) was examined through agglutination. All strains tested agglutinated human erythrocytes. They showed distinct blood group specificities, although here also a common feature was B blood group recognition. The ability of RHDV to agglutinate human RBCs has been observed soon after the virus discovery [2], although the existence of non-agglutinating strains was later reported [23], [31]. As the six strains that we tested spanned the RHDV phylogenetic diversity, our results suggest that all RHDV strains could be agglutinating. There was no indication of the RBCs blood groups used in the reports of non-agglutinating strains [23]. The use of O or A RBCs could thus well explain why some strains appeared non-agglutinating. Regardless, our results show that the ability of RHDV to recognize HBGAs has been maintained throughout RHDV evolution but with somewhat distinct strains specificities. We therefore investigated whether rabbits showed HBGA diversity.

HBGA expression in rabbits has previously been reported through analysis of glycolipids of the small intestine or by immunohistochemistry in the small and large intestines, respectively [21], [32], [33], [34]. However, the diversity of expression in the small intestine of individual animals has not been studied previously. We observed here that rabbits are able to express either A, B or H (Le^y^), mainly based on type 2 and type 3 precursors. With immunohistochemistry, nine wild rabbits tested were found to be either A+B+ or A−B−. A and B were expressed on the duodenum surface mucosa, whereas the underlying Brünners' glands were negative. Expression of A appeared stronger than B expression, and in tissues as trachea and bilary ducts where A and H type 2 expression was weak, B was negative, which was further confirmed with the semi-quantitative phenotype assay. In both assays, the concentration of anti-B necessary to detect the presence of B epitopes was at least 10 times higher than that required to detect B antigen either on human epithelial tissues or saliva. In contrast optimal dilutions of anti-A or UEA-I were similar to detect A or H epitopes in both species. This clearly indicated that in rabbits, B antigen was present in smaller amounts than A and H, or that it was less accessible. Mass spectrometry analysis of the duodenum determined that HBGA motifs were mainly present on O-linked rather than on N-linked glycans and revealed extensive individual variation. This great individual variation was also found through a semi-quantitative analysis of A, B or H expression with specific reagents. Strikingly however, no rabbits, out of over 200 screened individual duodenums, were found to completely lack expression of A, B or H, indicating that a clear-cut non-secretor phenotype does not exist in rabbits, unlike in humans. These data are in line with the findings of Guillon et al. [35] who observed that despite a large polymorphism of the *Fut2* and *Sec1* coding sequences of wild rabbits, involved in synthesis of H type 2 and H type 3, all of the detected Fut2 enzyme variants were functional, indicating that the genetic diversity of H expression in rabbits is controlled by the level of expression of the α1,2fucosyltransferases or by other as yet unknown mechanisms, but not due to null alleles of an α1,2fucosyltransferase gene like in humans. Antibody binding data and mass spectrometry were in accordance for detection of B antigen amongst the 10 rabbits analyzed with mass spectrometry, revealing B+ and B- rabbits. As for detection of A antigen, there was divergence between the mass spectrometry data and the antibody binding. Three rabbits with detectable A epitopes in mass spectrometry appeared A negative using antibodies, despite the use of 4 different well-characterized antibodies able to bind all types of A. For reasons still unclear, all A epitopes are not accessible to antibody binding. A possible explanation is that the A antigen of these three rabbits is exclusively of type 3, i.e. very short O-glycans where larger surrounding glycans may sterically hinder binding of the antibodies. A second complication may be that glycans may be present not only on proteins but also on glycolipids. Earlier work on glycolipids from rabbit small intestine showed expression of A type 2 and B type 2 in the small intestine of rabbits [33], [34]. A differential expression of A on O-glycans and glycolipids along with difficult detection of the short A type 3 may account for the difference between mass spectrometry analysis and antibody assay.

A complete understanding of the ABH polymorphism in rabbits will require a full genetic description of the system, which appears quite different from that in humans. Thus the frequencies of the different A and B phenotypes (A+B+, A+B− and A−B−) in the various populations that we studied cannot be explained by the polymorphism of a single *ABO* gene as in humans and our preliminary genetic analysis indicates that there are at least 6 *Abo* genes in rabbits located in tandem in the genome. A similar situation has already been described in rats which have been reported to have a variable number of *Abo* genes (up to 5), with some genes encoding A enzymes and others encoding B enzymes [36], [37]. Therefore, despite a generally conserved expression of ABH antigens across mammalian species, the genetic mechanisms leading to diversity of expression and intraspecies polymorphism are quite variable. Here the major observation regarding rabbits is that they present extensive individual variability of A, B and H expression in the duodenum which is a primary site of attachment for RHDV leading us to analyze the relationships between ABH expression and the binding properties of different strains.

**Figure 10:**
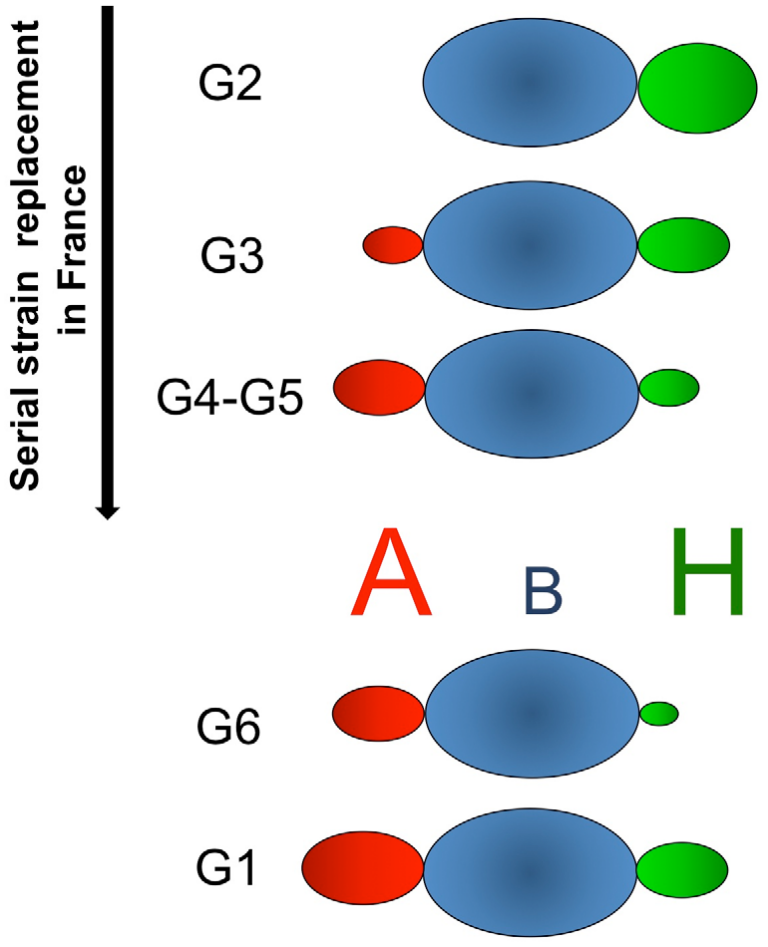
Summary of the RHDV strains specificities for ABH antigens. The size of the A, B and H letters represents the level of expression in the rabbit duodenum and the volume and color intensities of the ovals correspond to the ability of each strain to recognize the epitopes. The strains shown above the ABH antigens (G2–G5) serially replaced each other in France (G2: 1988–1990; G3: 1990–1997; G4: 1993–1999; G5: 1994–2009), whereas those shown below evolved independently.

As rabbit duodenums express complex patterns of HBGAs, statistical calculations were used to reveal relationships between individual RHDV strains binding and HBGA expression. Strong relationships were established with A+B+, A+B− and A−B− in a strain-specific manner, though the relative importance of each epitope was not completely clear. Enzymatic removal of each A, B or H epitope allowed a better assessment of the role of each of these ligands. The results showed that the strains are neither A, B nor H specific but more or less dependent on the level of expression of each of these antigens. Nevertheless and most importantly, for each RHDV strain, binding to individual duodenum extracts ranged from very weak to very strong, with the various strains clearly showing differential recognition of individual animals. Considering the results of the binding assay to synthetic sugars, of human RBCs agglutination, of the association with rabbits ABH phenotypes and of the enzymatic removal of ligands, G2 binds to B antigen as well as to H type 2 but is not able to recognize A. As a result, due to the masking of H type 2 by A, G2 only poorly binds to A+B− animals. Interestingly, strains which later displaced G2 in France, G3–G6, as well as G1 are all able to bind A. G3 was able to bind A, B and H type 2, which explains why no significant association was found between duodenum binding and rabbit ABH phenotypes. G4, G5, G6 and G1 preferentially recognized A+B+ or A+B− animals over A−B− animals. For these strains, binding to duodenum samples was more strongly associated with the presence of A antigen than of B antigen and inversely associated with the presence of H antigen. However, when A and B were both present, enzymatic removal indicated a greater importance of B over A despite an apparent higher expression of A antigen compared to B antigen in the duodenum. This apparent discrepancy can be explained by their stronger binding to B antigen than to A antigen along with the fact that B antigen is always present when A antigen is also expressed whereas A antigen can be expressed in absence of B in A+B− animals. The ability to bind the A+B− animals therefore explains the stronger association with A than with B expression. Nevertheless, despite similar binding patterns between G4, G5, G6 and G1, these strains showed slight differences resulting in different recognition patterns for individual rabbits. A schematic diagram summarizing these observations is presented in Fig 10.

Although HBGA binding has been determined as crucial for symptomatic infection by norovirus in humans (see below), the role of HBGA binding has not been directly established for RHDV infection of rabbits. For this purpose G4, a strain with a marked B-specific and to a lesser extent A-specific binding, was used in a challenge experiment in combination with a rabbit breed of domestic rabbits with high A−B− frequency. When rabbits were challenged with 10^5^ genome copies there was a clear relationship between survival and the A−B− phenotype (or low virus binding) but the relationship vanished at higher virus challenge doses. It should be noted that duodenum extracts of all animals were recognized by the G4 strain, although binding to some individuals was weak. Thus, infection with a high viral dose can compensate for weak viral binding to HBGAs. The increased survival rate at low infectious doses was not achieved by avoiding infection. Regardless of their survival, all rabbits in the experimental challenge study had detectable levels of RHDV-RNA in the duodenum and liver, indicating that all rabbits became infected. In addition, the groups infected with high doses of virus (10^9^ and 10^7^) still showed untypically high survival rates (30% and 54%, respectively), and lower virus loads in the tissues of the survivors. These findings suggest additional mechanisms of controlling the RHDV infection, leading to a less severe course of the disease and overall lower virus titers. In addition, G4, a strain no longer detected in France, may have been less virulent than other circulating strains. Noteworthy, hepatocytes are prone to potent viral propagation despite being completely devoid of HBGA [38]. Thus, our results suggest that HBGAs function as an attachment factor rather than the main cellular receptor.

Previous studies show that the amount of virus in fly spots from flies feeding on RHDV infected livers were sufficient to infect rabbits [39]. Similarly, it was shown that as few as 1 to 10 viral particles may be sufficient for infection with human noroviruses [40]. Thus, it is conceivable that 10^7^ or 10^9^ genome copies correspond to very high amounts of viral infectious particles, much higher than the doses involved in natural transmission during RHDV outbreaks. It is therefore likely that mechanisms allowing protection at low infectious doses will have noticeable effects on rabbit survival rates during natural outbreaks.

In order to investigate if selection pressure from RHDV leads to increased frequencies of low binding HBGA phenotypes in natural populations, wild rabbits from several locations in France and Australia were investigated. As rabbit populations are closely monitored in France, this allows for studying populations with a known RHDV-infection history that may have been under selection pressure for weak binding HBGA phenotypes. We had access to rabbits from two different French wild rabbit populations located 15 km apart. The first site, Claira, carries a high density population of rabbits in an area otherwise heavily hit by RHDV outbreaks, and has no evidence of previous RHDV infection. The second site, Canohès, has a population recovering from a major RHD outbreak due to a G5 (or possibly Iberian G1) strain in September 2006 that left few survivors, and small additional mortalities were again recorded in 2007 and 2008. Despite the limited sample size from the recovering population, we observed a highly significant weaker G5 and G1 binding associated with B- phenotypes among the descendants of RHDV survivor rabbits when compared to the neighboring control population, suggesting that the virus contributed to select animals with a weak binding HBGA phenotype.

In Australia, RHDV has been purposely introduced in 1996 to limit the damage caused by rabbits. RHDV quickly proved to be efficient at drastically limiting the population size [41], however in recent years rabbit survival has again increased due to several factors, such as development of immunity in the populations [42], [43], facilitated by partially cross-protecting antibodies from infection by non-pathogenic caliciviruses [15]. In addition, the appearance of genetic resistance to RHDV has recently been observed at some locations where the virus had previously proven very efficient at eliminating rabbits (Brian Cooke, personal communication). Rabbits were sampled from three different populations in Victoria, Hattah Kulkyne National Park, a site with indication of genetic resistance to RHDV and no protection from the non-pathogenic RCV-A1, Bacchus Marsh, a population with resistance to RHDV and protection from circulating RCV-A1 and Bendigo with little resistance to RHDV and with circulation of RCV-A1(Tanja Strive, unpublished results) [14]. We found the A+B− frequency to be significantly increased in the Hattah population. This phenotype is correlated with low virus binding of both the French G2 strain as well as the G2 Czech, the only strain currently circulating in Australia. The presence of a partially protective benign calicivirus in the two other populations likely resulted in increased survival rates during initial RHDV outbreaks and therefore likely promoted the establishment of immunity in the populations which protected them against subsequent outbreaks. It is feasible that this reduces the selective pressure towards weaker binding HBGA phenotypes as a means to avoid lethal RHDV infection. At present we cannot formally exclude that the observed associations between low HBGA binding and survival could be due to genetic drift rather than from selection by the virus. Nevertheless, it is quite remarkable that the observed associations occurred in a strain-specific pattern, where in the French population affected by a G5 strain with largely B-dependant binding a large increase in B- rabbit frequency occurred. On the other hand, the G2 strain steadily introduced in Australia is able to bind B as well as H type 2 with no ability to bind A, therefore poorly recognizing rabbits with no B expression and where A masks the otherwise available H type 2 (A+B−). Thus, the observed associations between survival or genetic resistance and HBGA binding matched the strain carbohydrate specificity. In addition, a prior study by Guillon et al. showed an association between survival to another devastating RHDV outbreak in France and a haplotype at the α1,2fucosyltransferases locus [35]. Although in the latter case the binding of the responsible RHDV strain could not be analyzed, collectively these analyses of wild animals indicate that RHDV outbreaks may select animals depending on their HBGA binding characteristics in a strain-dependent manner. Characterization of the binding profile of two G2 strains showed that they did not bind to the A antigen, unlike the five other RHDV strains tested. Since G2 strains appear to be the first pathogenic strains that circulated when emergence of RHDV was observed, acquisition of A antigen recognition by subsequent strains could have allowed a better coverage of the ABH diversity and targeting of the less susceptible and positively selected A+B− animals. Collectively, the data suggest that HBGA diversity tends to restrict the virus transmission and therefore to protect the host population, which is in accordance with the results of the models developed by Fouchet et al. [44].

The initial observation that RHDV binds to HBGAs led to the discovery that human noroviruses of the *Norovirus* genus (NoV), which cause gastrointestinal infection commonly known as “winter-vomiting disease”, also bind HBGAs [45], [46], [47], [48]. Most human norovirus strains bind α1,2-fucosylated HBGA structures such as H type 1, Lewis b, Lewis y, A or B antigens (Fig 1), which are expressed on epithelial cells and mucins of the gastrointestinal tract and saliva of individuals of the secretor phenotype [49]. Secretor individuals possess a functional *FUT2* gene (Fig 1A), encoding an α1,2 fucosyltransferase expressed on many epithelial cell types. Inactivating mutations of the *FUT2* gene results in a non-secretor phenotype where none of the above mentioned structures are expressed on most epithelial cells [50]. Human noroviruses cause a relatively mild, transient infection in healthy individuals although they can be responsible for more severe gastroenteritis in immunocompromised individuals, the elderly and in young children, particularly in developing countries [51]. In human volunteer studies, strain-specific binding of HBGAs correlated to infection and norovirus antibody detection [52], [53]. Thus, nonsecretor individuals proved completely resistant to infection by the Norwalk strain that binds to H type 1, Lewis b and the A antigen. Moreover B-type individuals who are poorly recognized by the strain were more likely to be either non-infected or to remain asymptomatic. Likewise, analysis of outbreaks showed a strong impact of either the secretor or the ABO phenotypes on infection [54], [55], [56], [57], [58]. Analyses of the carbohydrate specificity of many strains showed great variation depending on the combined *ABO* and *FUT2* polymorphisms. Thus, each strain recognizes a subset of the population only, although collectively NoVs can recognize all individuals except the very small subgroup of nonsecretors/Lewis negative (FUT2-/FUT3-) individuals who lack both the α2-linked fucose added by the FUT2 enzyme and the α4-linked fucose added by the FUT3 enzyme [45], [59], [60]. These data indicate that HBGAs act as attachment factors required for infection by noroviruses but also that their polymorphism contributes to restriction of the transmission of any given strain. Such a relationship between the host and the pathogen diversity suggests co-evolutionary processes or an adaptation of the virus to pre-existing host diversity. Histo-blood group antigens polymorphism is maintained in human populations for reasons that are still unclear. Nevertheless, it has been shown that the *ABO* and *FUT2* genes are under balancing frequency-dependent selection [61], [62], [63], [64], [65]. In addition, the *FUT2* gene has been subjected to intense gene conversion with its paralogues *FUT1* and *Sec1*during mammalian evolution [66]. In various species, including humans, many primate species, rat, mouse, and pig *Sec1* is a pseudogene. Yet, some of the alleles encode weakly functional enzymes in rabbits [35]. The *ABO* gene family underwent a birth-and-death mode [67] of evolution during vertebrate evolution [4]. These elements strongly suggest that through their polymorphism HBGAs are involved in interactions with environmental factors, most likely pathogens and the present observations strongly support of this view.

In conclusion, our work showed that similar to human noroviruses and despite a lower genetic diversity, RHDV strains bind to carbohydrates of the HBGA family with distinct specificities allowing them to preferentially recognize some subgroups of the host population. Due to variable cross-recognition of the available carbohydrate epitopes, strain binding patterns do not fit precisely with the ABH phenotypes of individual animals, generating complex patterns of recognition within populations. These carbohydrates act as attachment factors that facilitate infection, or at least symptomatic expression of the disease. Survivors to outbreaks are selected among animals showing the lowest binding and most protected from lethal disease. The polymorphism of ABH expression would thus act to generate genetic resistance to RHDV at the population level.

## Material and Methods

### Ethic statement

Work involving the acquisition and sampling of French wild rabbits was carried out in strict accordance with the bylaw (Arrêté N° 2009-014) issued by the Paris Prefecture. The acquisition and sampling of Australian wild rabbits was carried out in strict accordance with the guidelines of the Australian Code of Practice for the Care and Use of Animals for Scientific Purposes. All procedures were approved by the Commonwealth Scientific and Industrial Research Organisation (CSIRO) Sustainable Ecosystems Animal Ethics Committee (Licence # SEAEC 06-31). Rabbits were caught alive in cage traps or with ferrets. The cages were set on or around active rabbit warrens and baited with diced carrots. When ferrets were used, warren entrances were covered with purse nets and a ferret was released into the warren to flush out the rabbits. Caught animals were killed by cervical dislocation. The challenge study performed on laboratory animals was carried out in strict accordance with the recommendations of the French National Guide for the Ethics of Animal Experiments. The protocol was approved (Permit Number: 10/03/09-05B) by the ethics committee “ComEth Anses/ENVA/UPEC” registered under number 16 to the National Committee of Ethics on Animal Experiments of the Ministry of Higher Education and Research. All efforts were made to minimize suffering (observation of animals twice a day, euthanasia of animals showing signs of suffering) and euthanasia was performed under xylacine and ketamine anesthesia.

### Rabbit duodenum sampling

Rabbit duodenums were collected from either domestic New Zealand White rabbits, from 22 wild rabbits of a dense population in Claira, France and from 15 wild rabbits from small populations, including Canohès, with previously documented RHDV infection in the area of Perpignan, France, not far from the Spanish border and from 63 animals from 3 populations in Australia. The first 5 cm posterior to the gastroduodenal junction was removed after clearing the section from intestinal contents, the sample was vigorously rinsed in phosphate buffered saline (PBS) and stored in RNAlater (Sigma-Aldrich, St. Louis, MO). Sections of the duodenum were then rinsed in PBS, opened and scraped into RTL lysis buffer (Qiagen, Hilden, Germany) containing β-mercaptoethanol. The tissue scrapings were homogenized and split into three parts for ELISA assays, RNA and DNA extraction. The ELISA scrapings were boiled for 10 minutes.

### Duodenum phenotyping ELISA

Rabbit duodenum scrapings were phenotyped using ELISA. Briefly, the duodenum scrapings were diluted in duplicates in eleven two-fold dilutions with final dilutions ranging from 1/100 to 1/102,400 in 0.1 M sodium carbonate buffer pH 9.5 on a maxisorb plate (ThermoFischer scientific, Waltham, MA). Antibody dependent assays were blocked with 5% non-fat dry milk (Régilait, Saint-Martin-Belle-Roche, France) diluted in PBS while lectin assays were blocked with synblock (AbD serotec, Oxford UK). A antigen was detected using mouse monoclonal anti-A antibodies, 2A12, 2A21, and 2A15, all previously characterized to specifically bind A antigen based on all types of precursor structures [68]. B antigen was detected using a specific mouse monoclonal B49, a B-specific broadly reacting antibody [69]. H type 2 (Le^y^) expression was determined using HRP conjugated *Ulex europaeus*-I (Sigma-Aldrich, St. Louis, MO). Secondary horse radish peroxidase (HRP) conjugated anti-mouse (Uptima/Interchim, Montlucon FR) was used for A antigen detection and a biotin conjugated anti-mouse (vector laboratories, Burlingame, CA) followed by HRP conjugated avidin (vector laboratories, Burlingame, CA) for B detection due to relatively low amounts of B antigen in rabbit tissues. TMB (BD Bioscience, San Jose CA) was used as a substrate for all assays and O.D. values were measured at 450 nm.

### RHDV preparation

RHDV was prepared from infected rabbit livers. Livers were cut into small pieces and homogenized with PBS at 0.25 g/mL. Liver/PBS mixtures were centrifuged 20 minutes to remove cellular debris. RNA was isolated from 200 µL of each liver preparation with the RNeasy mini kit (Qiagen, Hilden, Germany) according to manufacturer's instructions and 5 µL of isolated RNA was reverse transcribed from each liver. RNA was reverse transcribed with random primers and Superscript II reverse transcriptase (Invitrogen, Carlsbad CA) according to instructions for first-strand cDNA synthesis. 2 µL cDNA/well was further analyzed in real-time PCR with primers and probes designed according to Taqman chemistry, forward primer: 5′ TCTGTCGTCAGGCGCACC 3′, reverse primer: 5′ GACGAGTAGTTGTTGAGCGAAAG 3′ and probe: 5′ FAM-CAGTACGGCACAGGCTCCCAACCA-TAMRA 3′. A plasmid containing the amplicon was used as a standard of 4 different concentrations for each run on an Mx3005P (Agilent technologies, Santa Clara CA). All real-time PCR tests were run in triplicates and together with several previously quantified RHDV strain cDNA. In all further assays RHDV from each strain was used in similar concentrations.

### RHDV binding assay

RHDV binding to rabbit duodenum scrapings was analyzed in the same manner as the duodenum phenotype ELISA. Here rabbit duodenum scrapings were diluted in a range of 11 dilutions and coated in 0.1 M sodium carbonate buffer. Plates were blocked with 5% non-fat dry milk diluted in PBS or distillated water. RHDV from six strains belonging to genetic groups G1 to G6 (Fig 2) was prepared from infected livers as described above. High titered rabbit sera Lp4 or an RHDV monoclonal antibody 2G3 was used for G1–G5 and G6 detection, respectively. Secondary antibodies anti-rabbit conjugated with HRP were used against Lp4 and anti-mouse biotin followed by avidin-HRP was used for RHDV detection with 2G3 anti-RHDVa (G6) monoclonal antibody [70]. Addition of substrate and measurement of the plates were performed as described above.

RHDV binding to synthetic sugars was tested by screening a panel of PAA-conjugated and BSA-conjugated sugars (Table S1). 1 µg of synthetic sugars were coated. For the first screening a high concentration of RHDV was used. For the detailed analysis of binding to the positive synthetic sugars a range of dilutions was used. RHDV binding ELISA was performed as described above. RHDV binding step was performed at 4°C for PAA-conjugated sugars and at 37°C for BSA-conjugated sugars, though no major difference was visible at the alternate temperatures.

### Statistical calculations for ABH phenotyping and RHDV binding

A threshold was set at 3 times the background for each phenotype or RHDV binding assay and dilution value of each sample for crossing the threshold were analyzed. All values were normalized against values obtained with the mannose-binding lectin *Concavalin A* to control for differences in the amount of material scraped, as protein quantification proved to be difficult due to the addition of β-mercaptoethanol to remove any potential anti-RHDV antibodies in the duodenum that would interfere with the analysis. Different sets of rabbits regarding ABH phenotypes and RHDV binding were analyzed with a chi-squared test or Fischer's exact test.

### RHDV agglutination

Blood from A, B and O individuals was washed 3 times in PBS and diluted to 2% RBC. RBC were added to virus dilutions in V-shaped wells, 1×10^9^ genomic copies of virus was used as determined through real time RT-PCR, described above. Agglutination titers were determined after 3 h incubation at room temperature.

### Immunohistochemistry

Tissue sections of nine French wild-rabbit duodenums, liver and trachea were de-parafinated through baths in LMR and ethanol. Endogenous peroxidase activity was blocked with 0.3% hydrogen peroxide. Non-specific binding was blocked with 5% goat serum in PBS. HRP conjugated *Ulex europaeus*-I (Sigma-Aldrich, St. Louis, MO) at 0.8 µg/mL, anti A monoclonal antibody 2A21 and anti B monoclonal antibody B49 were used for binding to H type 2, A and B phenotyping respectively. A rabbit expressing both A and B antigens was used for RHDV binding. All 6 strains described above were used at 2×10^9^ genome copies/ml (see RHDV preparation) and detected with the mouse monoclonal anti-RHDV antibody 2G3 [70]. Dilutions of lectin, antibodies and virus were done in 1% BSA in PBS and binding at 4°C overnight. A biotinylated anti-mouse antibody (vector laboratories, Burlingame, CA) diluted in 1% BSA in PBS was bound to all of the assays with primary mouse antibodies. Binding of the biotinylated anti-mouse antibody was followed up with HRP-conjugated avidin vector laboratories, Burlingame, CA) also this diluted in 1% BSA in PBS. Substrate was added to the slides (AEC kit, vector laboratories, Burlingame, CA) followed by Mayer's hemalum solution (Merck, Whitehouse Station, NJ) for contrast staining.

### Release of glycans and mass spectrometry analysis

#### Tissue homogenization and protein extraction

10 samples of rabbit duodenum were analyzed through a procedure previously described [71]. Briefly, tissues were first weighed and then were disrupted on ice by using a homogenizer in the presence of 1 mL degassed homogenization buffer (0.6 M Tris, 150 mM NaCl, 5 mM EDTA and 1% CHAPS at pH 8.5). Lysates were then dialyzed against 50 mM ammonium bicarbonate (Ambic; Sigma-Aldrich, UK) buffer, pH 8.5, at 4°C for 48 h with several buffer changes. This was followed by lyophilization of the tissue lysates.

#### Protein reduction and carboxymethylation

Proteins were reduced and carboxymethylated as described previously [72]. Briefly, lyophilized tissue lysates were reduced at 37°C for 1 h in 50 mM Tris-HCl buffer, pH 8.5, containing 10 mg/ml dithiothreitol (Roche, UK). Carboxymethylation was carried out by the addition of 0.6 M Tris (Sigma-Aldrich, UK) buffer containing 12 mg/mL of iodoacetic acid (Sigma-Aldrich, UK) at room temperature in the dark for 1.5 h. After carboxymethylation, samples were dialyzed against 50 mM ammonium bicarbonate, pH 8.5, at 4°C for 48 h with several buffer changes, and subsequently lyophilized.

#### Cleavage into peptides and glycopeptides

Protein digestion using TPCK-treated, bovine pancreas trypsin (EC 3.4.21.4, Sigma-Aldrich, UK) and purification was performed as described previously [73]. For each microgram of tissue sample, 1.2 µg of trypsin from a trypsin solution in 50 mM ambic buffer (pH 8.4) was added to the lysates and was incubated at 37°C for 14 h, followed by purification with Oasis HLB extraction cartridges (Waters, UK). The cartridges were successively conditioned with 5 mL of methanol, 5 mL of 5% (v/v) acetic acid, 5 mL of propan-1-ol and 15 mL of 5% (v/v) acetic acid. The digested samples were then loaded onto the cartridges, washed with 20 mL of acetic acid (5% v/v) to remove hydrophilic contaminants, and then eluted sequentially with 5 mL of each 20% and 40% propan-1-ol solution in 5% (v/v) acetic acid. The propan-1-ol elutions, which contain glycopeptides and peptides, were concentrated with the Savant SpeedVac (Thermo Fisher Scientific, UK), combined and subsequently lyophilized overnight.

#### Digestion of N-glycans from glycopeptides

The lyophilized glycopeptide and peptide fractions were dissolved in 200 µL of 50 mM ambic buffer (pH 8.4) before adding in a total of 5 U of PNGase F (EC 3.5.1.52, Roche) and incubate at 37°C for 20–24 h. The samples were then lyophilized overnight and re-dissolved in 200 µL of 5% (v/v) acetic acid for solid-phase extraction with Sep-Pak C_18_ cartridges (Waters, UK) as described for the tryptic digested lysates. The acetic acid fractions, which contained the released N*-*glycans, were lyophilized and later permethylated. The propan-1-ol eluents, which contain O*-*glycopeptides, were combined prior to lyophilisation.

#### Release of O-glycans from glycopeptides

Reductive elimination of O*-*glycans was performed as explained previously [73]. Four hundred microliters of 0.1 M potassium hydroxide (Sigma-Aldrich, UK) containing potassium borohydride (54 mg/mL) (Sigma-Aldrich, UK) was added to dried samples and incubated at 45°C for 14–16 h. The reaction was terminated by adding a few drops of 5% (v/v) acetic acid followed by purification with Dowex 1-X8 desalting column (Sigma-Aldrich, UK). The columns were first washed with 15 mL of 5% (v/v) acetic acid. Next, the samples were loaded and eluted with 5 mL of 5% (v/v) acetic acid. The volume of the eluents was reduced with Savant SpeedVac followed by overnight lyophilization. Excess borates in the samples were removed by co-evaporating with 10% (v/v) acetic acid in methanol under a stream of nitrogen at room temperature. The purified native O*-*glycans were subsequently permethylated.

#### Chemical derivatization of N and O-glycans

Sodium hydroxide (NaOH) permethylation was performed according to an established procedure as described previously [74]. Briefly, 5–7 NaOH pellets (Sigma-Aldrich, UK) were ground to fine powder and mixed with 2–3 mL anhydrous dimethyl sulfoxide (Romil, UK) before adding to each dried sample. This was followed by the addition of 0.5–0.7 mL of methyl iodide (Alfa Aeser, UK) and vigorous shaking at room temperature for 15 min. Permethylated glycans were extracted with chloroform and then purified by using Sep-Pak C_18_ cartridges. The cartridges were successively conditioned with methanol (5 mL), water (5 mL), acetonitrile (5 mL) and water (15 mL). Each sample was dissolved in 200 µL of methanol:water (1∶1) solution before loading onto the cartridges. The cartridges were washed with 5 mL of water and then eluted sequentially with 3 mL of each 15, 35 and 50% acenotrile solution in water. All eluents were then concentrated with a Savant SpeedVac and subsequently lyophilized.

#### MS and MS/MS analyses of permethylated glycans

MALDI-TOF data were acquired on a Voyager-DE STR mass spectrometer (Applied Biosystems, Foster City, CA) in the reflectron mode with delayed extraction. Permethylated samples were dissolved in 10 µL of methanol, and 1 µL of dissolved sample was premixed with 1 µL of matrix (20 mg/ml 2,5-dihydroxybenzoic acid in 70% (v/v) aqueous methanol), spotted onto a target plate, and dried under vacuum. Further MS/MS analyses of peaks observed in the MS spectra were carried out using a 4800 MALDI-TOF/TOF (Applied Biosystems) mass spectrometer in positive ion mode (M + Na)+. The collision energy was set to 1 kV, and argon was used as collision gas. Samples were dissolved in 10 µL of methanol, and 1 µL was mixed at a 1∶1 ratio (v/v) with 2,5-dihydroxybenzoic acid (20 mg/mL in 70% methanol in water) as matrix.

#### Analyses of MALDI data

The MS and MS/MS data were processed using Data Explorer 4.9 Software (Applied Biosystems, U.K.). The mass spectra were baseline corrected (default settings) and noise filtered (with correction factor of 0.7), and then converted to ASCII format. The processed spectra were then subjected to manual assignment and annotation with the aid of a glycobioinformatics tool known as GlycoWorkBench [75]. Peak picking was done manually, and proposed assignments for the selected peaks were based on molecular mass composition of the 12C isotope together with knowledge of the biosynthetic pathways. Some of the proposed structures were then confirmed by data obtained from MS/MS experiment.

### Rabbit challenge

31 New Zealand white rabbits of 12 weeks were grouped into three groups of 10 or 11 rabbits and placed in 3 rooms (2 cages containing 5 rabbits per room) at BSL2 experimental facilities with filtered air according to biosafety and bioethical procedures. The experimental study was performed under the authorization of animal experimentation number 10/03/09-05B delivered by the ethics committee ComEth Anses/ENVA/UPEC. Six rabbits were sampled for pre-challenged serum just prior to infection. The rabbits were orally infected with 10^5^, 10^7^ or 10^9^ genome copies of the G4 virus 95–10 [17] in a total of 1 mL PBS. At the time of infection the rabbits were 12 weeks of age. As rabbits succumbed to infection, post-mortem examinations were realized and liver and duodenum were sampled in RNAlater (Sigma-Aldrich, St. Louis, MO). Surviving animals were killed humanely after 11 days, examined for macroscopic lesions, and tissue samples were collected. Liver and duodenum scrapings were analyzed for RHDV quantification as described above. Duodenum was also phenotyped for ABH expression as described above and tested for G4 binding, also described above.

### Accession numbers

Fut2 XM_00273737

Sec1 X80225

Fut1 NM_001082403

G1 JF438967

G2 FR823355

G3 FR823354

G4 AJ535094

G5 AM085133

G6 AJ969628

## Supporting Information

Figure S1 **MALDI-TOF MS of N-glycans released by PNGase F digestion from three of the Rabbit Duodenum samples analyzed.** Glycan were permethylated prior to MALDI-TOF analysis. Structures were assigned taking into account the molecular weight and the biosynthetic pathway. A structure containing monosaccharides outside a bracket suggests potential structural heterogeneity of the peak. “X” indicates peaks corresponding to high mannose N-glycans. A: Profile of permethylated N-glycans released from rabbit duodenum, Sample 2. B: Profile of permethylated N-glycans released from rabbit duodenum, Sample 4. C: Profile of permethylated N-glycans released from rabbit duodenum, Sample 6.
(PDF)

Figure S2 **Representative MALDI-TOF-TOF mass spectra of O-glycans released from three of the Rabbit Duodenum samples analysed (Samples 2, 4, 6).** Glycans released by β-elimination reaction were permethylated prior to MALDI-TOF-TOF analysis. The fragment ions are consistent with the sequences shown in the inset. **A**: MS/MS spectrum of the molecular ion at m/z 708, sample 2; **B**: MS/MS spectrum of the molecular ion at m/z 953, sample 2; **C**: MS/MS spectrum of the molecular ion at m/z 1128, sample 2; **D**: MS/MS spectrum of the molecular ion at m/z 1199, sample 2; **E**: MS/MS spectrum of the molecular ion at m/z 1332, sample 2; **F**: MS/MS spectrum of the molecular ion at m/z 1404, sample 2; **G**: MS/MS spectrum of the molecular ion at m/z 1578, sample 2; **H**: MS/MS spectrum of the molecular ion at m/z 708, sample 4; **I**: MS/MS spectrum of the molecular ion at m/z 912, sample 4; **J**: MS/MS spectrum of the molecular ion at m/z 954, sample 4; **K**: MS/MS spectrum of the molecular ion at m/z 1199, sample 4; **L**: MS/MS spectrum of the molecular ion at m/z 1373, sample 4; **M**: MS/MS spectrum of the molecular ion at m/z 708, sample 6; **N**: MS/MS spectrum of the molecular ion at m/z 912, sample 6; **O**: MS/MS spectrum of the molecular ion at m/z 953, sample 6; **P**: MS/MS spectrum of the molecular ion at m/z 1128, sample 6; **Q**: MS/MS spectrum of the molecular ion at m/z 1158, sample 6; **R**: MS/MS spectrum of the molecular ion at m/z 1199, sample 6; **S**: MS/MS spectrum of the molecular ion at m/z 1331, sample 6; **T**: MS/MS spectrum of the molecular ion at m/z 1373, sample 6.
(PDF)

Figure S3 **MALDI-Ion Trap-TOF MS, MS/MS and MS^3^ mass spectra of O-glycans released from the Rabbit Duodenum Sample 4.** The sample was kindly analyzed by Shimadzu Ltd, by their MALDI-Ion Trap-TOF instrument “Axima Resonance”. A: MS spectrum of sample 4. The annotation of the structure at m/z 1373 takes into account the information emerging from fragmentation data obtained through MS/MS and MS^3^ analysis. B: MS/MS spectrum of peak at m/z 1373. Theoretical fragments, shown at the top- right corner of the figure, match the observed ones obtained by Ion trap-MS/MS analysis, reported on the annotated peaks. Peaks marked with the symbol “X” are due to the matrix used for MALDI analysis. C: MS^3^ spectra of the ion at m/z 1079 obtained after fragmentation of the parent ion at m/z 1373. Theoretical fragments, shown at the top- right corner of the figure, match the observed fragments obtained by Ion trap-MS^3^ fragmentation, reported on the annotated peaks. Peaks marked with the symbol “X” are due to the matrix used for MALDI analysis.
(PDF)

Figure S4 **RHDV challenge of A+B+ and A−B− rabbits. 31 rabbits were infected with an A and B binding G4 strain at 10^7^ or 10^9^ virus copies.** Rabbits either succumbed to infection or were sacrificed after 11 days. Duodenum and liver samples were collected at time of death. Duodenum was phenotyped for G4 binding. Duodenum and liver were also assayed for virus RNA via real time RT-PCR. (A–B) Virus RNA of liver or duodenum for 10^7^ infectious dose, (C–D) virus RNA of liver or duodenum for 10^9^ infectious dose, where A+B+ (circle), A−B− (triangle), dead rabbits (black symbol) and survival rabbits (white symbol).
(PDF)

Figure S5 **Survival analysis of rabbits challenged with the G4 strain at 3 genome copies doses (10^5^, 10^7^ and 10^9^).** At 11 days, all survivors were sacrificed. There were no statistically significant differences between the 3 groups.
(PDF)

Table S1 **Neoglycoconjugates used to determine the carbohydrate binding characteristics of RHDV strains.** ^a^Oligosaccharides were used coupled to either polyacrylamide via an 3 carbon spacer (R1), or to human serum albumin via either a p-aminophenylethyl spacer (R2) or an acetyl phenylenediamine spacer (R3).
(PDF)

Table S2 **Contingency table of relationship between expression of H type 2 with A or B.** ^1^p = 4.8×10^−7^; ^2^p = 7.5×10^−6^ ; ^3^p = 2.0×10^−9^.
(PDF)

Table S3 **Summary of all structures observed in the MALDI-TOF-MS spectra of the duodenum tissue from rabbits, Samples 1**–**10.** The symbol presentation of O-glycans is based on the nomenclature used in Essentials of Glycobiology textbook (http://www.ncbi.nlm.nih.gov/books/NBK1908) and adopted by the Consortium for Functional Glycomics (http://www.functionalglycomics.org). Mass values [M + Na]+, cartoon representation of structure and epitope types are reported for each peak detected. Intensity of the peaks observed is reported for each sample analyzed. ND  =  not detected –  =  very minor component (<25%); -  =  minor component (25–50%); **+**  =  major component (>25%); **++**  =  very major component (25–50%); **+++**  =  most abundant component (>50%).
(PDF)

Table S4 **Composition of permethylated O-glycans in 10 samples of Rabbit Duodenum, obtained through MALDI-TOF analysis.**
(PDF)

Table S5 **Significance of correlation between RHDV binding and ABH phenotypes with Fischer's exact test.** ^1^ 7 rabbits A+B-H− and one A+B-H+ with weak H type 2 expression.
(PDF)
