# Supplementary material for: Histo-Blood Group Antigens Act as Attachment Factors of Rabbit Hemorrhagic Disease Virus Infection in a Virus Strain-Dependent Manner
Source: PLoS Pathog. 2011 Aug 25;7(8):e1002188. doi: 10.1371/journal.ppat.1002188 (PMC3161982; doi:10.1371/journal.ppat.1002188)
Supplement: Figure S1 — MALDI-TOF MS of N-glycans released by PNGase F digestion from three of the Rabbit Duodenum samples analyzed. Glycan were permethylated prior to MALDI-TOF analysis. Structures were assigned taking into account the molecular weight and the biosynthetic pathway. A structure containing monosaccharides outside a bracket suggests potential structural heterogeneity of the peak. “X” indicates peaks corresponding to high mannose N-glycans. A: Profile of permethylated N-glycans released from rabbit duodenum, Sample 2. B: Profile of permethylated N-glycans released from rabbit duodenum, Sample 4. C: Profile of permethylated N-glycans released from rabbit duodenum, Sample 6. (PDF) [file ppat.1002188.s001.pdf]

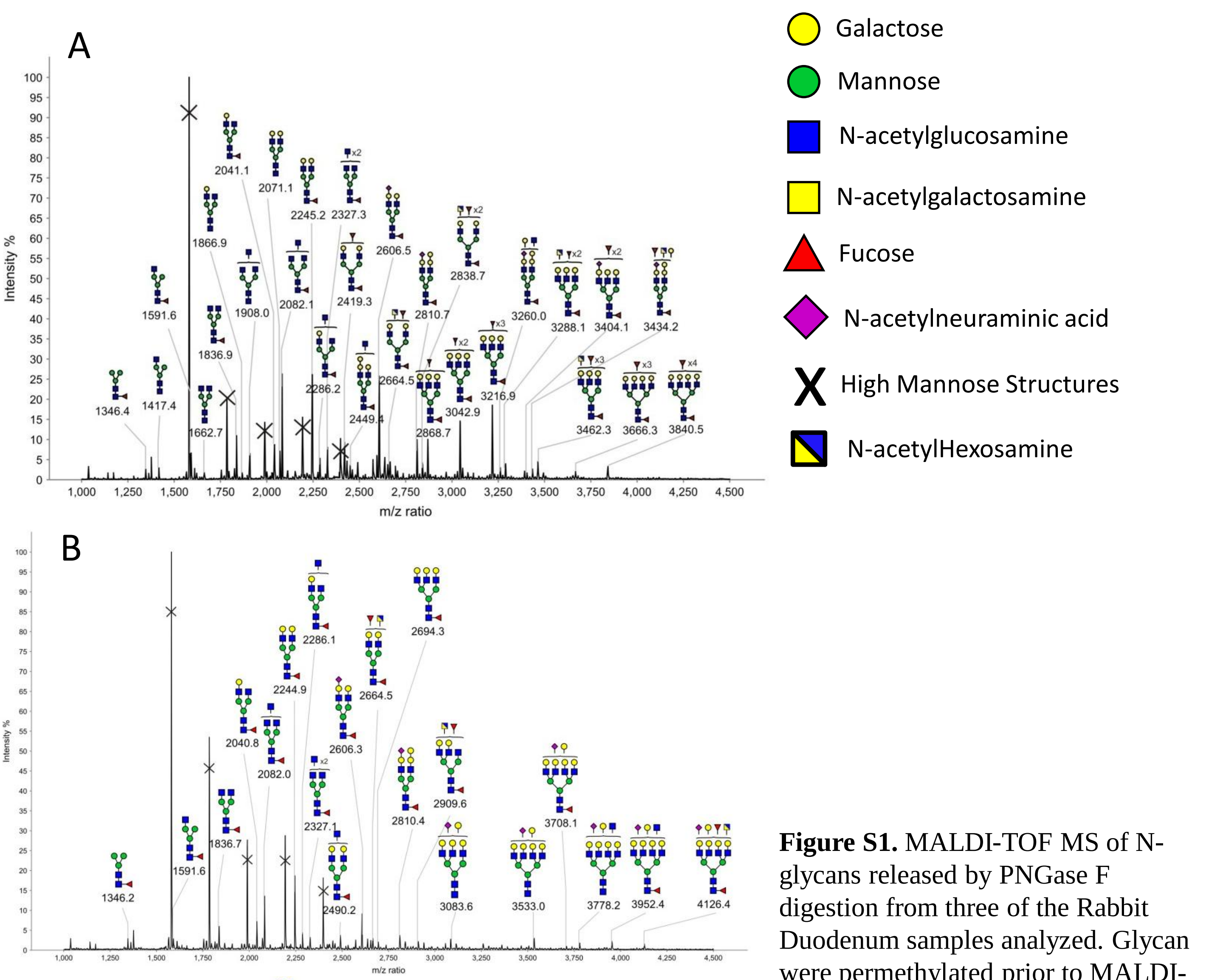

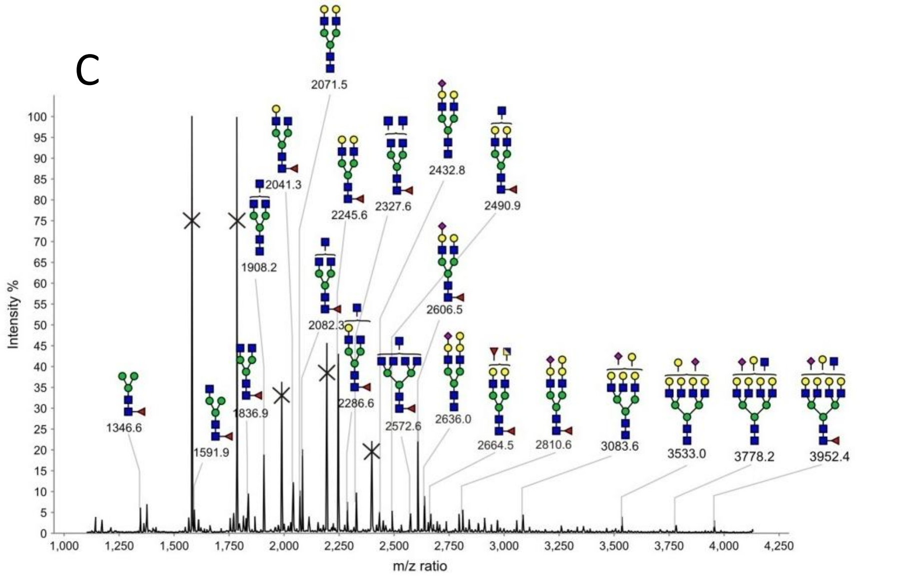

Figure S1. MALDI-TOF MS of N-glycans released by PNGase F digestion from three of the Rabbit Duodenum samples analyzed. Glycan were permethylated prior to MALDI-TOF analysis. Structures were assigned taking into account the molecular weight and the biosynthetic pathway. A structure containing monosaccharides outside a bracket suggests potential structural heterogeneity of the peak. "X" indicates peaks corresponding to high mannose N-glycans.

A: Profile of permethylated N-glycans released from rabbit duodenum, Sample 2.

B: Profile of permethylated N-glycans released from rabbit duodenum, Sample 4.

C: Profile of permethylated N-glycans released from rabbit duodenum, Sample 6.
