# Supplementary material for: Histo-Blood Group Antigens Act as Attachment Factors of Rabbit Hemorrhagic Disease Virus Infection in a Virus Strain-Dependent Manner
Source: PLoS Pathog. 2011 Aug 25;7(8):e1002188. doi: 10.1371/journal.ppat.1002188 (PMC3161982; doi:10.1371/journal.ppat.1002188)
Supplement: Figure S2 — Representative MALDI-TOF-TOF mass spectra of O-glycans released from three of the Rabbit Duodenum samples analysed (Samples 2, 4, 6). Glycans released by β-elimination reaction were permethylated prior to MALDI-TOF-TOF analysis. The fragment ions are consistent with the sequences shown in the inset. A: MS/MS spectrum of the molecular ion at m/z 708, sample 2; B: MS/MS spectrum of the molecular ion at m/z 953, sample 2; C: MS/MS spectrum of the molecular ion at m/z 1128, sample 2; D: MS/MS spectrum of the molecular ion at m/z 1199, sample 2; E: MS/MS spectrum of the molecular ion at m/z 1332, sample 2; F: MS/MS spectrum of the molecular ion at m/z 1404, sample 2; G: MS/MS spectrum of the molecular ion at m/z 1578, sample 2; H: MS/MS spectrum of the molecular ion at m/z 708, sample 4; I: MS/MS spectrum of the molecular ion at m/z 912, sample 4; J: MS/MS spectrum of the molecular ion at m/z 954, sample 4; K: MS/MS spectrum of the molecular ion at m/z 1199, sample 4; L: MS/MS spectrum of the molecular ion at m/z 1373, sample 4; M: MS/MS spectrum of the molecular ion at m/z 708, sample 6; N: MS/MS spectrum of the molecular ion at m/z 912, sample 6; O: MS/MS spectrum of the molecular ion at m/z 953, sample 6; P: MS/MS spectrum of the molecular ion at m/z 1128, sample 6; Q: MS/MS spectrum of the molecular ion at m/z 1158, sample 6; R: MS/MS spectrum of the molecular ion at m/z 1199, sample 6; S: MS/MS spectrum of the molecular ion at m/z 1331, sample 6; T: MS/MS spectrum of the molecular ion at m/z 1373, sample 6. (PDF) [file ppat.1002188.s002.pdf]

Figure S2. Representative MALDI-TOF-TOF mass spectra of O-glycans released from three of the Rabbit Duodenum samples analysed (Samples 2, 4, 6).

Glycans released by β-elimination reaction were permethylated prior to MALDI-TOF-TOF analysis.

The fragment ions are consistent with the sequences shown in the inset.

A: MS/MS spectrum of the molecular ion at m/z 708, sample 2.
B: MS/MS spectrum of the molecular ion at m/z 953, sample 2.
C: MS/MS spectrum of the molecular ion at m/z 1128, sample 2.
D: MS/MS spectrum of the molecular ion at m/z 1199, sample 2.
E: MS/MS spectrum of the molecular ion at m/z 1332, sample 2.
F: MS/MS spectrum of the molecular ion at m/z 1404, sample 2.
G: MS/MS spectrum of the molecular ion at m/z 1578, sample 2.
H: MS/MS spectrum of the molecular ion at m/z 708, sample 4.
I: MS/MS spectrum of the molecular ion at m/z 912, sample 4.
J: MS/MS spectrum of the molecular ion at m/z 954, sample 4.
K: MS/MS spectrum of the molecular ion at m/z 1199, sample 4.
L: MS/MS spectrum of the molecular ion at m/z 1373, sample 4.
M: MS/MS spectrum of the molecular ion at m/z 708, sample 6.
N: MS/MS spectrum of the molecular ion at m/z 912, sample 6.
O: MS/MS spectrum of the molecular ion at m/z 953, sample 6.
P: MS/MS spectrum of the molecular ion at m/z 1128, sample 6.
Q: MS/MS spectrum of the molecular ion at m/z 1158, sample 6.
R: MS/MS spectrum of the molecular ion at m/z 1199, sample 6.
S: MS/MS spectrum of the molecular ion at m/z 1331, sample 6.
T: MS/MS spectrum of the molecular ion at m/z 1373, sample 6.
U: MS/MS spectrum of the molecular ion at m/z 1404, sample 6.
V: MS/MS spectrum of the molecular ion at m/z 1444, sample 6.
X: MS/MS spectrum of the molecular ion at m/z 1578, sample 6.
Y: MS/MS spectrum of the molecular ion at m/z 1648, sample 6.
Z: MS/MS spectrum of the molecular ion at m/z 1823, sample 6.

Sample 2- msms 708

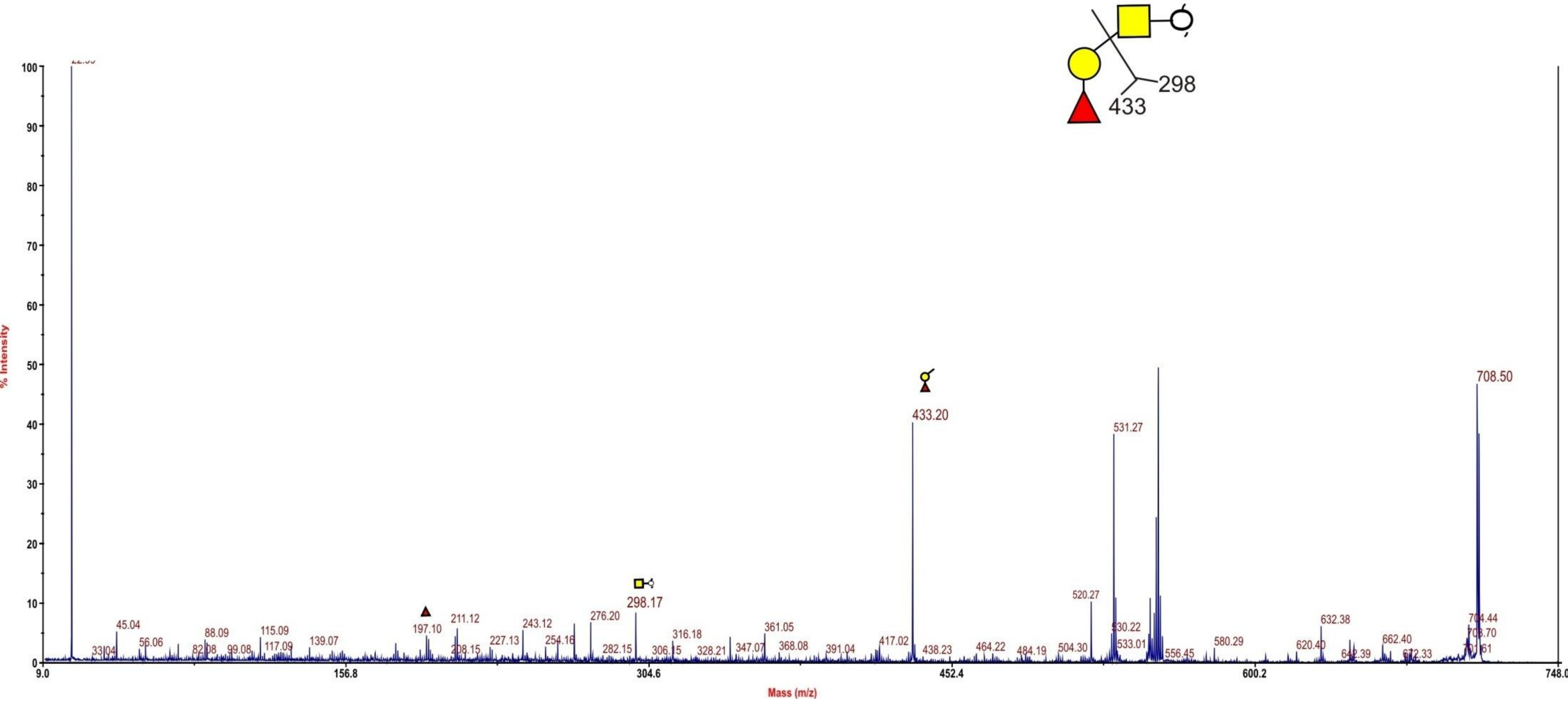

A

Sample 2- msms 953

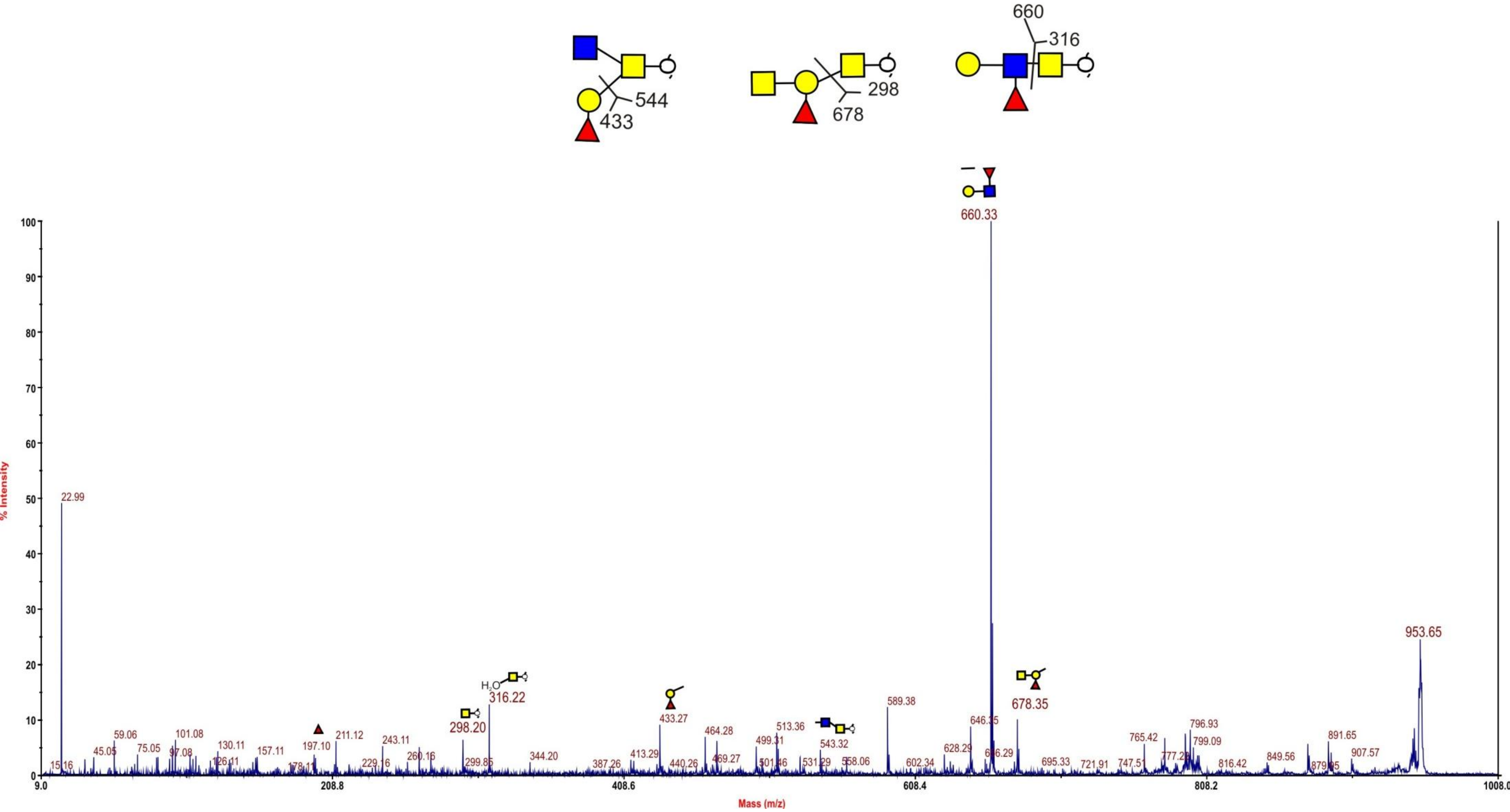

B

Sample 2- msms 1128

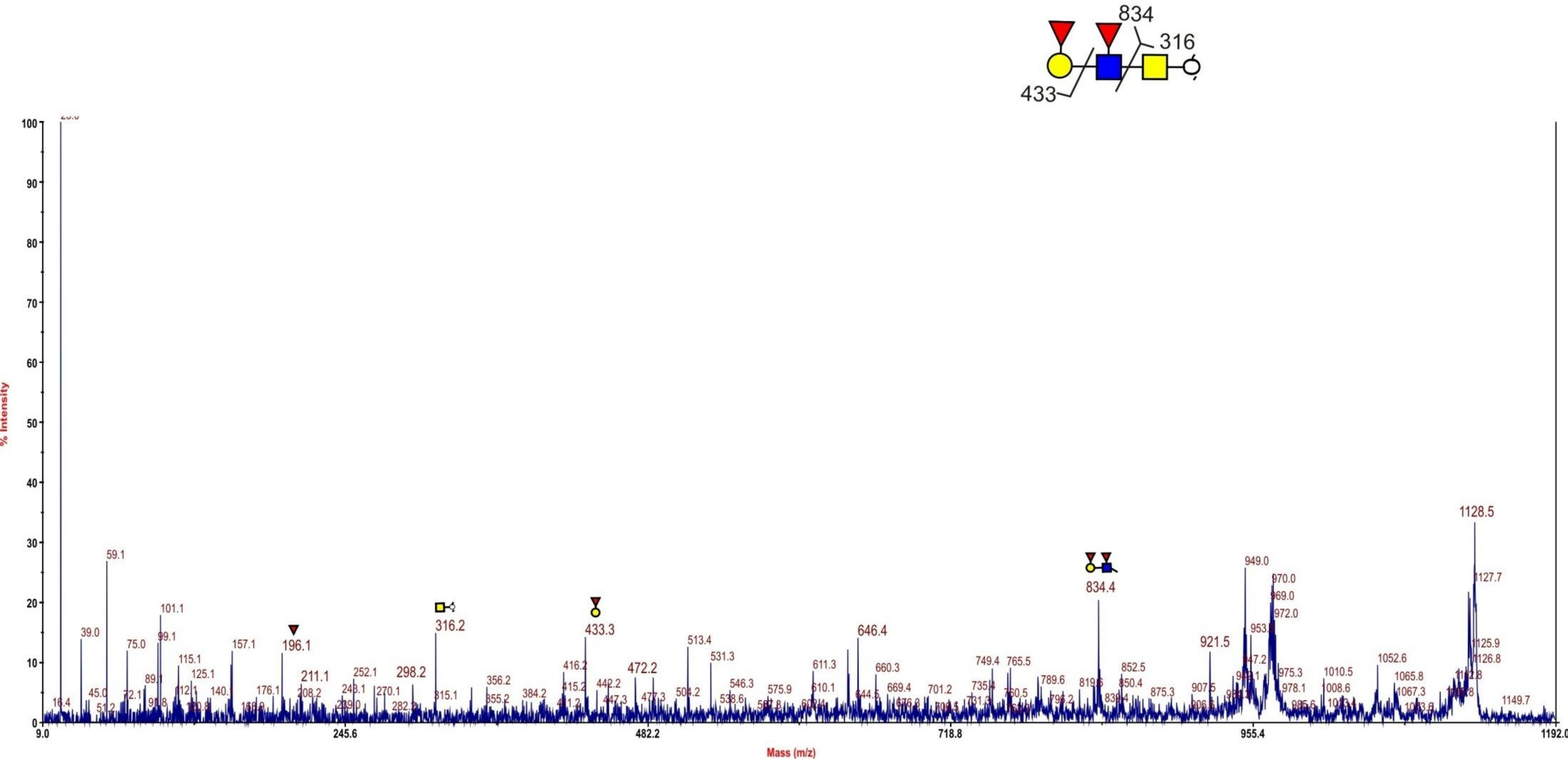

C

Sample 2- msms 1199

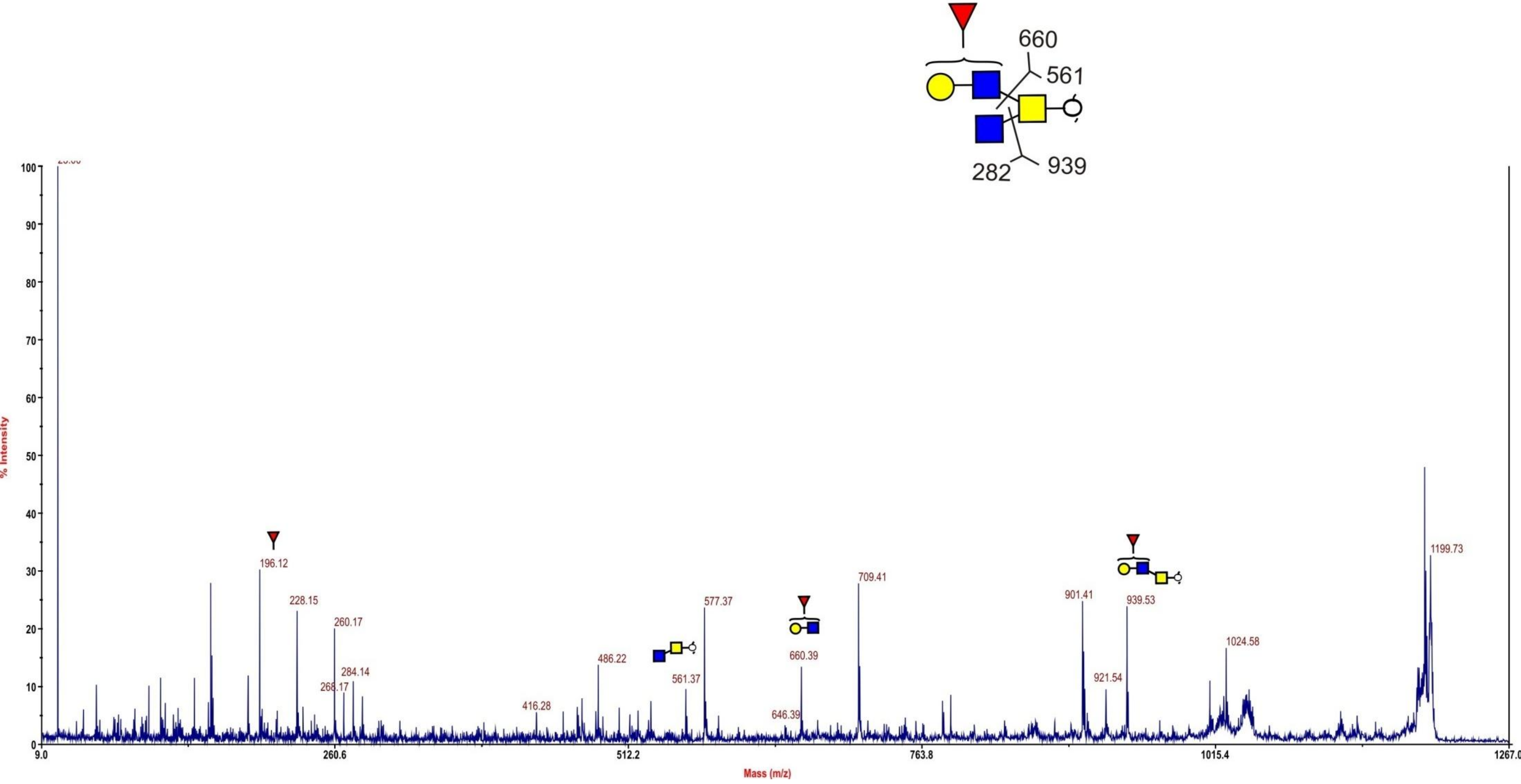

D

Sample 2- msms 1332

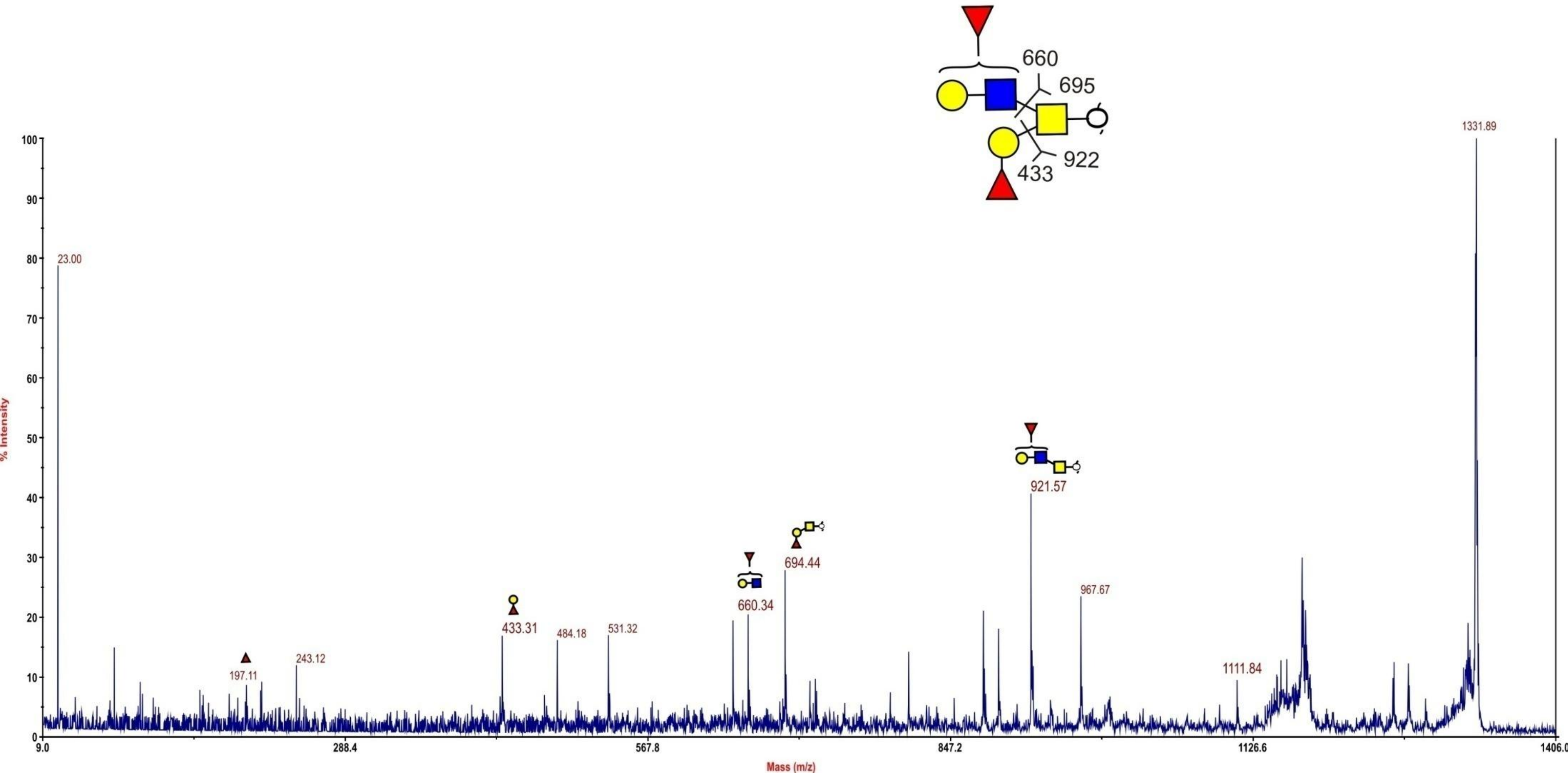

E

Sample 2- msms 1404

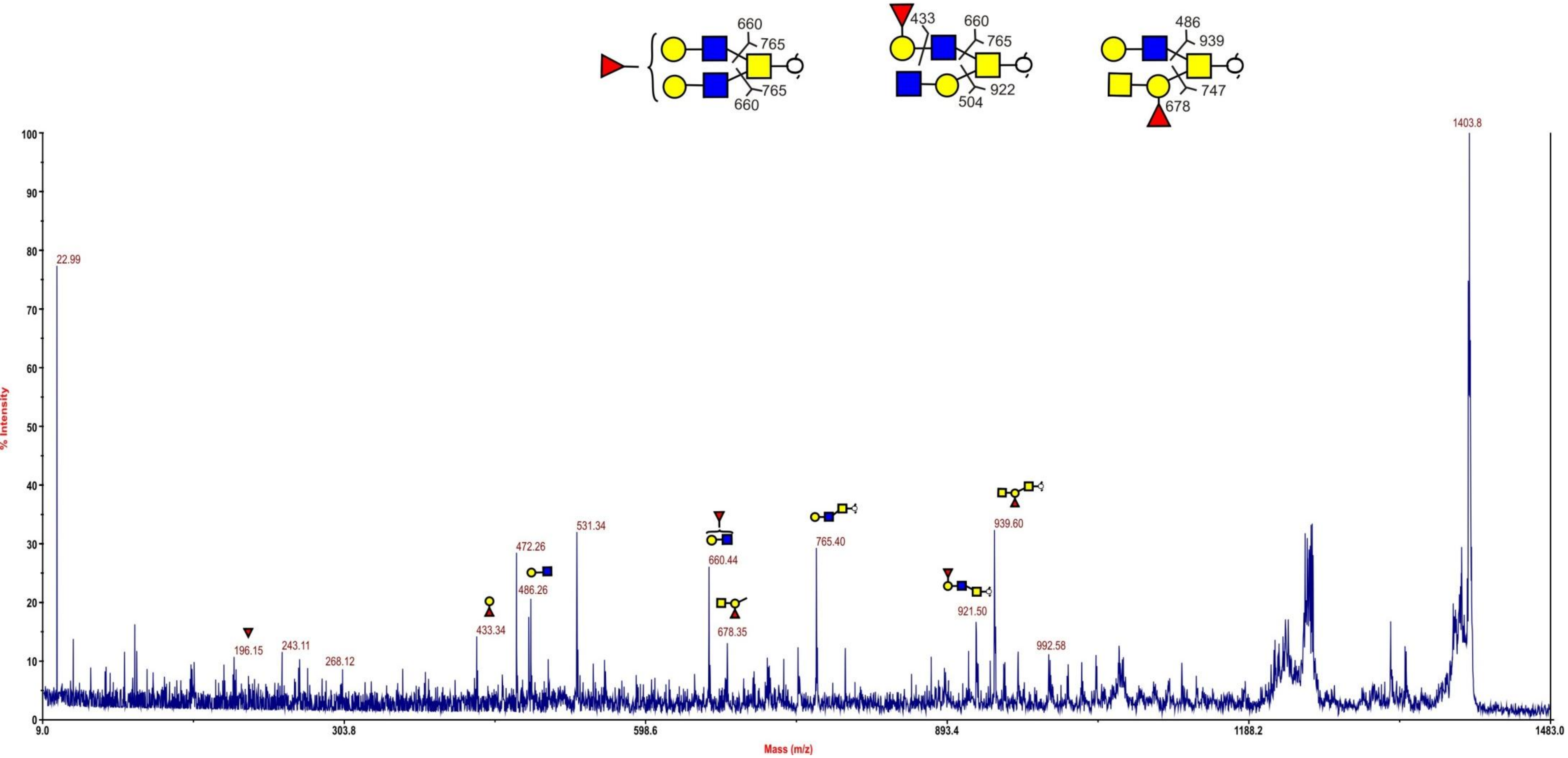

F

Sample 2- msms 1578

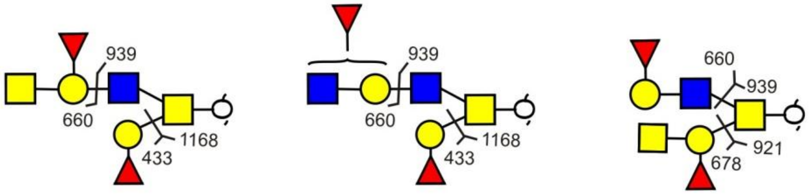

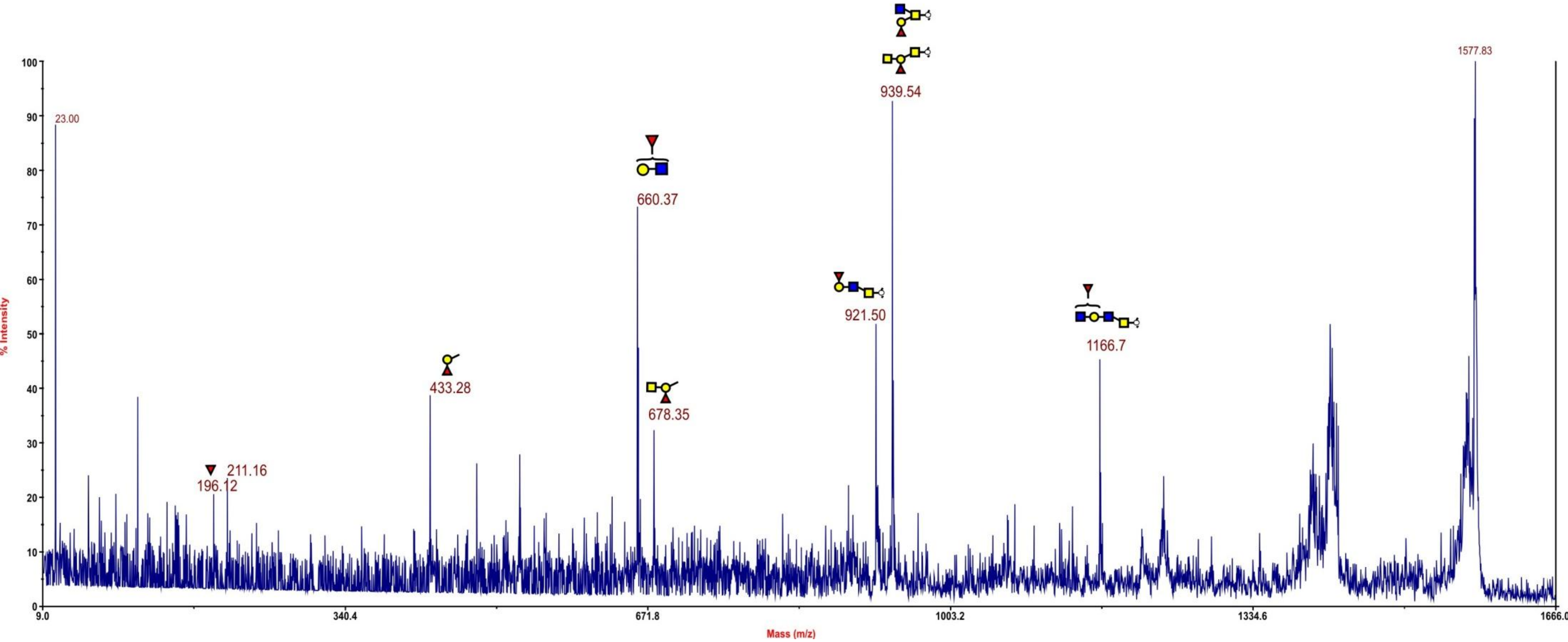

G

Sample 4- msms 708

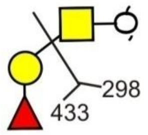

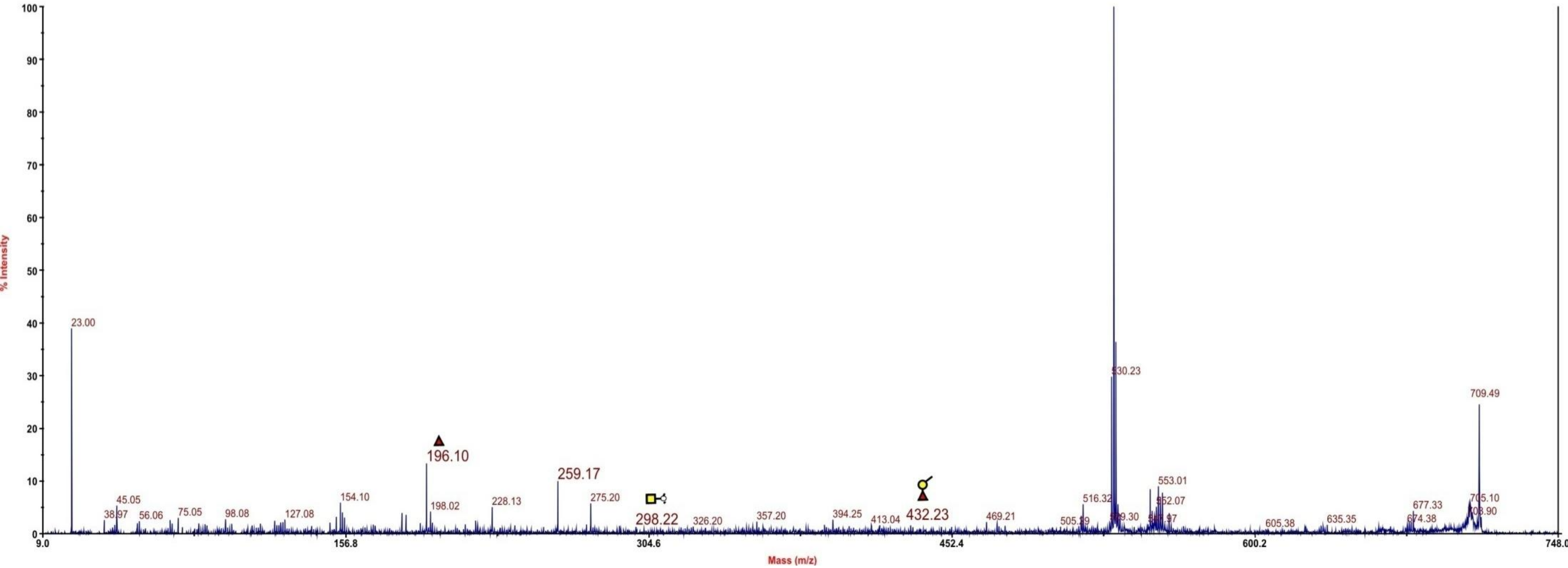

H

Sample 4- msms 912

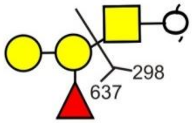

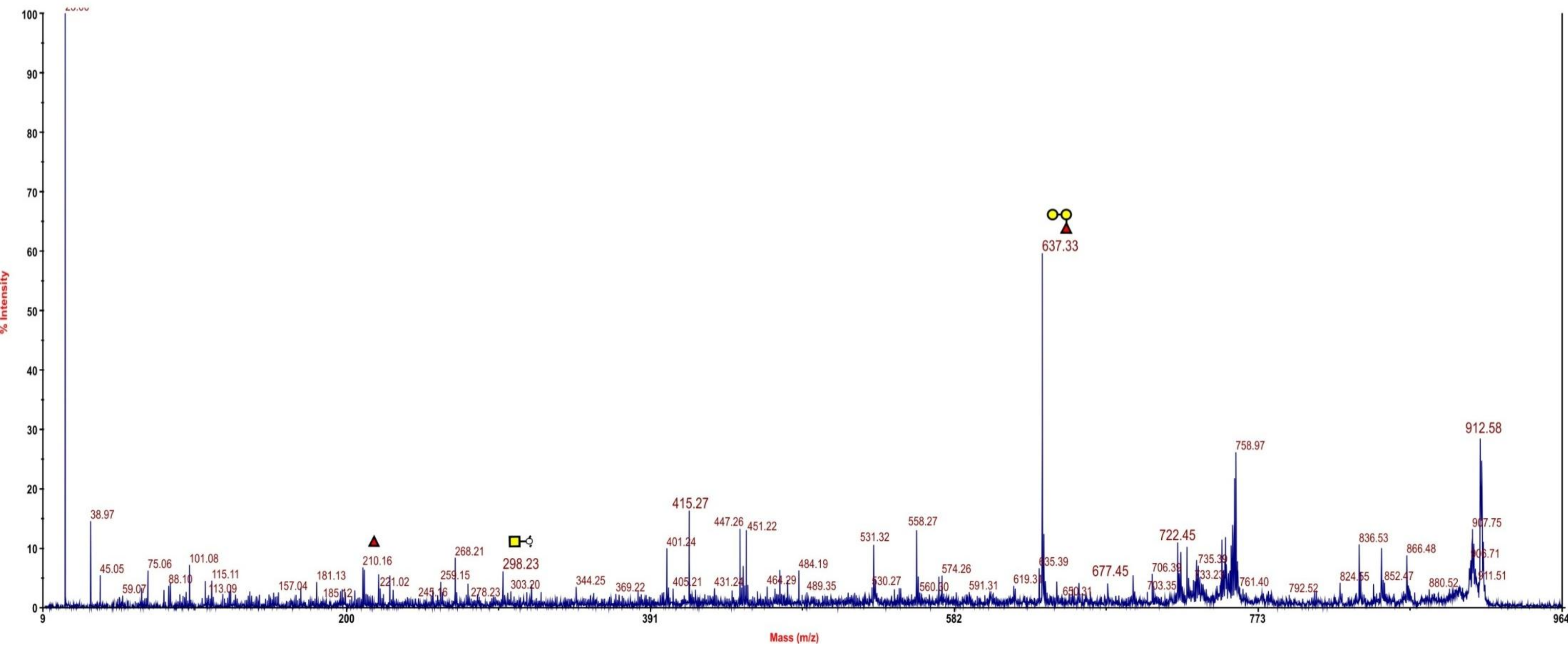

Sample 4- msms 954

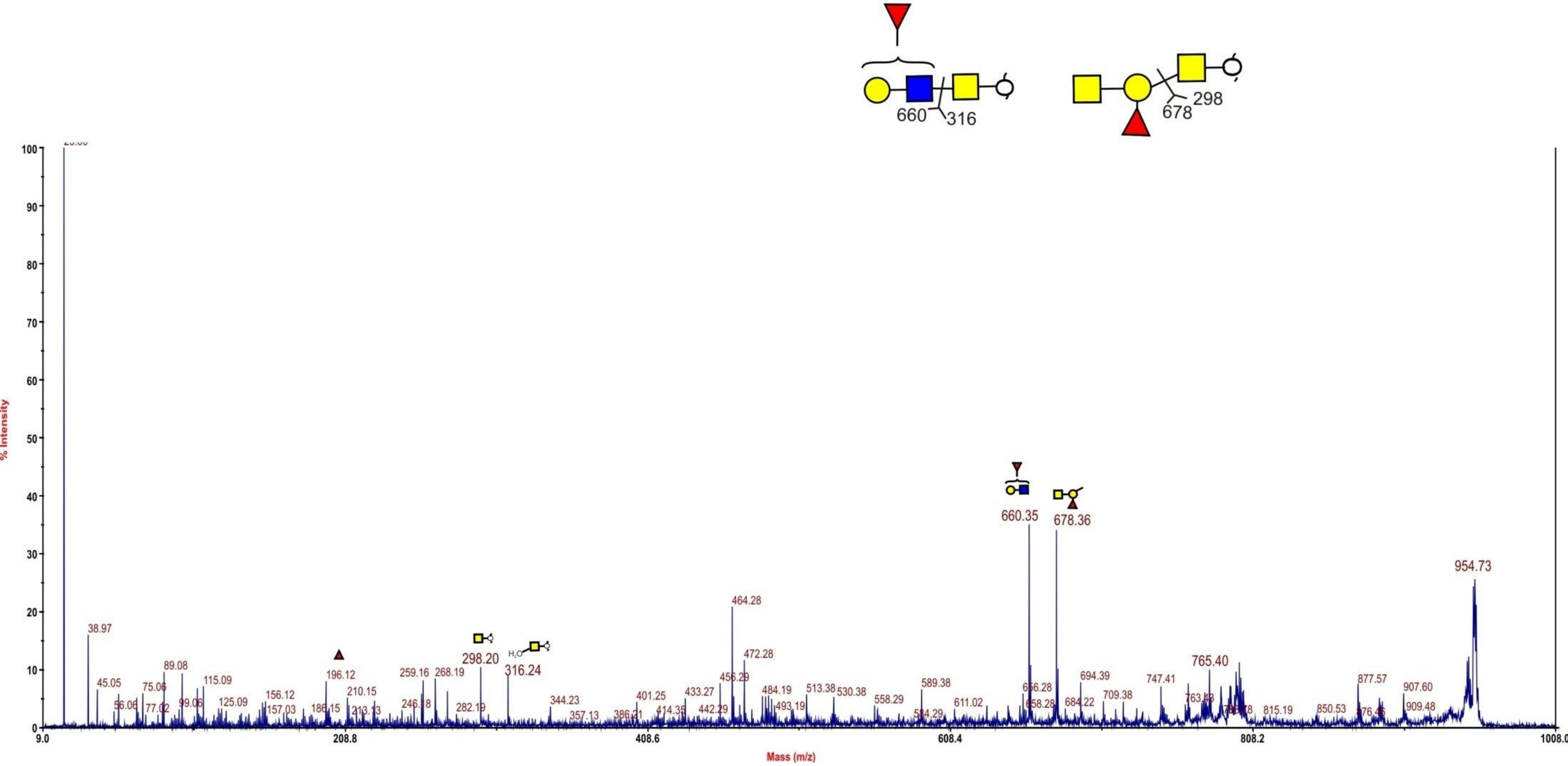

J

Sample 4- msms 1199

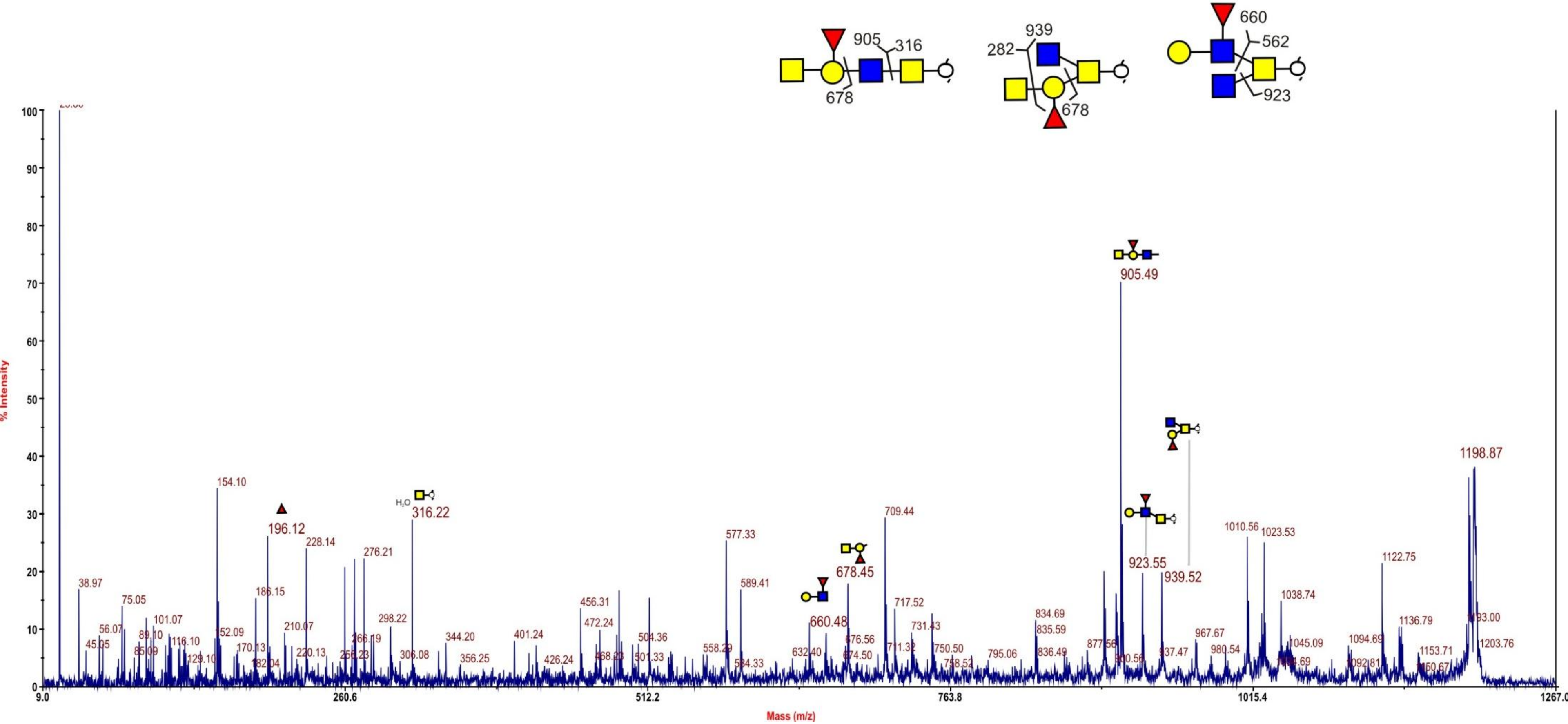

K

Sample 4- msms 1373

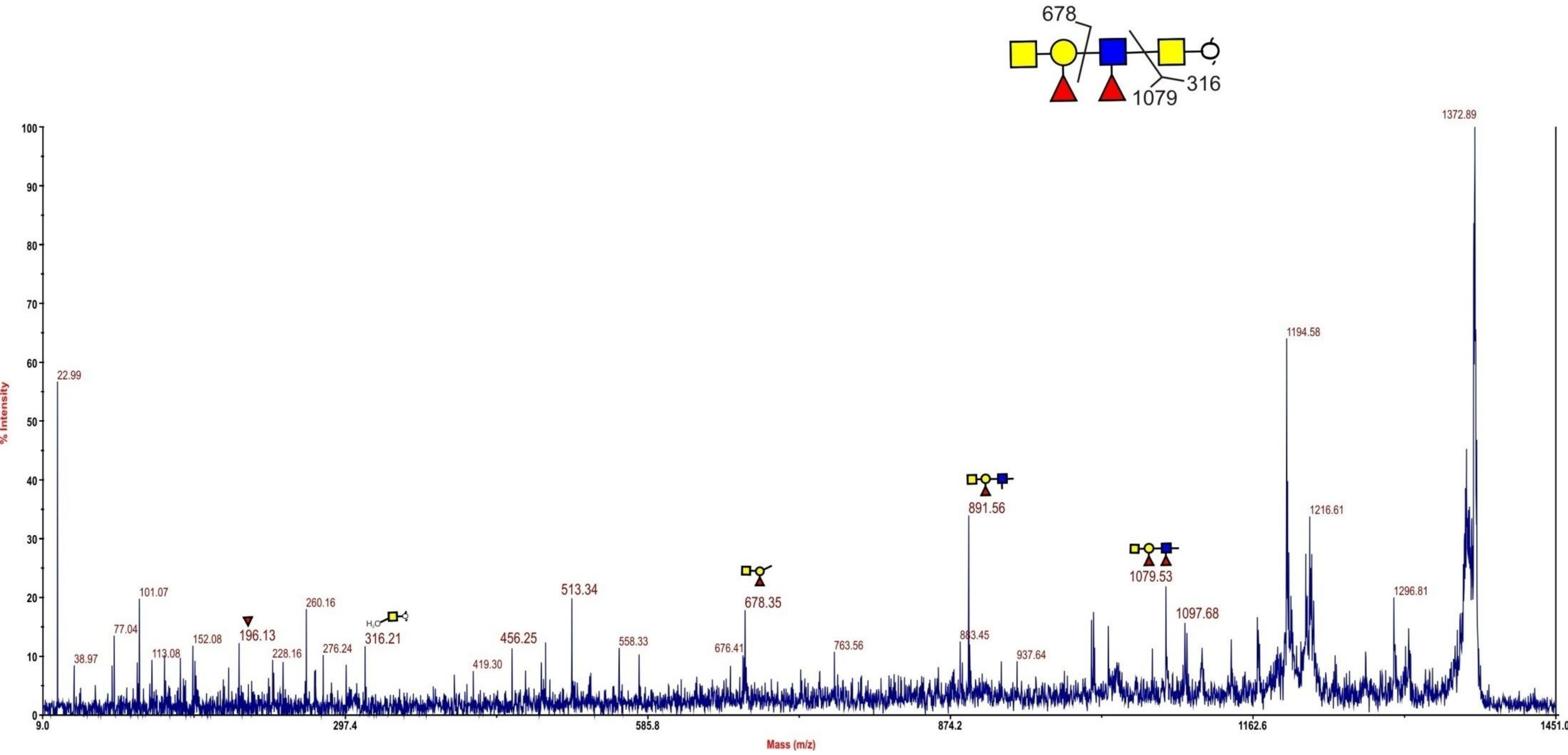

L

Sample 6- msms 708

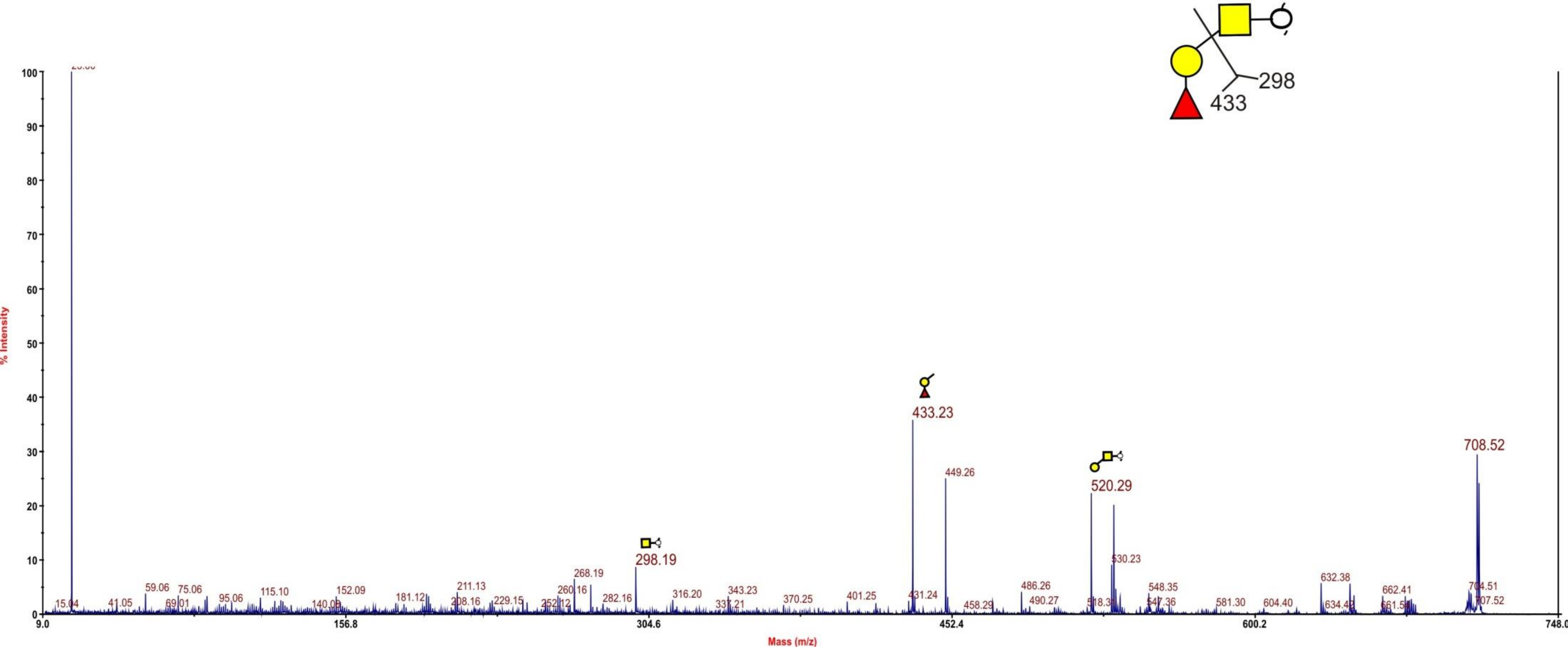

M

Sample 6- msms 912

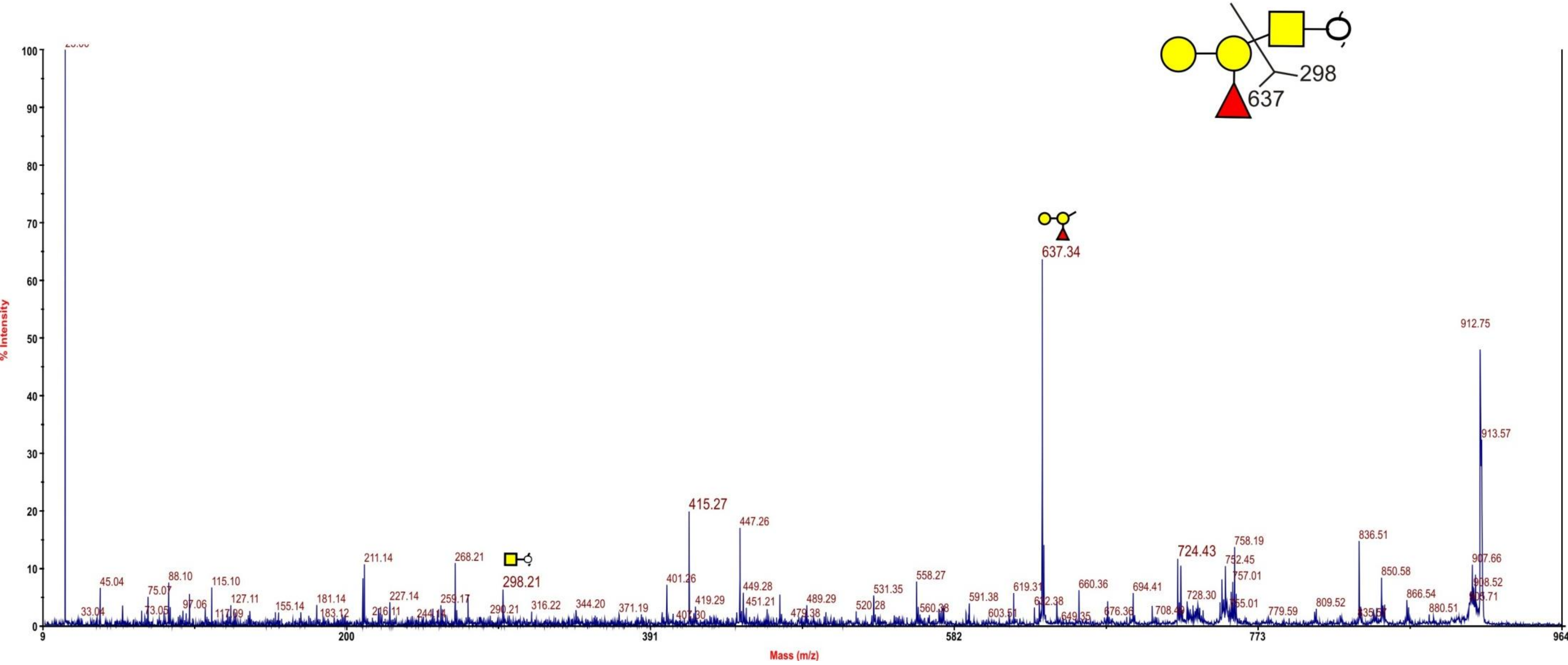

N

Sample 6- msms 954

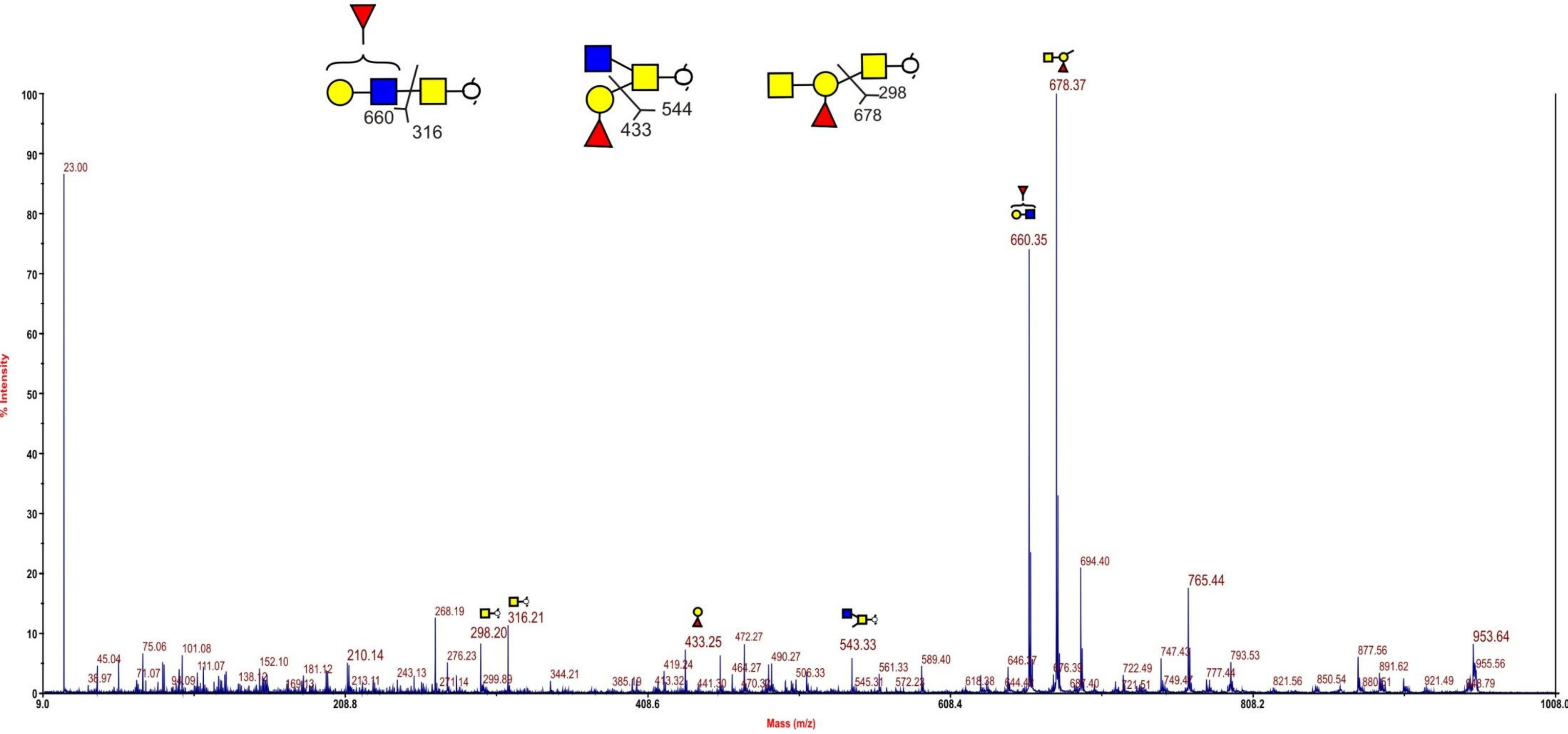

O

Sample 6- msms 1128

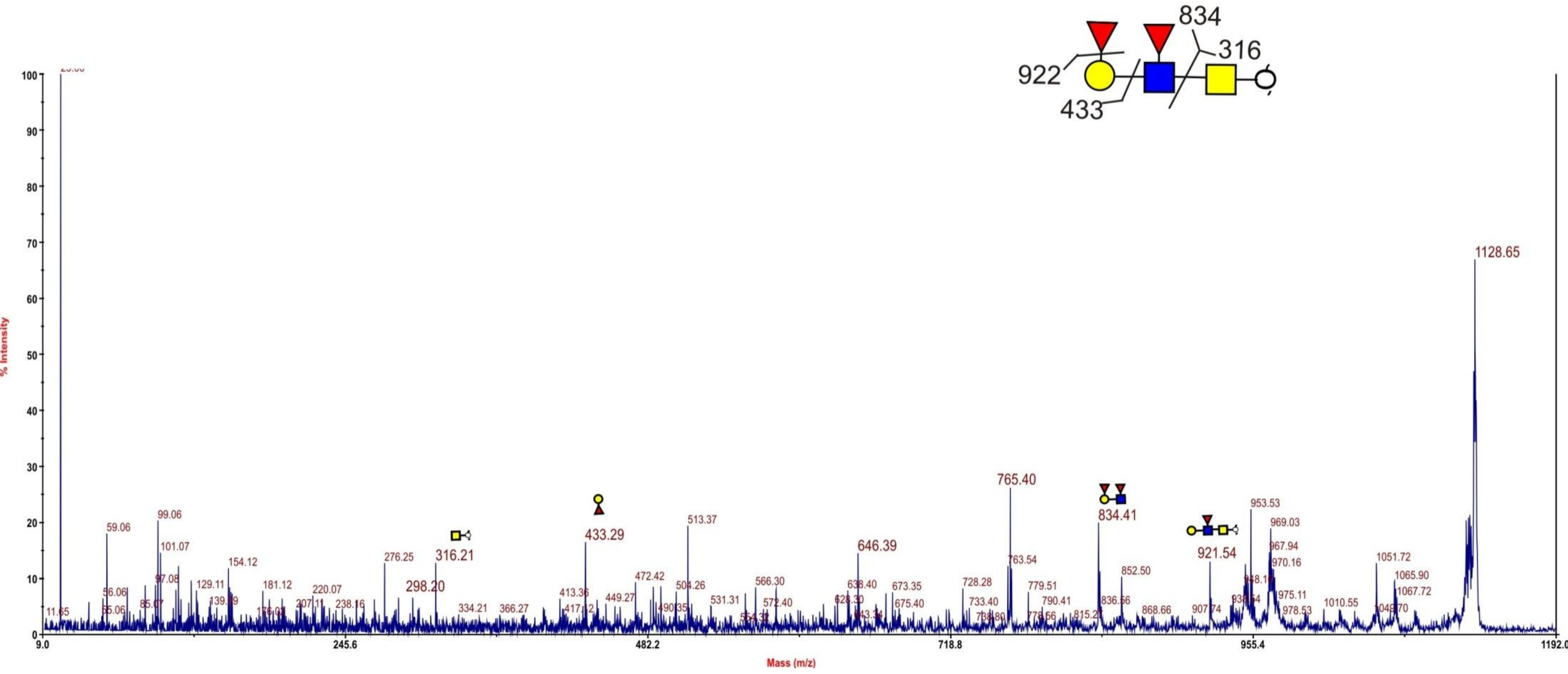

P

Sample 6- msms 1158

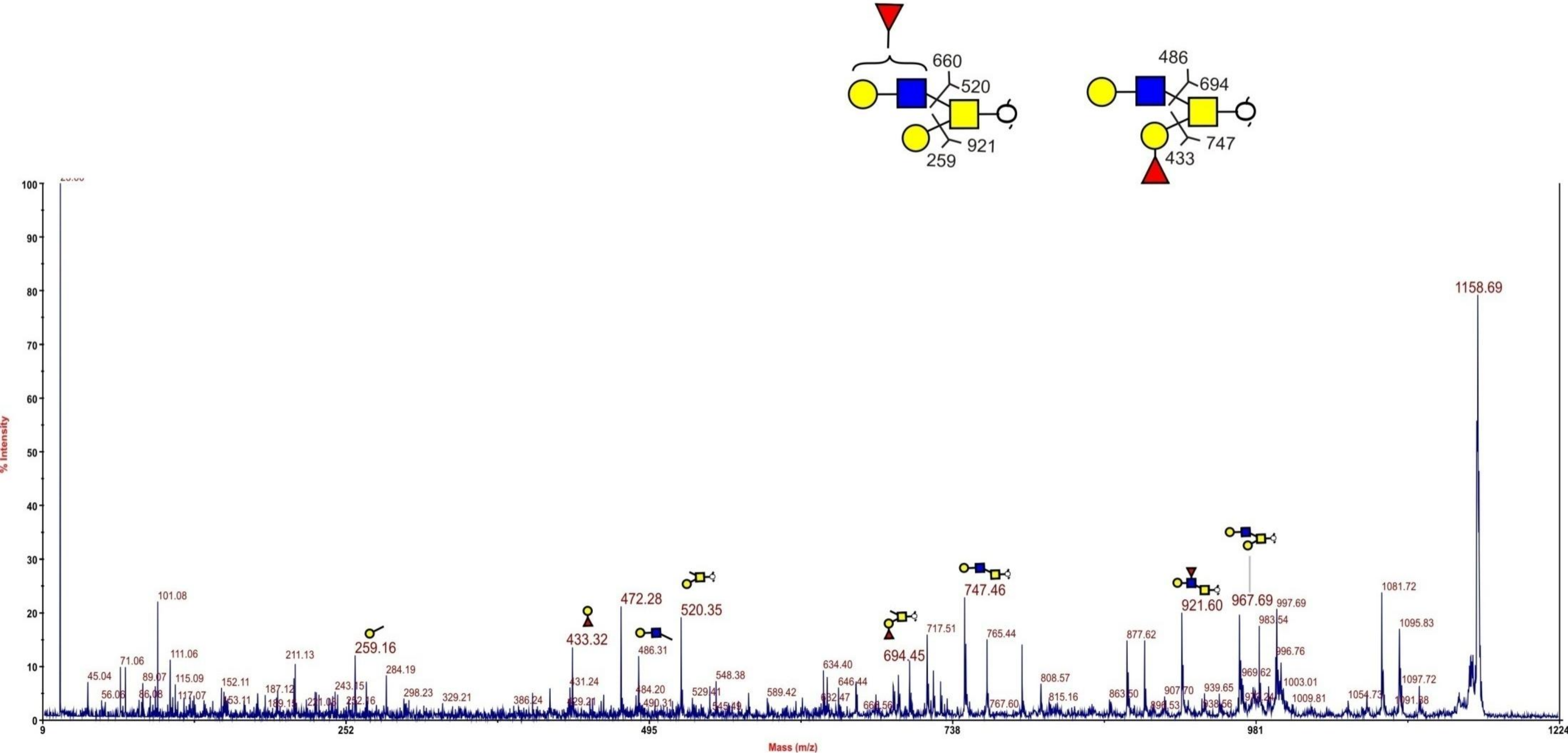

Q

Sample 6- msms 1199

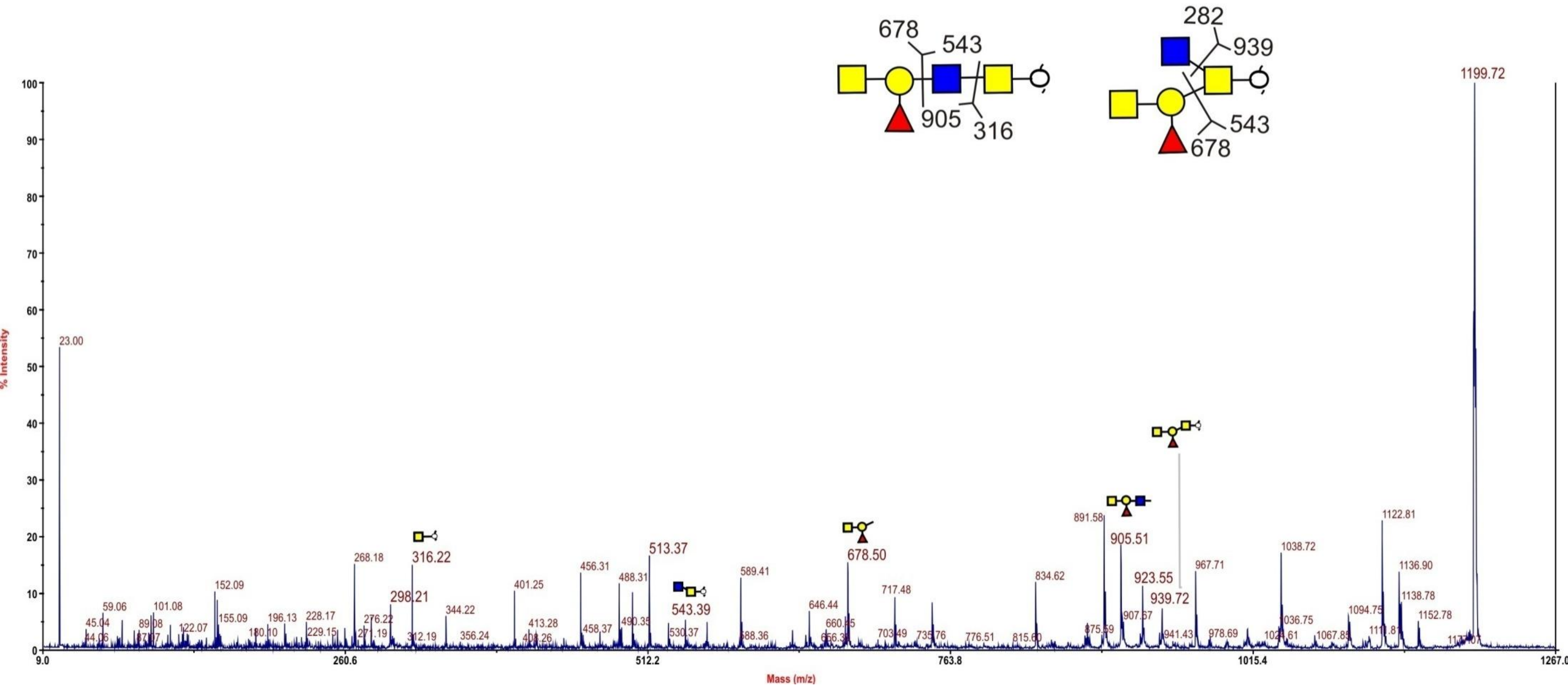

R

Sample 6- msms 1331

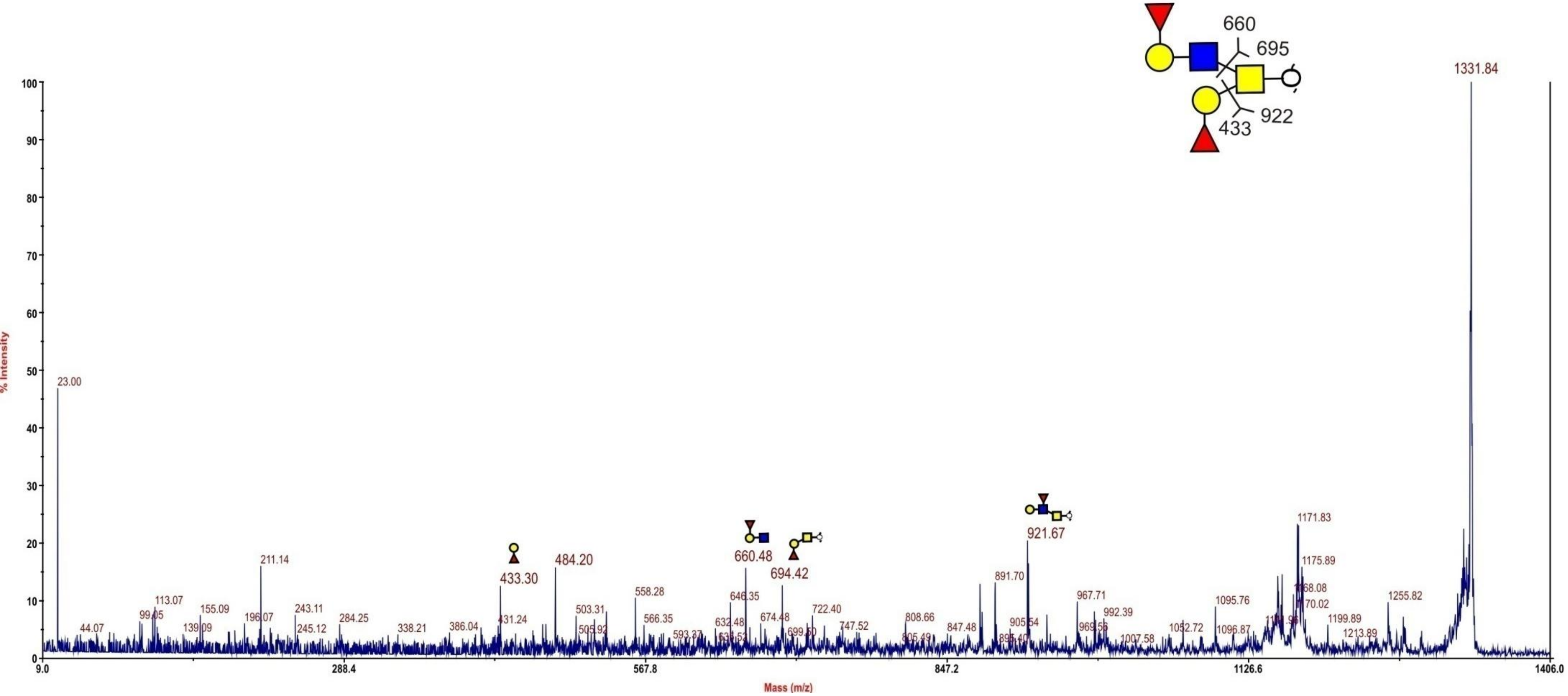

S

Sample 6- msms 1373

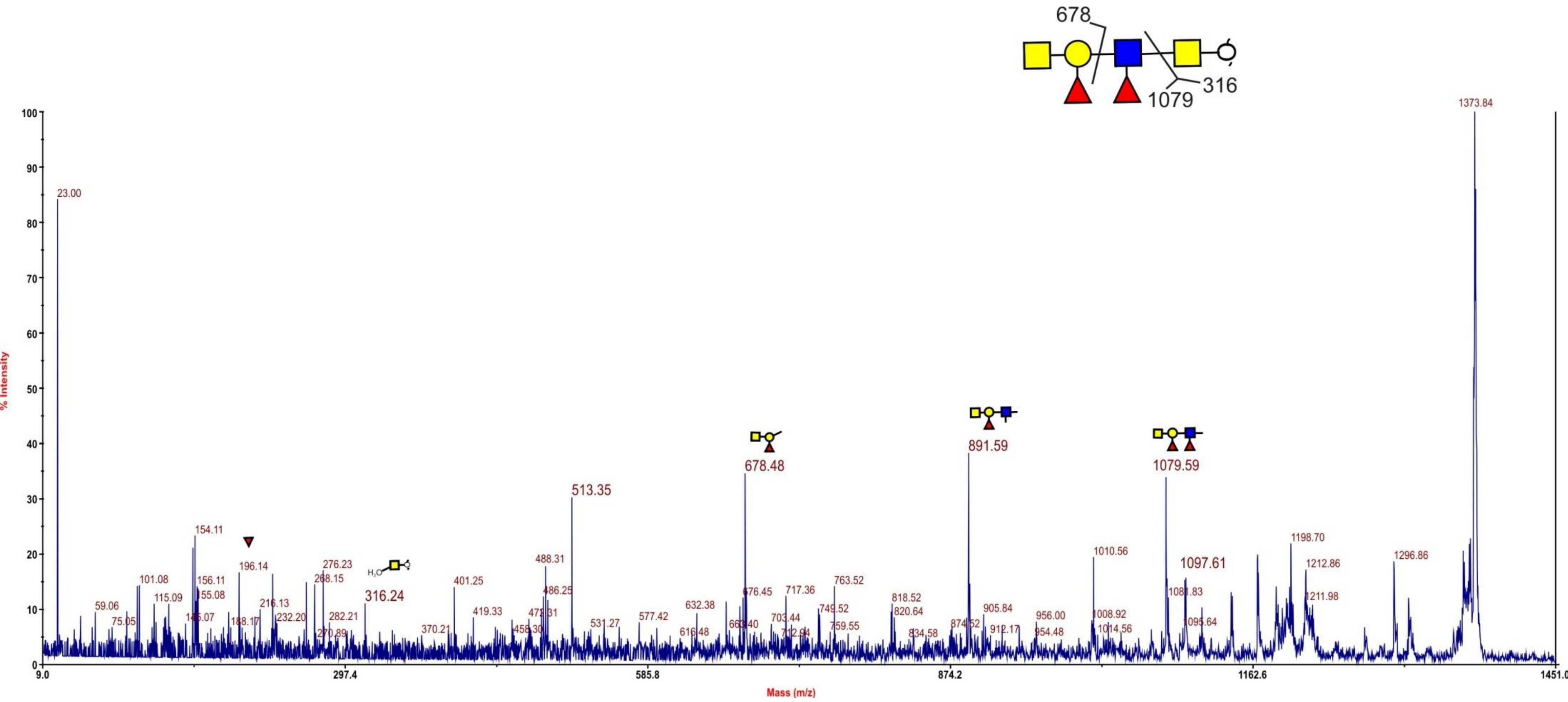

T

Sample 6- msms 1403

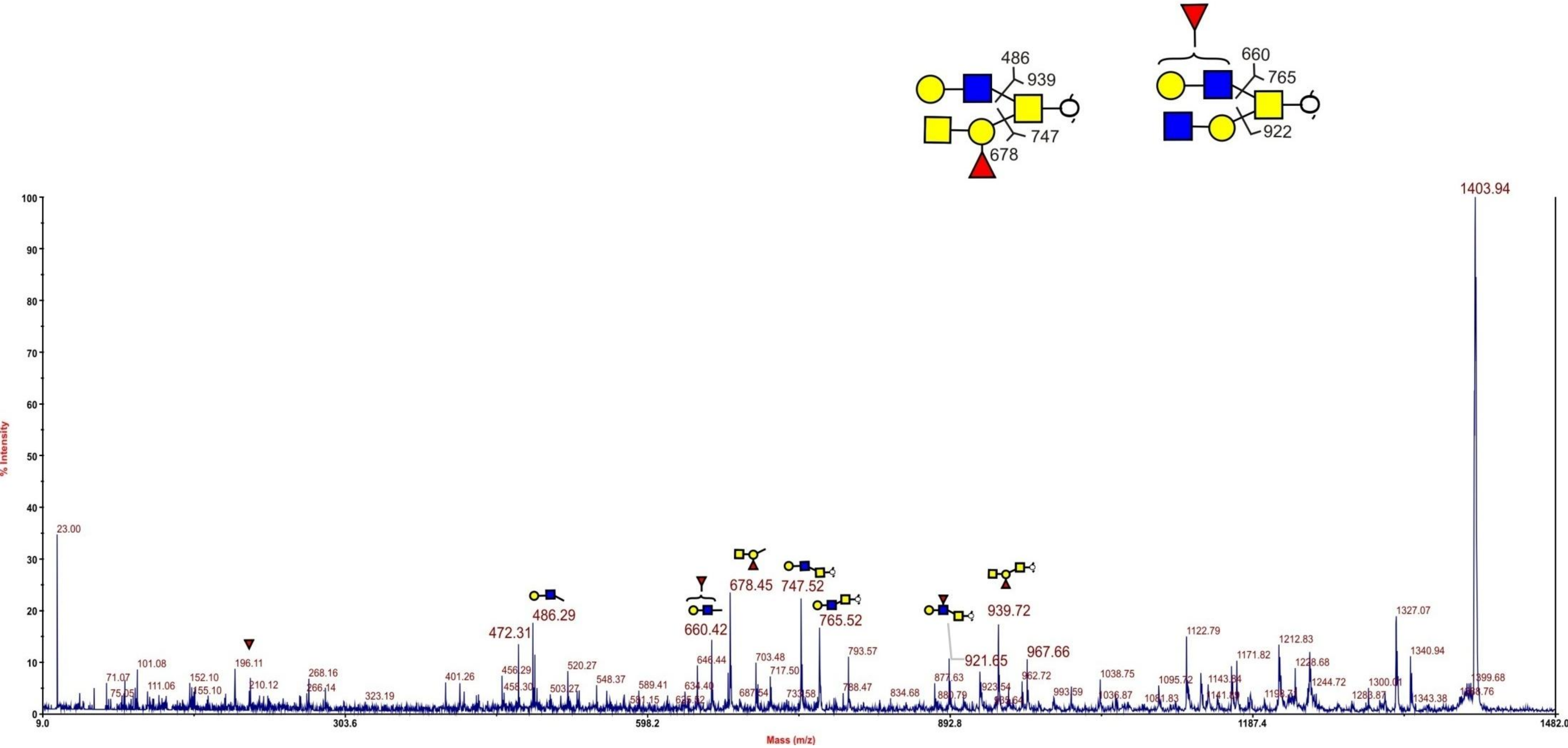

U

Sample 6- msms 1444

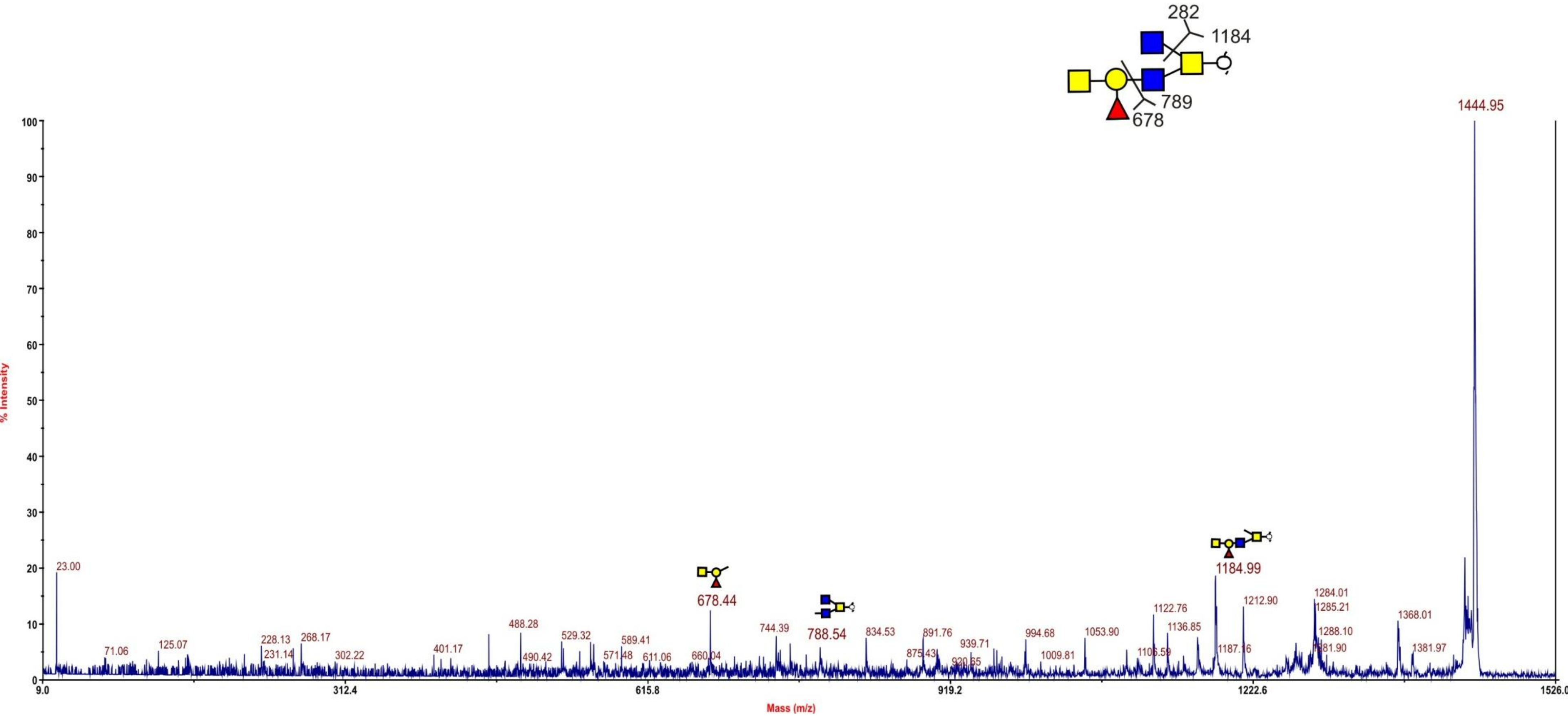

V

Sample 6- msms 1578

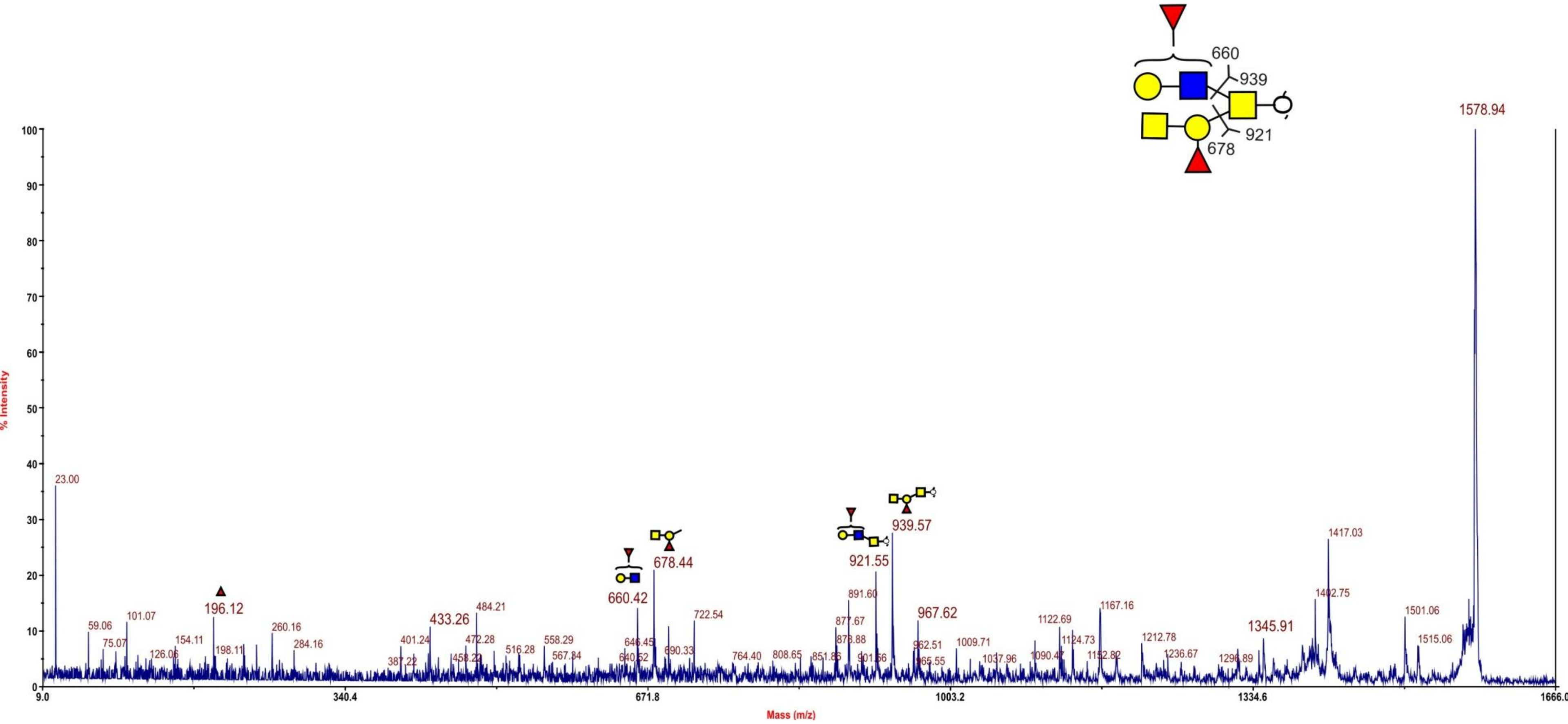

X

Sample 6- msms 1648

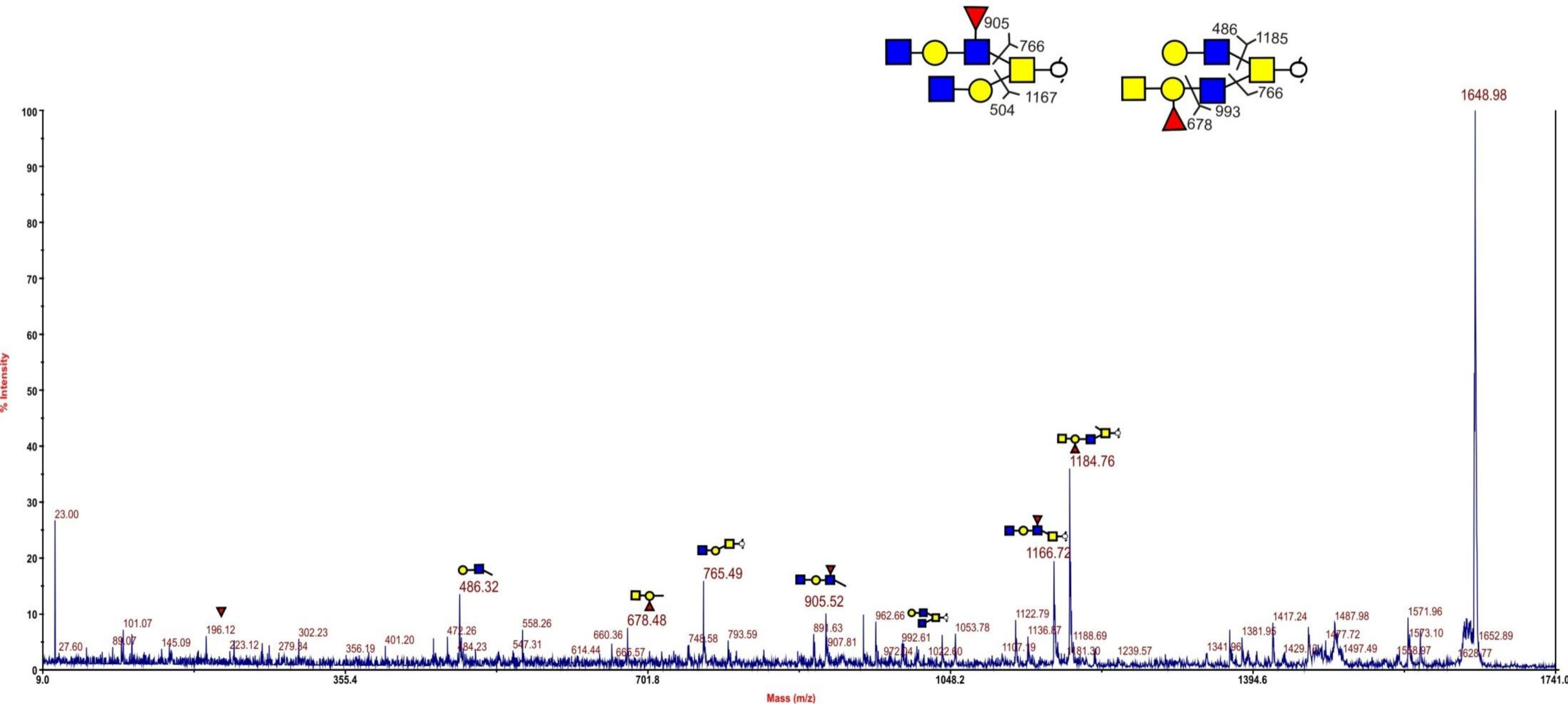

Y

Sample 6- msms 1823

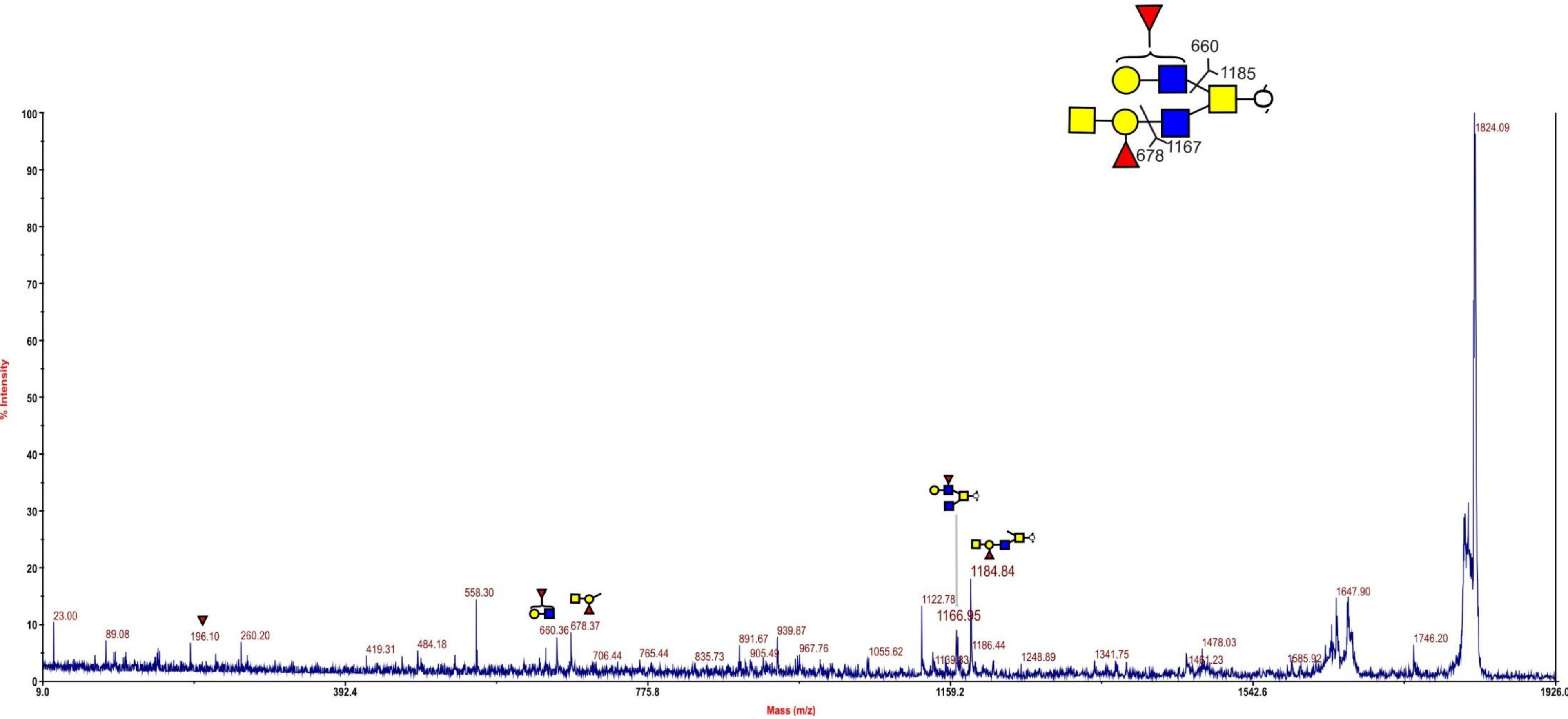

Z
