# Supplementary material for: Histo-Blood Group Antigens Act as Attachment Factors of Rabbit Hemorrhagic Disease Virus Infection in a Virus Strain-Dependent Manner
Source: PLoS Pathog. 2011 Aug 25;7(8):e1002188. doi: 10.1371/journal.ppat.1002188 (PMC3161982; doi:10.1371/journal.ppat.1002188)

Figure S3. MALDI-Ion Trap-TOF MS, MS/MS and MS³ mass spectra of O-glycans released from the Rabbit Duodenum Sample 4. The sample was kindly analyzed by Shimadzu Ltd, by their MALDI-Ion Trap-TOF instrument "Axima Resonance".

A: MS spectrum of sample 4. The annotation of the structure at m/z 1373 takes into account the information emerging from fragmentation data obtained through MS/MS and MS^3 analysis.

B: MS/MS spectrum of peak at m/z 1373. Theoretical fragments, shown at the top- right corner of the figure, match the observed ones obtained by Ion trap-MS/MS analysis, reported on the annotated peaks. Peaks marked with the symbol "X" are due to the matrix used for MALDI analysis.

C: MS^3 spectra of the ion at m/z 1079 obtained after fragmentation of the parent ion at m/z 1373. Theoretical fragments, shown at the top- right corner of the figure, match the observed fragments obtained by Ion trap-MS^3 fragmentation, reported on the annotated peaks. Peaks marked with the symbol "X" are due to the matrix used for MALDI analysis.

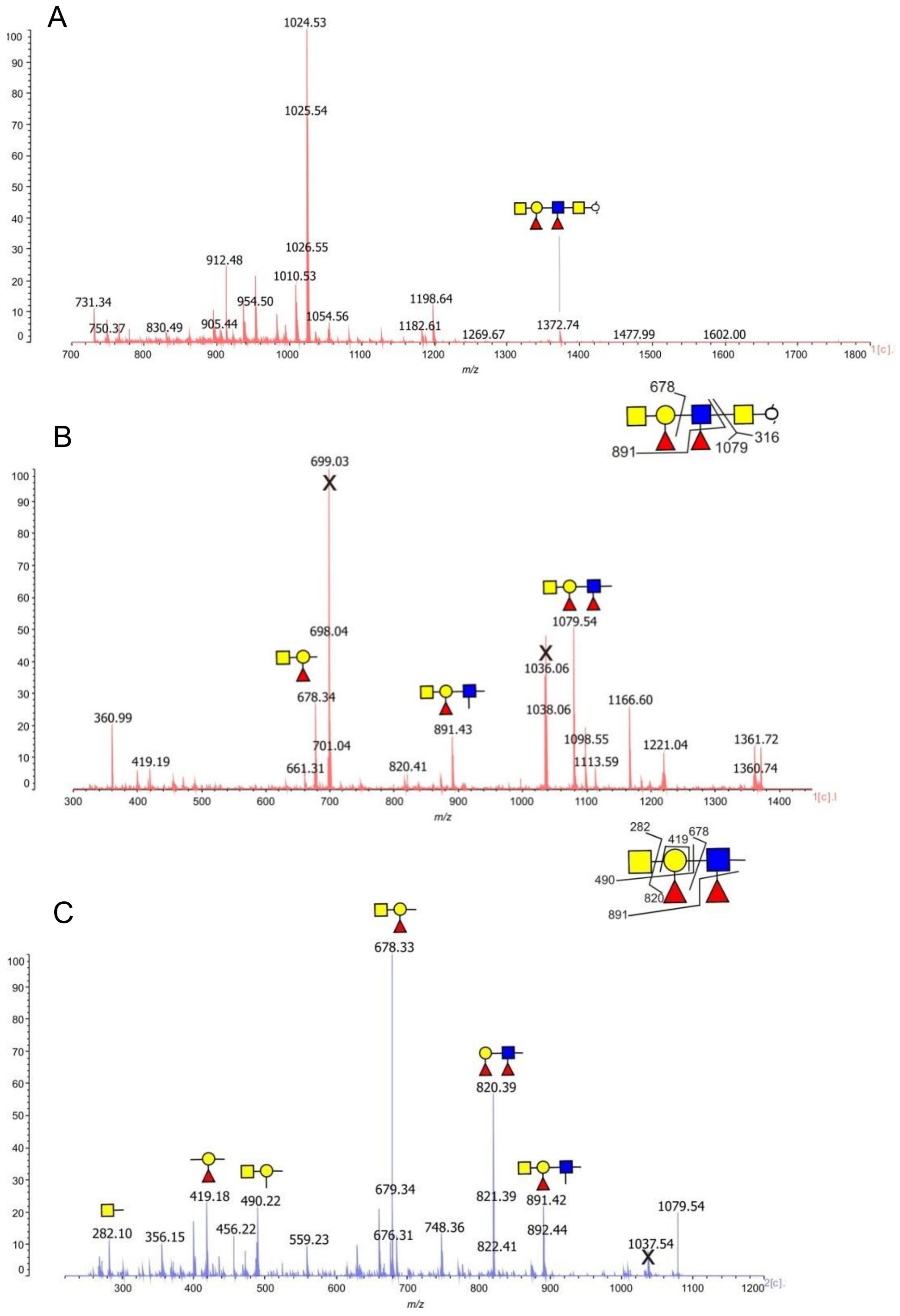

Supplement: Figure S3 — MALDI-Ion Trap-TOF MS, MS/MS and MS3 mass spectra of O-glycans released from the Rabbit Duodenum Sample 4. The sample was kindly analyzed by Shimadzu Ltd, by their MALDI-Ion Trap-TOF instrument “Axima Resonance”. A: MS spectrum of sample 4. The annotation of the structure at m/z 1373 takes into account the information emerging from fragmentation data obtained through MS/MS and MS3 analysis. B: MS/MS spectrum of peak at m/z 1373. Theoretical fragments, shown at the top- right corner of the figure, match the observed ones obtained by Ion trap-MS/MS analysis, reported on the annotated peaks. Peaks marked with the symbol “X” are due to the matrix used for MALDI analysis. C: MS3 spectra of the ion at m/z 1079 obtained after fragmentation of the parent ion at m/z 1373. Theoretical fragments, shown at the top- right corner of the figure, match the observed fragments obtained by Ion trap-MS3 fragmentation, reported on the annotated peaks. Peaks marked with the symbol “X” are due to the matrix used for MALDI analysis. (PDF) [file ppat.1002188.s003.pdf]
