# Supplementary material for: Histo-Blood Group Antigens Act as Attachment Factors of Rabbit Hemorrhagic Disease Virus Infection in a Virus Strain-Dependent Manner
Source: PLoS Pathog. 2011 Aug 25;7(8):e1002188. doi: 10.1371/journal.ppat.1002188 (PMC3161982; doi:10.1371/journal.ppat.1002188)
Supplement: Figure S4 — RHDV challenge of A+B+ and A−B− rabbits. 31 rabbits were infected with an A and B binding G4 strain at 107 or 109 virus copies. Rabbits either succumbed to infection or were sacrificed after 11 days. Duodenum and liver samples were collected at time of death. Duodenum was phenotyped for G4 binding. Duodenum and liver were also assayed for virus RNA via real time RT-PCR. (A–B) Virus RNA of liver or duodenum for 107 infectious dose, (C–D) virus RNA of liver or duodenum for 109 infectious dose, where A+B+ (circle), A−B− (triangle), dead rabbits (black symbol) and survival rabbits (white symbol). (PDF) [file ppat.1002188.s004.pdf]

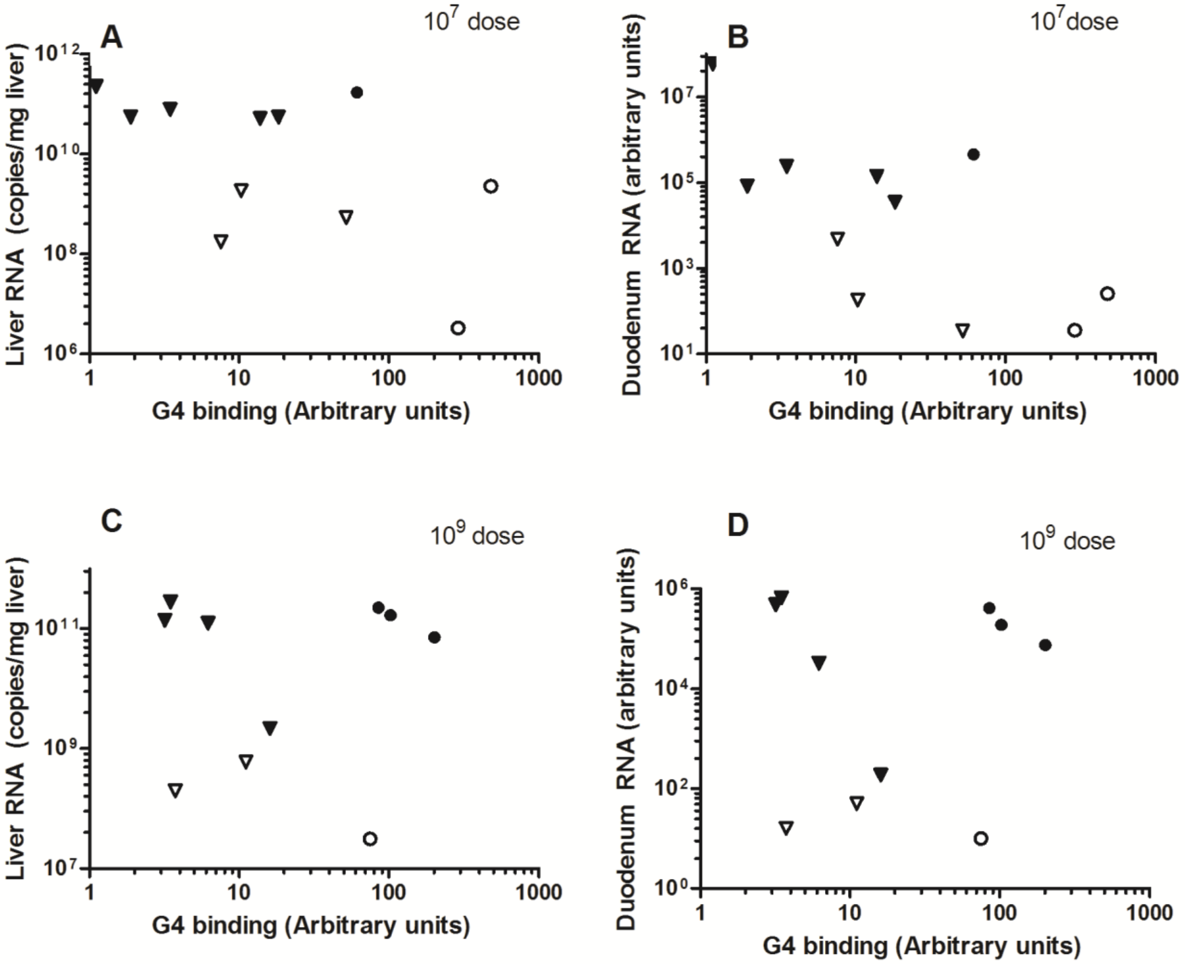

Figure S4. RHDV challenge of A+B+ and A-B- rabbits. 31 rabbits were infected with an A and B binding G4 strain at 10^7 or 10^9 virus copies. Rabbits either succumbed to infection or were sacrificed after 11 days. Duodenum and liver samples were collected at time of death. Duodenum was phenotyped for G4 binding. Duodenum and liver were also assayed for virus RNA via real time RT-PCR. (A-B) Virus RNA of liver or duodenum for 10^7 infectious dose, (C-D) virus RNA of liver or duodenum for 10^9 infectious dose, where A+B+ (circle), A-B- (triangle), dead rabbits (black symbol) and survival rabbits (white symbol).
