# Supplementary material for: Histo-Blood Group Antigens Act as Attachment Factors of Rabbit Hemorrhagic Disease Virus Infection in a Virus Strain-Dependent Manner
Source: PLoS Pathog. 2011 Aug 25;7(8):e1002188. doi: 10.1371/journal.ppat.1002188 (PMC3161982; doi:10.1371/journal.ppat.1002188)
Supplement: Figure S5 — Survival analysis of rabbits challenged with the G4 strain at 3 genome copies doses (105, 107 and 109). At 11 days, all survivors were sacrificed. There were no statistically significant differences between the 3 groups. (PDF) [file ppat.1002188.s005.pdf]

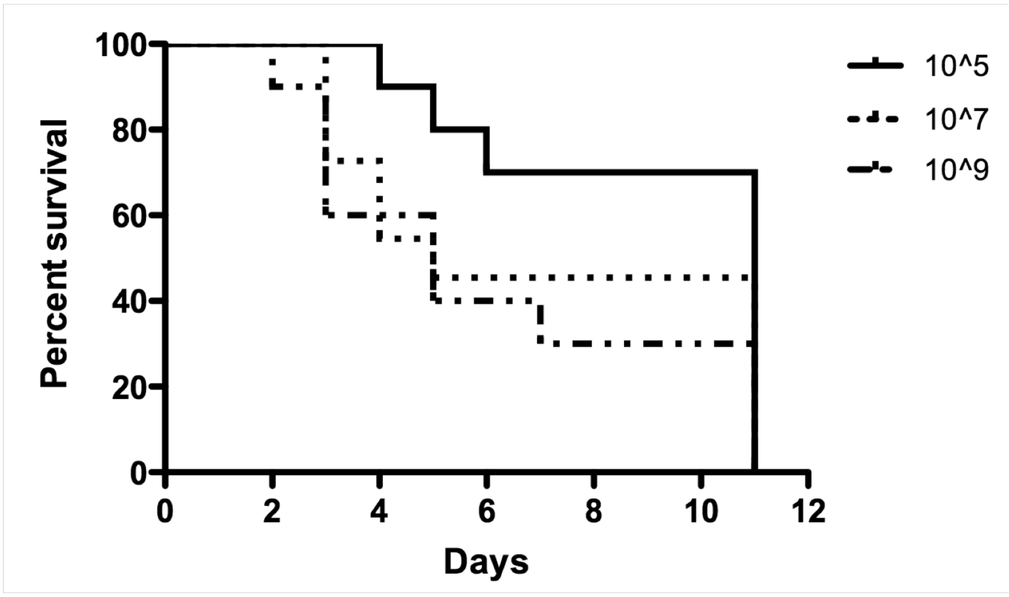

Figure S5. Survival analysis of rabbits challenged with the G4 strain at 3 genome copies doses (10^5, 10^7 and 10^9). At 11 days, all survivors were sacrificed. There were no statistically significant differences between the 3 groups.
