# Supplementary material for: Histo-Blood Group Antigens Act as Attachment Factors of Rabbit Hemorrhagic Disease Virus Infection in a Virus Strain-Dependent Manner
Source: PLoS Pathog. 2011 Aug 25;7(8):e1002188. doi: 10.1371/journal.ppat.1002188 (PMC3161982; doi:10.1371/journal.ppat.1002188)
Supplement: Table S1 — Neoglycoconjugates used to determine the carbohydrate binding characteristics of RHDV strains. aOligosaccharides were used coupled to either polyacrylamide via an 3 carbon spacer (R1), or to human serum albumin via either a p-aminophenylethyl spacer (R2) or an acetyl phenylenediamine spacer (R3). (PDF) [file ppat.1002188.s006.pdf]

Table S1. Neoglycoconjugates used to determine the carbohydrate binding characteristics of RHDV strains

**Trivial name**	**Oligosaccharide structure**[a]
Tn	GalNAca-R1
a-galactose monosaccharide	Gala-R1
a-fucose monosaccharide	Fuca-R1
A disaccharide	GalNAca3Galb-R1
B disaccharide	Gala3Galb-R1
	Gala2Galb-R1
	Gala6Glcb-R1
Core 5	GalNAca3GalNAca-R1
H disaccharide	Fuca2Galb-R1
Forsmann disaccharide	GalNAca3GalNAcb-R1
Core 8	Gala3GalNAca-R1
Type 2 precursor	Galb4GlcNAcb-R1
Tab	Gala3GalNAcb-R1
A trisaccharide	GalNAca3(Fuca2)Galb-R1, R2
B trisaccharide	Gala3(Fuca2)Galb-R1, R2
H type 1	Fuca2Galb3GlcNAcb-R1
H type 2	Fuca2Galb4GlcNAcb-R1, R2
H type 3	Fuca2Galb3GalNAca-R1
aGal trisaccharide	Gala3Galb4GlcNAcb-R1
Gb3 (Pk)	Gala4Galb4Glcb-R1
iGb3	Gala3Galb4Glcb-R1
P1 trisaccharide	Gala4Galb4GlcNAcb-R1
Lewis a	Galb3(Fuca4)GlcNAcb-R1
Lewis x	Galb4(Fuca3)GlcNAcb-R1
3’-Sulfo-Lewis a	Su-O-3Galb3(Fuca4)GlcNAcb-R1
3’-Sulfo-Lewis x	Su-O-3Galb4(Fuca3)GlcNAcb-R1
A type 2	GalNAca3(Fuca2)Galb4GlcNAcb-R1
B type 2	Gala3(Fuca2)Galb4GlcNAcb-R1
Lewis b	Fuca2Galb3(Fuca4)GlcNAcb-R1, R3
Lewis y	Fuca2Galb4(Fuca3)GlcNAcb-R1, R2
Sialyl-Lewis a	NeuAca2,3Galb3(Fuca4)GlcNAcb-R1, R3
Sialyl-Lewis x	NeuAca2,3Galb3(Fuca4)GlcNAcb-R1, R3
6-sulfo Sialyl-Lewis x	NeuAca2,3Galb3(Fuca4)(Su-O-6)GlcNAcb-R1
Tk	GlcNAcb3(GlcNAcb6)GlcNAcb3Galb-R1
Lacto-N-tetraose (LNT)	Galb3GlcNAcb3Galb4Glcb-R3
Lacto-N-neotetraose (LNnT)	Galb4GlcNAcb3Galb4Glcb-R3
a-Gal-Lewis x	Gala3Galb4(Fuca3)GlcNAcb-R1
a-Gal pentasaccharide	Gala3Galb4GlcNAcbGalb4Glcb-R1
Sialyl-Lewis x pentasaccharide	NeuAca2,3Galb3(Fuca4)GlcNAcb3Galb-R1
Sialyl-lacto-N-neotetraose (Sia-LNnT)	NeuAca2,3Galb4GlcNAcb3Galb4Glcb-R1

Lactoneofucopentaose I (LNF I)	Fuca2Galb3GlcNAcb3Galb4Glcb-R1, R3
Lacto-N-fucopentaose II (LNF II)	Galb3(Fuca4)GlcNAcb3Galb4Glcb-R3
Lacto-N-fucopentaose III (LNF III)	Galb4(Fuca3)GlcNAcb3Galb4Glcb-R3
A hexasaccharide	GalNAca3(Fuca2)Galb3GlcNAcb3Galb4Glcb-R3
A heptasaccharide	GalNAca3(Fuca2)Galb3(Fuca4)GlcNAcb3Galb4Glcb-R3

[a] Oligosaccharides were used coupled to either polyacrylamide via an 3 carbon spacer (R1), or to human serum albumin via either a p-aminophenylethyl spacer (R2) or an acetyl phenylenediamine spacer (R3).
