# Supplementary material for: Histo-Blood Group Antigens Act as Attachment Factors of Rabbit Hemorrhagic Disease Virus Infection in a Virus Strain-Dependent Manner
Source: PLoS Pathog. 2011 Aug 25;7(8):e1002188. doi: 10.1371/journal.ppat.1002188 (PMC3161982; doi:10.1371/journal.ppat.1002188)
Supplement: Table S2 — Contingency table of relationship between expression of H type 2 with A or B. 1p = 4.8×10^−7^; 2p = 7.5×10^−6^ ; 3p = 2.0×10^−9^. (PDF) [file ppat.1002188.s007.pdf]

Table S2. Contingency table of relationship between expression of H type 2 with A or B expression for all individual rabbits

	A+[1]	A-[1]	B+[2]	B-[2]	A+B+[3]	A-B-[3]	A+B-[3]
H+	16	32	15	33	15	32	1
H-	36	0	29	7	29	0	7

Rabbits were phenotyped for expression or lack of expression of A, B and H antigens (+ or -) and grouped according to A or B phenotypes versus H phenotypes. Values represent the number of animals in each subgroup. Expressors of A and B are significantly more often H- than H+ due to the masking of H epitopes by the A or B epitopes.

[1] $p=4.8x10^{-7}$

[2] $p=7.5x10^{-6}$

[3] $p=2.0x10^{-9}$
