# Supplementary material for: Histo-Blood Group Antigens Act as Attachment Factors of Rabbit Hemorrhagic Disease Virus Infection in a Virus Strain-Dependent Manner
Source: PLoS Pathog. 2011 Aug 25;7(8):e1002188. doi: 10.1371/journal.ppat.1002188 (PMC3161982; doi:10.1371/journal.ppat.1002188)
Supplement: Table S3 — Summary of all structures observed in the MALDI-TOF-MS spectra of the duodenum tissue from rabbits, Samples 1–10. The symbol presentation of O-glycans is based on the nomenclature used in Essentials of Glycobiology textbook (http://www.ncbi.nlm.nih.gov/books/NBK1908) and adopted by the Consortium for Functional Glycomics (http://www.functionalglycomics.org). Mass values [M + Na]+, cartoon representation of structure and epitope types are reported for each peak detected. Intensity of the peaks observed is reported for each sample analyzed. ND = not detected – = very minor component (<25%); - = minor component (25–50%); + = major component (>25%); ++ = very major component (25–50%); +++ = most abundant component (>50%). (PDF) [file ppat.1002188.s008.pdf]

Table S3. Summary of all structures observed in the MALDI-TOF-MS spectra of the duodenum tissue from rabbits, Samples 1-10. The symbol presentation of O-glycans is based on the nomenclature used in Essentials of Glycobiology textbook (http://www.ncbi.nlm.nih.gov/books/NBK1908) and adopted by the Consortium for Functional Glycomics (http://www.functionalglycomics.org). Mass values [M + Na]+, cartoon representation of structure and epitope types are reported for each peak detected. Intensity of the peaks observed is reported for each sample analyzed:

Mass	Structure	Epitope	Sample									
			1	2	3	4	5	6	7	8	9	10
708	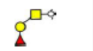	H	ND	++	+	++	ND	+	-	ND	ND	++
912	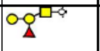	B	+++	ND	ND	+	+	+	ND	+	+	ND
954	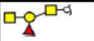	A	+++	ND	ND	-	-	++	+	-	-	--
	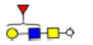	H or Lewis X	ND	+++	+	+	++	-	-	+	+	+
	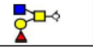	H	ND	-	-	ND	--	ND	ND	--	ND	-
1128	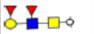	Lewis Y	ND	+	+	ND	+	+	ND	ND	ND	+
1158	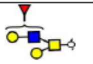	H or Lewis X	ND	ND	ND	ND	ND	+	ND	ND	ND	-
	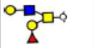	H	ND	ND	ND	ND	ND	-	ND	ND	ND	+
1199	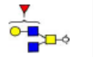	H or Lewis X	ND	+	ND	--	--	ND	ND	ND	ND	ND
	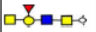	A	ND	ND	ND	++	++	+++	+	ND	+	ND
	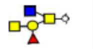	A	ND	ND	ND	-	-	-	ND	ND	ND	ND
1332	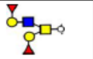	H	ND	+	ND	ND	ND	+	ND	ND	ND	++
1362	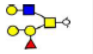	B	+	ND	ND	ND	ND	ND	ND	ND	ND	ND
1373	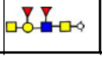	A	ND	ND	ND	+	+	+	ND	ND	ND	ND
1403	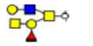	A	++	+	-	ND	ND	++	ND	ND	ND	ND
	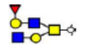	H	-	-	ND	ND	ND	ND	ND	ND	ND	ND
	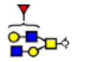	H or Lewis X	ND	ND	+	ND	ND	-	ND	ND	ND	ND
	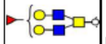	H or Lewis X	--	--	--	ND	ND	ND	ND	ND	ND	ND
1444	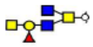	A	ND	ND	ND	ND	ND	+	ND	ND	ND	ND
1577	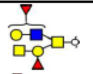	A and Lewis X/H	ND	-	+	ND	ND	+	ND	ND	ND	ND
	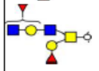	H and/or Lewis X	ND	+	ND	ND	ND	ND	ND	ND	ND	ND
	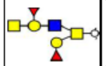	A and H	ND	+	ND	ND	ND	ND	ND	ND	ND	ND
1648	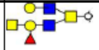	A	ND	ND	ND	ND	ND	+	ND	ND	ND	ND
1822	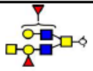	A and Lewis X/H	ND	ND	ND	ND	ND	+	ND	ND	ND	ND

ND = not detected
— — = very minor component (<25%)
— = minor component (25-50%)
+ = major component (> 25%)
++ = very major component (25-50%)
+++ = most abundant component (>50%)
