# Supplementary material for: Histo-Blood Group Antigens Act as Attachment Factors of Rabbit Hemorrhagic Disease Virus Infection in a Virus Strain-Dependent Manner
Source: PLoS Pathog. 2011 Aug 25;7(8):e1002188. doi: 10.1371/journal.ppat.1002188 (PMC3161982; doi:10.1371/journal.ppat.1002188)
Supplement: Table S4 — Composition of permethylated O-glycans in 10 samples of Rabbit Duodenum, obtained through MALDI-TOF analysis. (PDF) [file ppat.1002188.s009.pdf]

Table S4: Composition of permethylated O-glycans in 10 samples of Rabbit Duodenum, obtained through MALDI-TOF analysis.

m/z value	Composition
708	HexNAc, Hex, Fuc
912	HexNAc , Hex2, Fuc
954	HexNAc 2, Hex, Fuc
995	HexNAc 3, Fuc
1128	HexNAc 2, Hex, Fuc2
1158	HexNAc 2, Hex2, Fuc
1199	HexNAc 3, Hex, Fuc
1332	HexNAc 2, Hex2, Fuc2
1362	HexNAc 2, Hex3, Fuc
1373	HexNAc 3, Hex, Fuc2
1403	HexNAc 3, Hex2, Fuc
1444	HexNAc 4, Hex, Fuc
1577	HexNAc 3, Hex2, Fuc2
1648	HexNAc 4, Hex2, Fuc
1822	HexNAc 4, Hex2, Fuc2
