# Supplementary material for: Histo-Blood Group Antigens Act as Attachment Factors of Rabbit Hemorrhagic Disease Virus Infection in a Virus Strain-Dependent Manner
Source: PLoS Pathog. 2011 Aug 25;7(8):e1002188. doi: 10.1371/journal.ppat.1002188 (PMC3161982; doi:10.1371/journal.ppat.1002188)
Supplement: Table S5 — Significance of correlation between RHDV binding and ABH phenotypes with Fischer's exact test. 1 7 rabbits A+B-H− and one A+B-H+ with weak H type 2 expression. (PDF) [file ppat.1002188.s010.pdf]

Table S5. Significance of correlation between RHDV binding and ABH phenotypes with Fischer's exact test.

Strain	Binding	A+B+H+	A+B+H-	A+B-[1]	A-B-H+	p value
G2	Low	3	10	7	8	0.014
	Medium	6	12	1	9	
	High	6	7	0	15	
G3	Low	5	10	2	10	0.43
	Medium	3	10	1	14	
	High	6	9	5	8	
G4	Low	1	7	1	19	$3x10^{-8}$
	Medium	3	12	0	13	
	High	11	10	7	0	
G5	Low	2	5	2	19	0.0011
	Medium	5	12	1	10	
	High	8	12	5	3	
G6	Low	3	11	1	11	$9.8x10^{-4}$
	Medium	3	7	0	16	
	High	7	10	7	3	
G1	Low	3	5	3	17	0.013
	Medium	4	12	1	11	
	High	8	12	4	4	

[1] 7 rabbits A+B-H- and one A+B-H+ with weak H type 2 expression
